# New Ethylated Derivatives of Sulfur- and Nitrogen-Containing Artifacts from *Tenodera sinensis* Egg Pod and Their Anti-Renal Fibrosis

**DOI:** 10.3390/molecules29153491

**Published:** 2024-07-25

**Authors:** Ye-Fei Chen, Shi-Gang Peng, Yong-Ming Yan, Yong-Xian Cheng

**Affiliations:** 1 Guangdong Provincial Key Laboratory of Chinese Medicine Ingredients and Gut Microbiomics, Institute for Inheritance-Based Innovation of Chinese Medicine, Marshall Laboratory of Biomedical Engineering, School of Pharmacy, Shenzhen University Medical School, Shenzhen University, Shenzhen 518055, China; chenyefei@szu.edu.cn (Y.-F.C.); pengsg18884367820@163.com (S.-G.P.); yanym@szu.edu.cn (Y.-M.Y.); 2 Guangdong Key Laboratory for Biomedical Measurements and Ultrasound Imaging, National-Regional Key Technology Engineering Laboratory for Medical Ultrasound, School of Biomedical Engineering, Shenzhen University Medical School, Shenzhen 518060, China; 3 School of Pharmacy, Chengdu University of Traditional Chinese Medicine, Chengdu 611137, China

**Keywords:** *Mantidis ootheca*, *Tenodera sinensis* Saussure, mantidisamides E–H, renal fibrosis

## Abstract

Three pairs of enantiomers and one achiral molecule that are new ethylated derivatives of sulfur and nitrogen-containing compounds named mantidisamides E–H (**1**–**4**), along with twenty known ones (**5**–**24**), were derived from the ethanol extract of *Tenodera sinensis* Saussure. The structures of these new compounds and their absolute configurations were assigned on the basis of spectroscopic analyses and computational methods. The assessment of activities in NRK-52e cells induced by TGF-β1 demonstrated that the previously undescribed compounds **1** and **2** exhibited a significant capacity to inhibit the expression of proteins (fibronectin, collagen I, and *α*-SMA). Moreover, the biological activity of these compounds was found to increase with rising concentrations. Notably, compounds **1**–**4** should be artifacts; however, undescribed compounds **1** and **2,** which possessed obvious biological activity, might be attractive for chemists and biologists due to the potential for more detailed exploration of their properties. It is worth mentioning that compounds **1** and **2** remain novel structures even in the absence of the ethoxy group.

## 1. Introduction

**Figure 1:**
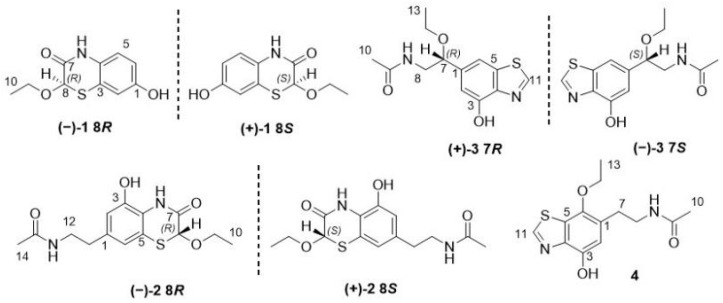
Structures of compounds **1**–**4**.

For centuries, it has been recognized that insects and their products serve as valuable sources of sustenance and medicine, owing to their nutritive properties, diverse chemical compositions, and abundance. Our present research endeavors are focused on investigating the natural substances obtained from *Mantidis ootheca*, which originated from three species of Mantis: *Tenodera sinensis* Saussure, *Statilia maculate* (Thurlberg), and *Hierodula patellifera* (Serville) [1]. The *Mantidis ootheca* is a very common insect that is mainly distributed in Guangxi, Yunnan, and Liaoning provinces of China, and its potential for therapeutic use in the treatment of kidney diseases has been noted [2,3]. In our previous studies, we have reported some interesting small molecules that exhibited COX-2 inhibitory and anti-renal fibrosis activities [4,5]. In our quest for a more profound comprehension of the chemical composition and biological activity of *Mantidis ootheca*, we kept on conducting the study on the specified insect in the title, which resulted in the discovery of three pairs and one previously undescribed compound containing sulfur and nitrogen atoms (Figure 1). Additionally, we have identified twenty previously known compounds (Figure S1). Their structures, including absolute configurations, were determined through a combination of spectroscopic and computational methods. Moreover, in order to assess the biological effects of the undescribed compounds, we investigated their possible anti-fibrotic properties, a pathology characterized by the abnormal accumulation of extracellular matrix (ECM) proteins within the renal system.

## 2. Results and Discussion

### 2.1. Structure Elucidation of the Compounds

**Figure 2:**
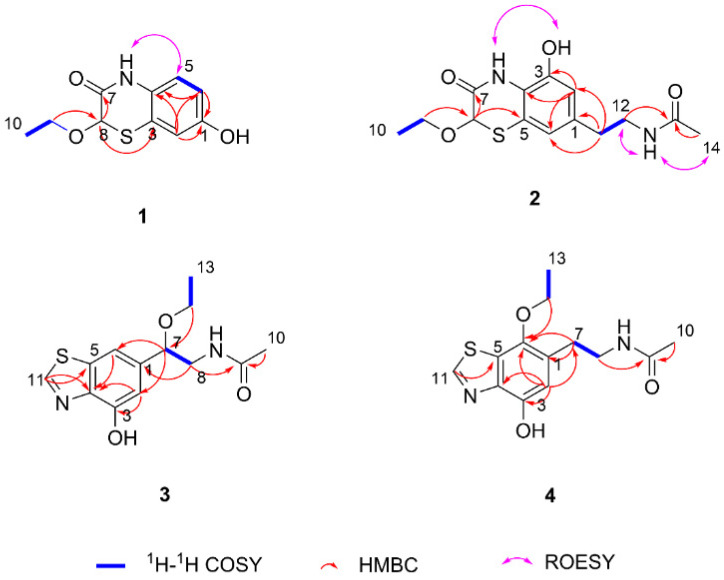
Key ^1^H–^1^H COSY and HMBC correlations of compounds **1**–**4** and ROESY correlations of compounds **1** and **2**.

**Figure 3:**
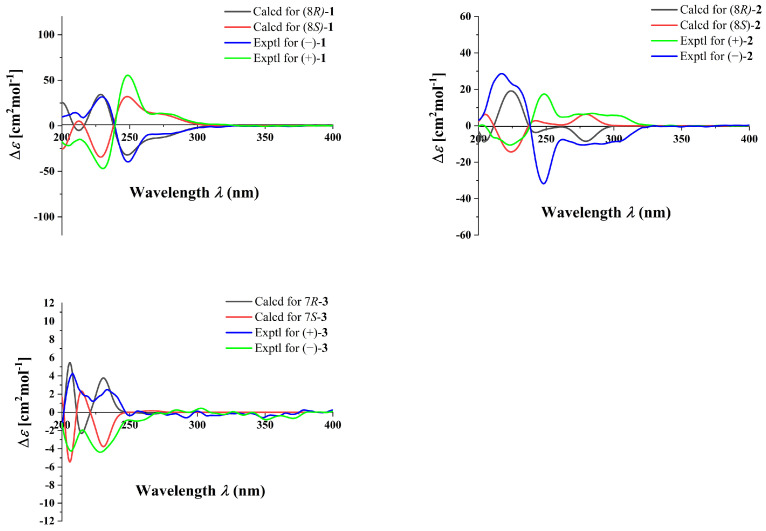
Calculated and experimental ECD spectra of compounds **1**–**3**.

Compound **1** ((±) mantidisamide E) was isolated as a yellow powder. The molecular formula was confirmed to be C_10_H_11_NO_3_S (*m*/*z* 248.0353 [M + Na]^+^, calcd for C_10_H_11_NO_3_SNa, 248.0352), with six degrees of unsaturation, by analyzing its HRESIMS, ^13^C NMR, and DEPT spectra. The ^1^H NMR spectrum (Table 1) indicates that there is an ABX spin system [*δ*_H_ 6.66 (dd, *J* = 8.6, 2.6 Hz, H-6), 6.74 (d, *J* = 2.6 Hz, H-2), 6.85 (d, *J* = 8.6 Hz, H-5)]. The proton signals [*δ*_H_ 3.83 (dq, *J* = 9.5, 7.0 Hz, Ha-9), 3.52 (dq, *J* = 9.5, 7.0 Hz, Hb-9), 1.12 (t, *J* = 7.0 Hz, H_3_-10)] combing with the ^13^C NMR data of C-9 (*δ*_C_ 65.1) and C-10 (*δ*_C_ 14.9) exhibit the presence of one ethoxy group. The structure of **1** was accomplished mainly by 2D NMR spectroscopic data (Figure 2). The HMBC spectrum shows the correlations (Figure 2) of H_2_-9 (*δ*_Ha_ 3.83, *δ*_Hb_ 3.52)/C-8 (*δ*_C_ 79.5), demonstrating that the ethoxy group is located at C-8. The HMBC correlations of H-8 (*δ*_H_ 5.02)/C-3 (*δ*_C_ 118.7) and C-7 (*δ*_C_ 163.6), combined with the molecular formula, reveal that C-7 is connected to C-8 and C-8 is supposed to be linked with C-3 via a sulfur atom. Furthermore, the ROESY correlation (Figure 2) of 7-*N*H (*δ*_H_ 10.54)/H-5 (*δ*_H_ 6.83) indicates that C-7 is connected to C-4 via an -*N*H- bridge. Thus, the planar structure of **1** was deduced. In addition, the near-zero optical rotation indicates **1** may be a racemate, and then it was submitted to a chiral HPLC to afford two enantiomeric isomers (±)-**1**. Then, the ECD calculations were performed at the mPW1PW91/6-311+G(d,p) level with the PCM model in methanol. The ECD spectra were finished by comparing the experimental ECD curves with the calculated ones, and the results showed that the calculated ECD spectrum for compound **1** matches well with the experimental one (Figure 3). Thus, the absolute configurations were determined to be 8*S* for (+)-**1** and 8*R* for (−)-**1**.

Compound **2** ((±) mantidisamide F) was obtained as a yellow powder. Its molecular formula C_14_H_18_N_2_O_4_S was deduced on the basis of negative HRESIMS at *m*/*z* 309.0916 [M–H]^−^ (calcd for C_14_H_17_N_2_O_4_S, 309.0915), ^13^C NMR, and DEPT spectra, indicating seven degrees of unsaturation. In the ^1^H NMR spectrum, two methyl protons [*δ*_H_ 1.12 (t, *J* = 7.0 Hz, H_3_-10), 1.91 (s, H_3_-14)], three methylene protons [*δ*_H_ 2.67 (m, H_2_-11), 3.35 (m, H_2_-12), 3.53 (dq, *J* = 9.5, 7.0 Hz, Ha-9), and 3.83 (dq, *J* = 9.5, 7.0 Hz, Hb-9)], three methine protons [*δ*_H_ 5.05 (s, H-8), 6.61 (d, *J* = 1.7 Hz, H-2), 6.67 (d, *J* = 1.7 Hz, H-6)], and three active proton signals [*δ*_H_ 7.88 (t, *J* = 5.4 Hz, 12-*N*H), 9.60 (brs, 3-OH), 10.09 (brs,7-*N*H)] were observed (Table 1). The ^13^C NMR and DEPT spectra of **2** (Table 1) indicate 14 carbon signals, comprising two methyls, three methylenes, three methines (one nitrogen or oxygen-bearing and two olefinic), and six nonprotonated carbons (two carbonyls and four olefinic). A detailed comparison of 1D NMR data between compounds **1** and **2** indicated that both of them have the same basic structural fragment of benzothiazide. The ^1^H-^1^H COSY correlation of H_2_-11/H_2_-12 and the HMBC correlations of H_2_-11/C-2 (*δ*_C_ 114.4), C-6 (*δ*_C_ 120.2), and C-1 (*δ*_C_ 136.6) reveal that C-11 is connected to C-1. The HMBC correlations of H_2_-12 and H_3_-14/C-13 (*δ*_C_ 173.3), combined with the ROESY correlations (recorded in DMSO-*d*_6_, Figure 2) of H_2_-12 (*δ*_H_ 3.18)/12-*N*H (*δ*_H_ 7.88)/H_3_-14 (*δ*_H_ 1.77), exhibit the presence of an acetamide group located at C-12 via the *N* atom. A hydroxyl group at C-3 can be deduced by the chemical shift of C-3 at *δ*_C_ 145.3 and the observed ROESY correlation (Figure 2) of 7-*N*H (*δ*_H_ 10.09)/3-OH (*δ*_H_ 9.60). The presence of one ethoxy group located at C-8 (*δ*_C_ 79.5) in **2** is supported by the particular proton signals [*δ*_H_ 3.83 (dq, *J* = 9.5, 7.0 Hz, Ha-9), 3.52 (dq, *J* = 9.5, 7.0 Hz, Hb-9), 1.12 (t, *J* = 7.1 Hz, H_3_-10)] and the HMBC correlations of H_2_-9/C-8. Therefore, the planar structure of **2** was established. Similar to **1**, compound **2** was found to be racemic; its chiral HPLC chromatographic separation resulted in a pair of enantiomers (+)-**2** and (−)-**2**. Additionally, ECD calculations of (8*R*)-**2** were carried out based on the B3LYP/6-31G (d, p) level with PCM in methanol. As shown in Figure 3, the experimental ECD curve of (+)-**2** is similar to the calculated one of (8*S*)-**2**. Thus, we confirmed that the absolute configurations of (+)-**2** and (−)-**2** are *S*-form and *R*-form, respectively.

Compound **3** ((±) mantidisamide G), a colorless gum, has the molecular formula of C_13_H_16_N_2_O_3_S by analysis of its negative HRESIMS at m/z 279.0808 [M–H]^−^ (calcd for C_13_H_15_N_2_O_3_S, 279.0809), with 7 degrees of unsaturation. The ^13^C NMR combined with the DEPT 135 experimental data of **3** (Table 2) show 13 carbon signals attributed to 2 methyls, 2 methylenes, 4 methines, and 5 nonprotonated carbons (containing one carbonyl). A detailed analysis of the NMR data suggested that the structure of **3** is similar to that of polyrhadopamine D [6], except for an additional ethoxy group at C-7 in **3**, evidenced by the ^1^H-^1^H COSY correlation of H_2_-12 (δ_H_ 3.44)/H_3_-13 (δ_H_ 1.20) (Figure 2), and the HMBC correlation of H_2_-12/C-7 (δ_C_ 81.5) (Figure 2). Thus, the planar structure of **3** was identified. Two enantiomers were gained from **3** by means of chiral HPLC purification, and their absolute configurations were assigned as 7S for (−)-**3** and 7R for (+)-**3** by comparing the calculated ECD spectra with the experimental ones (Figure 3).

Compound **4** (mantidisamide H), a colorless gum, has the molecular formula of C_13_H_16_N_2_O_3_S, deduced by analysis of its negative HRESIMS at *m*/*z* 279.0813 [M–H]^−^ (calcd for C_13_H_15_N_2_O_3_S, 279.0809), with 7 degrees of unsaturation. Careful comparison of the NMR data of **4** with those of **3** (Table 2) reveals that they have similar structures differing in that the ethoxy group in **4** is located at C-6, which was demonstrated by the HMBC correlation of H-2 (*δ*_H_ 6.67)/C-6 (*δ*_C_ 157.2) and H_2_-12 (*δ*_H_ 4.11)/C-6 (Figure 2). Therefore, the planar structure of **4** was assigned and named mantidisamide H.

Twenty known compounds (Figure S1), which included polyrhadopamine D (**5**) [6], divesamide A (**6**) [7], *N*-acylplancyidopamine A (**7**) [8], tenoderin B (**8**) [9], (2*R*,3*S*)-2-(3′,4′-dihydroxyphenyl)-3-acetylamino-7-(*N*-acetyl-2′′-aminoethylene)-1,4-benzodioxane (**9**) [10], indole 3-acetic acid (**10**) [11], dihydroxyisoechinulin A (**11**) [12], circumdatin G **(12)** [13], ginkgool (**13**) [14], rel-(2*α*,3*β*)-7-O-methylcedrusin **(14**) [15], (+)-isolariciresinol (**15**) [16], (+)-cycloolivil (**16**) [17], (−)-secoisolariciresinol (**17**) [18], umbelliferone (**18**) [19], fraxetin (**19**) [20], cyclo-(Phe-Tyr) (**20**) [21], 3-(2-(4-hydroxyphenyl)-2-oxoethyl)-5,6-dihydropyridin-2(1H)-one (**21**) [22], cristatumside A (**22**) [23], aspongopusin (**23**) [24], and yangjinhualine A (**24**) [25], were obtained simultaneously from *T. sinensis* and structurally identified by comparison of NMR data with previous studies.

Of note, compounds **1**–**4** all contain an ethoxyl group in the structures. Normally, this change could be considered to be artificial in nature during extraction or isolation procedures. In this study, we proposed that **1**–**4** are also artifacts during the extraction process since EtOH was used for extraction. In spite of this, we still changed the alterative solvents, including acetone and MeOH, to extract from raw materials under mild conditions. Then, we made an effort to examine whether **1**–**4** are present or not by HPLC-MS experiments. As expected, **1**–**4** were not detectable in the above extracts (Figures S44–S47), suggesting that they should be artificial products. Despite this, it will have no negative influence to add the structural or biological diversity of such types of structures where the biological activity has been confirmed by the following experiments in this study (see below). More significantly, it has been determined that the structures of compounds 1 and 2 are novel even without the ethoxy group.

**Scheme 1:**
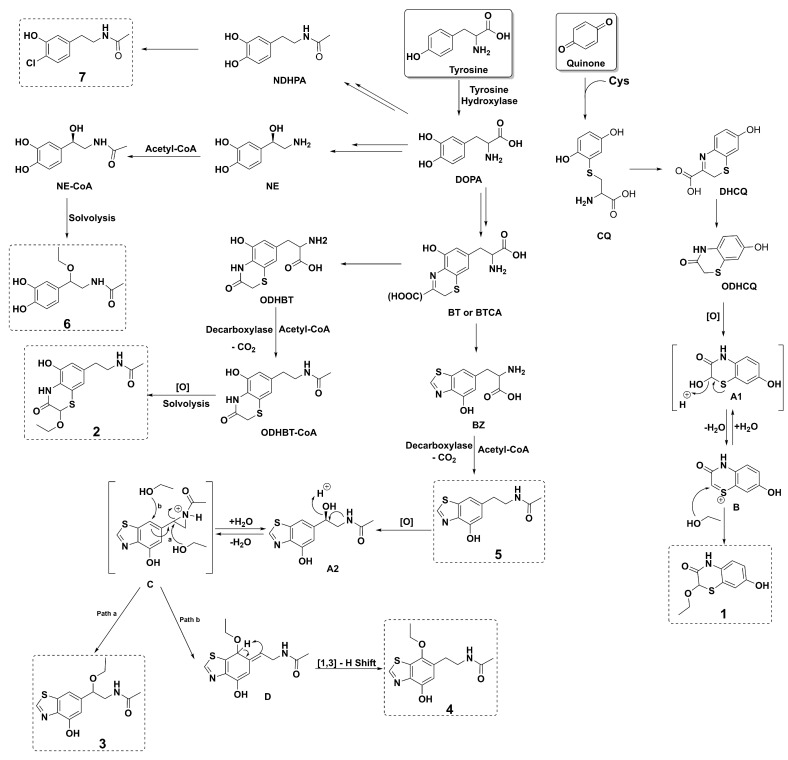
Proposed biosynthetic pathway of compounds **1**–**7**.

In order to understand the sources of these molecules, the biosynthetic pathways of compounds **1**–**7** have been discussed (Scheme 1). The proposed biosynthetic pathway of the mantidisamides commenced with tyrosine, which was converted to DOPA, followed by norepinephrine (NE) [26], 1,4-benzothiazine (BT) or benzothiazine carboxylic acid (BTCA) [27], and *N*-(3,4-dihydroxyphenethyl) acetamide (NDHPA) through several enzymatic transformations. The unstable intermediates, BT and BTCA, may possibly lead to the formation of 3-oxo-3,4-dihydro-1,4-benzothiazine (ODHBT), and the benzothiazole (BZ). Furthermore, ODHBT decarboxylated followed by acetylation with acetyl-CoA led to the formation of ODHBT-CoA. Finally, ODHBT-CoA was then converted to compound **2** through oxidation by a hydroxylase and subsequently ethylated. BZ decarboxylated and acylated to afford **5.** Compound **5** transformed into a non-isolated precursor **A2** through oxidation, prone to dehydration under acidic conditions or when extracted as mixtures with acidic co-metabolites to produce an aziridine intermediate **C**, which was a nucleophilic addition with EtOH at C-7 and C-6 positions to generate the compounds **3** and **4,** respectively. In addition, racemic artifacts formed due to nucleophile (EtOH) attacks from the top side and bottom side at the planner carbocation position; in particular, EtOH attacked from both sides at C-7 positions of aziridine intermediate **C** to generate the racemic compound **3.** NE was acylated with acetyl-CoA and followed by ethylation to obtain compound **6**. The hydroxyl group in NDHPA was substituted with a chlorine atom to form **7**. The biosynthesis of compound **1** began with quinone, which was converted to 5-*S*-cysteinyl quinone (CQ) via a rapid addition reaction. CQ underwent intramolecular cyclization by condensation of the pendant amine, affording 2*H*-1,4-benzothiazine (DHCQ) [28]. Furthermore, decarboxylation and oxidation introduce a hydroxy group to the obtained **A1**. **A1** is accelerated to dehydration to produce the achiral thionium intermediate **B**, which can be quenched by EtOH to deliver racemic compounds **1** and **2**. Finally, the proposed biosynthesis of compounds **3** and **4** formed compound **5,** suggesting that the mild conditions used during the extraction process were favorable for selective mono ethylation. Hence, we conclude that these isolates could be artifacts, and these artifacts obtained under acidic co-metabolites act as a catalyst during the extraction/isolation of the insect *T. sinensis*.

### 2.2. Biological Activity

**Figure 4:**
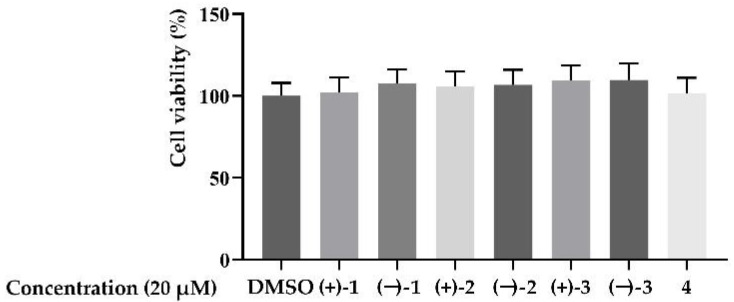
Assessment of the toxicity of compounds **1**–**4** in NRK-52e cells. NRK-52e cells were exposed to compounds at 20 μM for 48 h, and the cell viability was assessed by using the CCK-8 assay. *n* = 6 biologically independent cells.

**Figure 5:**
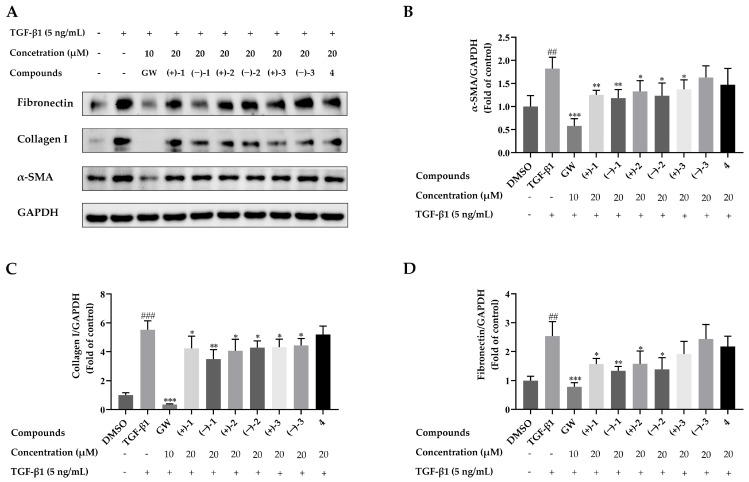
Compounds **1**–**4** attenuate kidney fibrosis in TGF-β1-induced NRK-52e cells. (**A**) The protein levels of α-SMA, collagen I, and fibronectin were determined by Western blot, and GAPDH was used as a control. (**B**–**D**) Quantification data of α-SMA (**B**), collagen I (**C**), and fibronectin (**D**). The results are expressed as mean ± SD. ^##^ *p* < 0.01, ^###^ *p* < 0.001 compared with DMSO alone. * *p* < 0.05, ** *p* < 0.01, *** *p* < 0.001 compared with TGF-β1 alone. *n* = 4 biologically independent cells. GW788388 (GW) as a positive control.

**Figure 6:**
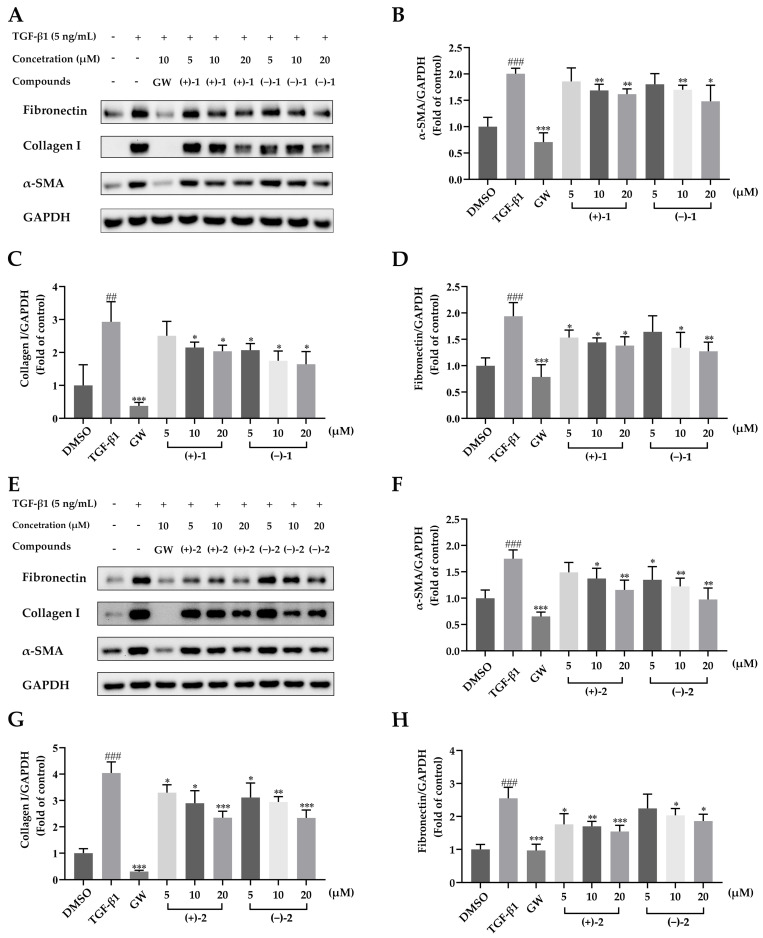
Compounds (±)-**1** and (±)-**2** attenuate renal fibrosis in TGF-β1-induced NRK-52e cells. Compound (±)-**1** dose-dependently inhibits renal fibrosis-related protein expression. Quantification of α-SMA (**B**), collagen I (**C**), and fibronectin (**D**) were tested by Western blot analyses (**A**) normalized by GAPDH. Compound (±)-**2** dose-dependently relieves renal fibrosis-related protein expression. Western blot analyses (**E**) and quantitative data of α-SMA (**F**), collagen I (**G**), and fibronectin (**H**). The results are expressed as mean ± SD. ^##^ *p* < 0.01, ^###^ *p* < 0.001 compared with DMSO alone. * *p* < 0.05, ** *p* < 0.01, *** *p* < 0.001 compared with TGF-β1 alone. *n* = 4 biologically independent cells. GW as a positive control.

Chronic kidney disease (CKD) is commonly associated with renal fibrosis and is regarded as its histological endpoint. Similar to fibrosis in other organs, renal fibrosis is characterized by the pathological deposition of the extracellular matrix (ECM) [29]. Multiple factors contribute to the development of fibrosis, and, among them, transforming growth factor-β1 (TGF-β1) plays a crucial role in driving this pathological process [30]. Therefore, the anti-renal fibrosis activities of the newfound compounds in NRK-52E cells induced by TGF-β1 were evaluated by measuring the levels of ECM proteins such as fibronectin, collagen I, and α-SMA. Initially, the cells’ potential cytotoxicity was successfully eliminated through testing cell viability by a CCK-8 assay; the compounds were nontoxic at 20 μM (Figure 4). Subsequently, we proceeded to evaluate the expression of ECM-related proteins, and found that the expression of α-SMA, collagen I, and fibronectin exhibited a notable reduction in response to compounds (±)-**1** and (±)-**2** (Figure 5). Furthermore, dose–response studies revealed that the anti-fibrotic activities of compounds (±)-**1** (Figure 6A–D) and (±)-**2** (Figure 6E–H) appeared to be more prominent with increasing concentrations; even the inhibition of ECM-associated proteins was discernible at 5 μM.

## 3. Materials and Methods

### 3.1. General

To obtain NMR spectra, a Bruker AV-500 or AV-600 spectrometer (Bruker, Karlsruhe, Germany) was utilized, with TMS serving as the internal standard. LC-MS chromatograms and HRESIMS came from a Shimazu LC-20AD AB SCIEX triple TOF X500R MS spectrometer (Shimadzu Corporation, Tokyo, Japan) with a C18 column (2.1 mm × 50 mm, i.d., 5 μm, Waters Corporation, Milford, MA, USA). Optical rotations were obtained from the Anton Paar MCP-100 digital polarimeter (Anton Paar, Graz, Austria). CD and UV spectra were obtained using a Jasco J-815 circular dichroism spectrometer (JASCO, Tokyo, Japan). For the column chromatography experiments, the following materials were utilized: Sephadex LH-20 (Amersham Pharmacia, Uppsala, Sweden), macroporous adsorbent resins (Rohm Haas AMBERLITET™ XAD™ 16N, USA), MCI gel CHP 20P (75–150 μm, Mitsubishi Chemical Industries, Tokyo, Japan), YMC gel ODS-A-HG (40–60 μm; YMC Co., Kyoto, Japan), and silica gel (200–300 mesh, Qingdao Marine Chemical Inc., Qingdao, China). The preparative and semi-preparative HPLC were used through a Saipuruisi (SEP) chromatograph (Separation (Beijing) Technology Co., Ltd., Beijing, China) with a YMC-Actus ODS-A column (250 mm × 20 mm, i.d., 5 µm) or a YMC-Pack ODS column (250 mm × 10 mm, i.d., 5 μm). Chiral separations were conducted using a chiral HPLC equipped with a UV detector and a Daicel Chiralpak IC column (250 mm × 4.6 mm, i.d., 5 μm).

### 3.2. Insect Material

The insects were identified as the egg cases of *T. sinensis* by Prof. Da-Rong Yang at the Kunming Institute of Zoology, Chinese Academy of Sciences, which were purchased from Anshan, Liaoning, China, in March 2021. The voucher specimen (CHYX0647) has been placed in the School of Pharmacy, Shenzhen University Medical School, Shenzhen University, China.

### 3.3. Extraction and Isolation

The insect materials (30.0 kg) were pulverized and then extracted three times with 50% ethanol (200 L × 48 h × 1, 150 L × 48 h × 2) at room temperature. This process resulted in the production of the crude extract, weighing 2.1 kg. Afterwards, the extract was fractionated using a macroporous resin column, and carefully eluted with EtOH/H_2_O (0–100%) to produce six fractions (Fr.A–Fr.F). The comprehensive isolated procedures are included in the Supporting Information. Racemic mixtures of compounds **1**–**3** were acquired and subsequently purified by chiral HPLC on a Daicel Chiralpak column (IC, 250 mm × 4.6 mm, i.d., 5 μm).

Mantidisamide E (**1**): yellow powder; UV (MeOH) *λ*_max_ (log*ε*) 221 (2.72), 238 (2.98), 264 (2.43), and 280 (2.49) nm; {[α]_D_^25^ −170.00 (*c* 0.03, MeOH); CD (MeOH) *Δε*_211_ +4.90, *Δε*_215_ +3.13, *Δε*_230_ +10.81, *Δε*_249_ −13.52, *Δε*_265_ −3.22; (−)-**1**}; {[α]_D_^25^ +270.00 (*c* 0.04, MeOH); CD (MeOH) *Δε*_205_ −7.43, *Δε*_213_ −5.05, *Δε*_230_ −16.01, *Δε*_248_ +18.88, *Δε*_265_ +4.46; (+)-**1**}; HRESIMS *m*/*z* 248.0353 [M + Na]^+^ (calcd for C_10_H_11_NO_3_SNa, 248.0352); ^1^H; and ^13^C NMR data (see Table 1).
Mantidisamide F (**2**): yellow powder; UV (MeOH) *λ*_max_ (log*ε*) 218 (2.87), 224 (2.85), 237 (2.95), and 263 (2.40) nm; {[α]_D_^25^ −180.00 (*c* 0.03, MeOH); CD (MeOH) *Δε*_217_ +13.45, *Δε*_248_ −14.95, *Δε*_261_ −3.61; (−)-**2**}; {[α]_D_^25^ +160.00 (*c* 0.03, MeOH); CD (MeOH) *Δε*_224_ −4.97, *Δε*_248_ +8.17, *Δε*_263_ +2.24; (+)-**2**}; HRESIMS *m*/*z* 309.0916 [M–H]^−^ (calcd for C_14_H_17_N_2_O_4_S, 309.0915); ^1^H; and ^13^C NMR data (see Table 1).
Mantidisamide G (**3**): colorless gum; UV (MeOH) *λ*_max_ (log*ε*) 205 (3.02), 221 (2.74), 232 (2.80), and 304 (2.21) nm; {[α]_D_^25^ −6.67 (*c* 0.03, MeOH); CD (MeOH) *Δε*_206_ −1.97, *Δε*_215_ −0.91, *Δε*_228_ −2.06; (−)-**3**}; {[α]_D_^25^ +3.33 (*c* 0.03, MeOH); CD (MeOH) *Δε*_208_ +1.94, *Δε*_222_ +0.59, *Δε*_233_ +1.17; (+)-**3**}; HRESIMS *m*/*z* 279.0808 [M–H]^−^ (calcd for C_13_H_15_N_2_O_3_S, 279.0809); ^1^H; and ^13^C NMR data (see Table 2).
Mantidisamide H (**4**): colorless gum; UV (MeOH) *λ*_max_ (log*ε*) 205 (3.21), 228 (2.87), 241 (2.92), and 261 (2.45) nm; HRESIMS *m*/*z* 279.0813 [M–H]^−^ (calcd for C_13_H_15_N_2_O_3_S, 279.0809); ^1^H; and ^13^C NMR data (see Table 2).

### 3.4. ECD Calculations

The ECD calculations were conducted using a similar approach as described in the literature [31]. The lowest energy of all the conformers of each compound was given by CONFLEX 7 software, and then the optimized and ECD calculations were carried out based on the mPW1PW91/6-311 + G(d,p) level with the PCM model in methanol for (8*R*)-**1** and the B3LYP/6-31G (d, p) level with PCM in methanol for (8*R*)-**2** and (7*R*)-**3**. SpecDis 1.62 and Origin 2019 programs were utilized to analyze the ultimate data.

### 3.5. Biological Evaluation

#### 3.5.1. Cell Culture

Normal rat kidney proximal tubular epithelial cells (NRK-52e) (Cell Bank of China Science Academy, Shanghai, China) were cultured in 10 cm dishes and maintained in high glucose DMEM (C11995500BT, Gibco, Waltham, MA, USA) supplemented with 10% fetal bovine serum (FBS) (2094468CP, Gibco, Waltham, MA, USA), 100 U/mL penicillin, and 100 μg/mL streptomycin. The cells were incubated at 37 °C in a humidified atmosphere with 5% CO_2_.

#### 3.5.2. Cell Viability Assay

NRK-52e cells (5 × 10^4^ cells/mL) were seeded into 96-well plates filled with complete DMEM (DMEM with 10% FBS, 100 U/mL penicillin, and 100 μg/mL streptomycin) and incubated for 24 h at 37 °C. Afterward, cells were exposed to DMSO or various compounds for 48 h. Subsequently, 100 μL of a combined solution comprising 10 μL of CCK-8 (CCK-8, Beyotime, Shanghai, China) solution and 90 μL of DMEM was introduced into each well, followed by an additional incubation period of 1 h at 37 °C. In the end, the absorbance for each well at 450 nm was determined using a microplate reader (BioTek, Winooski, VT, USA).

#### 3.5.3. Western Blot Analysis

NRK-52e cells were placed in 6-well plates with 2 mL per well (5 × 10^4^ cells/mL) and left to incubate for 12 h. After a 6 h starvation period, cells were exposed to different concentrations of compounds **1**–**4** or DMSO or GW788388 (HY-10326, MCE, USA) in DMEM containing 4% FBS for 48 h. Furthermore, recombinant TGF-β1 (5 ng/mL) (HY-P7118, MCE, Rahway, New Jersey, USA) was added to each plate to induce fibrosis in NRK-52e cells. Total proteins were extracted from cell lines using the radioimmunoprecipitation assay buffer (Beyotime, Shanghai, China) that included a protease cocktail (Roche, Penzberg, Germany). Subsequently, the protein samples were quantified using a BCA assay (Thermo Fisher Scientific, Waltham, MA, USA). In the next step, the cell proteins were then subjected to SDS-PAGE electrophoresis. Following electrophoresis, the proteins were transferred to a PVDF membrane (Merck Millipore, Darmstadt, Germany). They were then blocked using a mixture of 5% skimmed milk powder and TBST solution for 1 h. Subsequently, the primary antibodies were incubated overnight at 4 °C, followed by a secondary horseradish peroxidase-conjugated antibody for 1 h at room temperature. In this study, primary antibodies were used as follows: anti-fibronectin (dilution 1:1000; ab268020; Abcam, Cambridge, UK), anti-collagen1 (dilution 1:1000; ab270993; Abcam), anti-α-SMA (dilution 1:1000; A2547; Sigma, Kawasaki-shi, Japan), and anti-GAPDH (dilution 1:2000; sc-365062; Santa Cruz, Dallas, TX, USA). In addition, secondary horseradish peroxidase-conjugated antibody contained anti-rabbit IgG (dilution 1:5000; 7074; CST, Danvers, MA, USA) and anti-mouse IgG (dilution 1:5000; 7076; CST).

#### 3.5.4. Statistical Analysis

All data were analyzed using IBM SPSS Statistics 22, and the results are represented as mean ± SD. Graphpad Prism 8 (GraphPad Software, San Diego, CA, USA) was employed for performing statistical analyses. The normality of the data was tested using the Shapiro–Wilk normality test. Comparisons between groups were made using one-way ANOVA, followed by the Student–Newman–Kuels test. *p* < 0.05 was considered a significant difference. Detailed statistical tests were stated in figure legends for each experiment.

## 4. Conclusions

In conclusion, new ethylated derivatives of sulfur and nitrogen-containing artifacts (**1**–**4**), along with twenty known compounds (**5**–**24**), were derived from the egg cases of the insect *T. sinensis*. The biological evaluation of the novel compounds showed that (±)-**1** and (±)-**2** displayed notable inhibitory effects on the expression of fibronectin, collagen I, and α-SMA in TGF-β1-induced NRK-52E cells, indicating their potential usage in the treating of renal fibrosis. Although these new compounds should be artifacts, the undescribed compounds **1** and **2** have shown noteworthy potential as anti-fibrotic agents. Importantly, **1** and **2** retain their new structures even without the presence of the ethoxy group, which may be of interest to chemists and biologists for deeper investigation of their properties. The current study will enrich the structural and biological diversity of non-peptide small molecules in traditional insect-derived medicines.

## Figures and Tables

**Table 1:** ^1^H NMR (500 MHz) and ^13^C NMR (150 MHz) data of **1** and **2** (*δ* in ppm, *J* in Hz).

No.	1 ^a^	1 ^b^		2 ^a^		2 ^b^	
	*δ*_H_ (*J* in Hz)	*δ*_H_ (*J* in Hz)	*δ*_C_	*δ*_H_ (*J* in Hz)	*δ*_C_	*δ*_H_ (*J* in Hz)	*δ*_C_
1			155.0		134.8		136.6
2	6.69, d (2.6)	6.74, d (2.6)	115.6	6.56, d (1.7)	113.6	6.61, d (1.7)	114.4
3			118.7		145.3		146.7
4			130.3		122.9		124.1
5	6.83 d (8.6)	6.85, d (8.6)	119.4		116.4		118.2
6	6.62, dd (8.6, 2.6)	6.66, dd (8.6, 2.6)	115.5	6.61, d (1.7)	118.6	6.67, d (1.7)	120.2
7			163.6		160.3		163.3
8	5.12, s	5.02, s	79.5	5.15, s	77.6	5.05, s	79.5
9	Ha: 3.70, dq (9.8, 7.0)	Ha: 3.83, dq (9.5, 7.0)	65.1	Ha: 3.69, dq (9.8, 7.0)	63.4	Ha: 3.83, dq (9.5, 7.0)	65.1
	Hb: 3.48, dq (9.8, 7.0)	Hb: 3.52, dq (9.5, 7.0)		Hb: 3.48, dq (9.8, 7.0)		Hb: 3.53, dq (9.5, 7.0)	
10	1.04, t (7.0)	1.12, t (7.0)	14.9	1.04, t (7.0)	14.5	1.12, t (7.0)	14.9
11				2.54, m	34.6	2.67, m	35.9
12				3.18, m	40.0	3.35, m	41.9
13					169.0		173.3
14				1.77, s	22.6	1.91, s	22.5
1-OH	9.39, brs						
3-OH				9.60, brs			
7-*N*H	10.54, brs			10.09, brs			
12-*N*H				7.88, t (5.4)			

^a^ Recorded in DMSO-*d*6. ^b^ Recorded in methanol-*d*4.

**Table 2:** ^1^H NMR (500 MHz) and ^13^C NMR (150 MHz) data of **3** and **4** (*δ* in ppm, *J* in Hz, in methanol-*d*).

No.	3		4	
	*δ*_H_ (*J* in Hz)	*δ*_C_	*δ*_H_ (*J* in Hz)	*δ*_C_
1		141.3		111.9
2	6.92, d (1.4)	110.5	6.67, s	99.4
3		153.1 ^a^		138.0
4		143.8		151.9
5		137.0 ^a^		138.8
6	7.47, d (1.4)	111.8		157.2
7	4.47, dd (8.0, 4.7)	81.5	2.96, t (6.9)	31.2
8	Ha: 3.44, m	47.2	3.42, t (6.9)	39.6
	Hb: 3.34, m			
9		173.5 ^a^		173.3
10	1.93, s	15.6	1.85, s	22.6
11	9.04, s	154.4	8.84, s	151.4
12	3.44, m	65.7	4.11, q (7.0)	65.8
13	1.20, t (7.0)	22.5	1.45, t (7.0)	15.3

^a^ Chemical shift was obtained from HMBC spectrum.

## Data Availability

Data are contained within the article and Supplementary Materials.

## Supplementary Materials

The following supporting information can be downloaded at: https://www.mdpi.com/article/10.3390/molecules29153491/s1, Figure S1: structures of compounds **5**–**24**; Figures S2–S12: NMR and UV spectra and HRESIMS of **1**; Figures S13–S24: NMR and UV spectra and HRESIMS of **2**; Figures S25–S33: NMR and UV spectra and HRESIMS of **3**; Figures S34–S40: NMR and UV spectra and HRESIMS of **4**; Figure S41: mPW1PW91/6-311 + G(d,p) optimized lowest energy conformers for (8*R*)-**1**; Figure S42: B3LYP/6-31G(d,p) optimized lowest energy conformers for (8*R*)-**2**; Figure S43: B3LYP/6-31G(d,p) optimized lowest energy conformers for (7*R*)-**3**; Figures S44–S45: LC-MS chromatograms of the crude extract (extracted with acetone) and the compounds **1**–**4**; Figures S46–S47: LC-MS chromatograms of the crude extract (extracted with MeOH) and the compounds **1**–**4**; Table S1: the Cartesian coordinates of the lowest energy conformers for (8*R*)-**1**; Table S2: the Cartesian coordinates of the lowest energy conformers for (8*R*)-**2**; Table S3: the Cartesian coordinates of the lowest energy conformers for (7*R*)-**3**.
