# Supplementary material for: New Ethylated Derivatives of Sulfur- and Nitrogen-Containing Artifacts from Tenodera sinensis Egg Pod and Their Anti-Renal Fibrosis
Source: Molecules. 2024 Jul 25;29(15):3491. doi: 10.3390/molecules29153491 (PMC11314582; doi:10.3390/molecules29153491)
Supplement: Supplementary file 1 [file molecules-29-03491-s001.zip › molecules-3090742-supplementary.pdf]

Supplementary Information

New ethylated derivatives of sulfur and nitrogen-containing artifacts from *Tenodera sinensis* egg pod and their anti-renal fibrosis

Ye-Fei Chen [1, 2 †], Shi-Gang Peng [1, 3 †], Yong-Ming Yan[1] and Yong-Xian Cheng [1,2,3*]

[1] Guangdong Provincial Key Laboratory of Chinese Medicine Ingredients and Gut Microbiomics, Institute for Inheritance-Based Innovation of Chinese Medicine, Marshall Laboratory of Biomedical Engineering, School of Pharmacy, Shenzhen University Medical School, Shenzhen University, Shenzhen 518055, PR China

[2] Guangdong Key Laboratory for Biomedical Measurements and Ultrasound Imaging, National-Regional Key Technology Engineering Laboratory for Medical Ultrasound, School of Biomedical Engineering, Shenzhen University Medical school, Shenzhen 518060, PR China

[3] School of Pharmacy, Chengdu University of Traditional Chinese Medicine, Chengdu 611137, PR China

[*] Correspondence: yxcheng@szu.edu.cn (Y.-X.C.); Tel./Fax: +86-0755-26902073

[†] These authors contributed equally to this paper.

Content

Detailed Isolation Procedures
Figure S1. Structures of compounds **5**–**24**.
Figure S2. ^{1}H NMR spectrum of **1** in DMSO-d_6.
Figure S3. ^{1}H NMR spectrum of **1** in methanol-d_4.
Figure S4. ^{13}C NMR and DEPT spectra of **1** in methanol-d_4.
Figure S5. ^{1}H-^{1}H COSY spectrum of **1** in methanol-d_4.
Figure S6. HSQC spectrum of **1** in methanol-d_4.
Figure S7. HMBC spectrum of **1** in methanol-d_4.
Figure S8. ROESY spectrum of **1** in DMSO-d_6.
Figure S9. HRESIMS of **1**.
Figure S10. CD and UV spectra of (−)-**1** in methanol.
Figure S11. CD and UV spectra of (+)-**1** in methanol.
Figure S12. The A) Chiral HPLC chromatogram of **1**;
B) Chiral HPLC chromatogram of (−)-**1**;
C) Chiral HPLC chromatogram of (+)-**1**.
Analysis condition: Daicel Chiralpak IC column (250 mm × 4.6 mm, i.d., 5 μm), n-hexane/EtOH, 81:19, 0.05% TFA, flow rate: 1 mL/min.
Figure S13. ^{1}H NMR spectrum of **2** in DMSO-d_6.
Figure S14. ^{13}C NMR and DEPT spectra of **2** in DMSO-d_6
Figure S15. ^{1}H NMR spectrum of **2** in methanol-d_4.
Figure S16. ^{13}C NMR spectrum of **2** in methanol-d_4.
Figure S17. ^{1}H-^{1}H COSY spectrum of **2** in methanol-d_4.
Figure S18. HSQC spectrum of **2** in methanol-d_4.
Figure S19. HMBC spectrum of **2** in methanol-d_4.
Figure S20. ROESY spectrum of **2** in DMSO-d_6.
Figure S21. HRESIMS of **2**.
Figure S22. CD and UV spectra of (+)-**2** in methanol.
Figure S23. CD and UV spectra of (−)-**2** in methanol.
Figure S24. The A) Chiral HPLC chromatogram of **2**;
B) Chiral HPLC chromatogram of (+)-**2**;
C) Chiral HPLC chromatogram of (−)-**2**.
Analysis condition: Daicel Chiralpak IC column (250 mm × 4.6 mm, i.d., 5 μm), n-hexane/EtOH, 93:7, 0.05% TFA, flow rate: 1 mL/min.
Figure S25. ^{1}H NMR spectrum of **3** in methanol-d_4.
Figure S26. ^{13}C NMR and DEPT spectra of **3** in methanol-d_4.
Figure S27. ^{1}H-^{1}H COSY spectrum of **3** in methanol-d_4.
Figure S28. HSQC spectrum of **3** in methanol-d_4.
Figure S29. HMBC spectrum of **3** in methanol-d_4.
Figure S30. HRESIMS of **3**.
Figure S31. CD and UV spectra of (−)-**3** in methanol.
Figure S32. CD and UV spectra of (+)-**3** in methanol.
Figure S33. The A) Chiral HPLC chromatogram of **3**;

B) Chiral HPLC chromatogram of (−)-**3**;

C) Chiral HPLC chromatogram of (+)-**3**.

Analysis condition: Daicel Chiralpak IC column (250 mm × 4.6 mm, i.d., 5 μm), n-hexane/EtOH, 81:19, 0.05% TFA, flow rate: 1 mL/min.

Figure S34.^{1}H NMR spectrum of **4** in methanol-d_4.

Figure S35. ^{13}C NMR and DEPT spectra of **4** in methanol-d_4.

Figure S36. ^{1}H-^{1}H COSY spectrum of **4** in methanol-d_4.

Figure S37. HSQC spectrum of **4** in methanol-d_4.

Figure S38. HMBC spectrum of **4** in methanol-d_4.

Figure S39. UV spectrum of **4** in methanol.

Figure S40. HRESIMS of **4**.

Figure S41. mPW1PW91/6-311+G(d,p) optimized lowest energy conformers for (8*R)*-**1**.

Figure S42. B3LYP/6-31G(d,p) optimized lowest energy conformers for (8*R)*-**2**.

Figure S43. B3LYP/6-31G(d,p) optimized lowest energy conformers for (7*R)*-**3**.

Figure S44. LC-MS chromatogram of the crude extract and the compounds (**1** and **2**). To check whether compounds **1** and **2** are natural products or artifacts, Mantidis Ootheca was extracted with acetone.

Figure S45. LC-MS chromatogram of the crude extract and the compounds (**3** and **4**). To check whether compounds **3** and **4** are natural products or artifacts, Mantidis Ootheca was extracted with acetone.

Figure S46. LC-MS chromatogram of the crude extract and the compounds (**1** and **2**). To check whether compounds **1** and **2** are natural products or artifacts, Mantidis Ootheca was extracted with MeOH.

Figure S47. LC-MS chromatogram of the crude extract and the compounds (**3** and **4**). To check whether compounds **3** and **4** are natural products or artifacts, Mantidis Ootheca was extracted with MeOH.

Table S1 The Cartesian coordinates of the lowest energy conformers for (8*R*)-**1**.

Table S2 The Cartesian coordinates of the lowest energy conformers for (8*R*)-**2**.

Table S3 The Cartesian coordinates of the lowest energy conformers for (7*R*)-**3**

Detailed Isolation Procedures

The insect materials (30.0 kg), were pulverized and then extracted three times with 50% ethanol (200 L × 48 h × 1, 150 L × 48 h × 2) at room temperature. This process resulted in the production of the crude extract, weighing 2.1 kg. Afterwards, the extract was fractionated using a macro-porous resin (Rohm Haas AMBERLITET™ XAD™ 16N, USA) column, and carefully eluted with EtOH/H_2O (0%–100%) to produce six fractions (Fr.A–Fr.F). Fr.D (57.1 g) was fractionated into seven parts (Fr.D.A–Fr.D.G) by using a MCI gel CHP 20P column (MeOH/H_2O, 10%–100%).

Fr.D.C. (400.0 mg) was gel filtered through Sephadex LH-20 (MeOH) to give three parts (Fr.D.C.1–Fr.D.C.3). The third part (98.2 mg) was fractionated into six parts (Fr.D.C.3.1–Fr.D.C.3.6) by using a semi-preparative HPLC (MeCN/H_2O, 11%, 0.05% HCOOH). Compound **6** (1.53 mg, t_R = 13.2 min; flow rate: 3 mL/min) was obtained from Fr.D.C.3.5 (4.2 mg) by using a semi-preparative HPLC (MeOH/H_2O, 34%, 0.05% HCOOH).

Fr.D.D（737.4 mg）was subjected to Sephadex LH-20 (MeOH) to give five parts (Fr.D.D.1–Fr.D.D.5). Fr.D.D.5 (83.4 mg) was cut by a semi-preparative HPLC (MeCN/H_2O, 18%, 0.05% HCOOH) to afford eight parts (Fr.D.D.5.1–Fr.D.D.5.8). Among them, Fr.D.D.5.7 is compound **7** (5.7 mg, t_R = 23.7 min; flow rate: 3 mL/min), and Fr.D.D.5.8 is **24** (5.7 mg, t_R = 24.6 min; flow rate: 3 mL/min). Fr.D.D.5.3 (8.1 mg) was purified by a semi-preparative HPLC (MeOH/H_2O, 14%, 0.05% HCOOH) to afford **19** (1.3 mg, t_R = 24.8 min; flow rate: 3 mL/min)

Fr.D.E (5.5 g) was passed through Sephadex LH-20 (MeOH) to provide five fractions (Fr.D.E.1–Fr.D.E.5). Fr.D.E.2 (1.2 g) was cut by an ODS column (MeOH/H_2O, 15%–100%) to give eight parts (Fr.D.E.2.1–Fr.D.E.2.8). Fr.D.E.2.2 (103.8 mg) was segregated by using preparative HPLC (MeCN/H_2O, 5%−100%, 0.05% HCOOH) to afford six parts (Fr.D.E.2.2.1–Fr.D.E.2.2.6). Fr.D.E.2.2.6 (5.9 mg) was purified by semi-preparative HPLC (MeCN/H_2O, 19%) to afford **22** (1.8 mg, t_R = 20.2 min; flow rate: 3 mL/min). Fr.D.E.3 (2.1 g) was cut by an ODS column (MeOH/H_2O, 30%–100%) to give five parts (Fr.D.E.3.1–Fr.D.E.3.5). **16** (2.4 mg, t_R = 19.9 min; flow rate: 3 mL/min) was purified from Fr.D.E.3.2 (51.7 mg) by semi-preparative HPLC (MeCN/H_2O, 15%, 0.05% HCOOH). Fr.D.E.3.3 (696.4 mg) was filtered through Sephadex LH-20 (MeOH) to produce four parts

(Fr.D.E.3.3.1–Fr.D.E.3.3.4). Of which, Fr.D.E.3.3.2 (497.3 mg) was cut into eight parts (Fr.D.E.3.3.2.1– Fr.D.E.3.3.2.8) by preparative HPLC (MeOH/H_2O, 10%−100%, 0.05% HCOOH). **5** (7.6 mg, t_R = 7.3 min; flow rate: 3 mL/min) was purified from Fr.D.E.3.3.2.2 (102 mg) by semi-preparative HPLC (MeCN/H_2O, 27%, 0.05% HCOOH). **21** (2.5 mg, t_R = 22.7 min; flow rate: 3 mL/min) and **13** (2.2 mg, t_R = 24.8 min; flow rate: 3 mL/min) was purified from Fr.D.E.3.3.2.3 (69.6 mg) by semi-preparative HPLC (MeCN/H_2O, 17%, 0.05% HCOOH). **15** (6.7 mg, t_R = 24.5 min; flow rate: 3 mL/min) was purified from Fr.D.E.3.3.2.4 (111.2 mg) by semi-preparative HPLC (MeCN/H_2O, 19%, 0.05% HCOOH). And **20** (3.8mg, t_R = 31.4min; flow rate: 3 mL/min) was purified from Fr.D.E.3.3.2.8 (6.0 mg) by semi-preparative HPLC (MeCN/H_2O, 17%, 0.05% HCOOH). Fr.D.E.4 (361.4 mg) was passed through preparative HPLC (MeCN/H_2O, 10%–100%, 0.05% HCOOH) to provide six fractions (Fr.D.E.4.1–Fr.D.E.4.6). Of which, Fr.D.E.4.3 (24.4 mg) was subjected to semi-preparative HPLC (MeCN/H_2O, 21%, 0.05% HCOOH) to afford **18** (6.5 mg, t_R = 20.9 min). Fr.D.E.4.4 (48.4 mg) was purified by semi-preparative HPLC (MeCN/H_2O, 19%, 0.05% HCOOH) to obtain **8 (**3.6 mg, t_R = 19.5 min; flow rate: 3 mL/min) and **1** (3.5 mg, t_R = 36.1 min). Fr.D.E.4.5 (56.3 mg) was subjected to semi-preparative HPLC (MeCN/H_2O, 23%, 0.05% HCOOH) to provide three fractions (Fr.D.E.4.5.1–Fr.D.E.4.5.3). Among them, Fr.D.E.4.5.2 is **23** (19.9 mg, t_R = 14.0 min; flow rate: 3 mL/min). Compound **9** (3.8 mg, t_R = 23.9 min; flow rate: 3 mL/min) was purified from Fr.D.E.4.6 (29.1 mg) by semi-preparative HPLC (MeCN/H_2O, 20%, 0.05% HCOOH).

Fr.D.F (8.6 g) was sub-fractionated into five parts (Fr.D.F.1–Fr.D.F.5) through Sephadex LH-20 (MeOH). Among them, Fr.D.F.2 (3.1 g) was subjected to an ODS column (MeOH/H_2O, 20%–100%) to give five parts (Fr.D.F.2.1–Fr.D.F.2.5). Fr.D.F.2.5 (668.2 mg) was passed through preparative HPLC (MeCN/H_2O, 5%–100%, 0.05% HCOOH) to provide six fractions (Fr.D.F.2.5.1–Fr.D.F.2.5.6). **Compound 3** (2.0 mg, t_R = 19 min; flow rate: 3 mL/min) was purified from Fr.D.F.2.5.1 (85.4 mg) by semi-preparative HPLC (MeCN/H_2O, 18%, 0.05% HCOOH). Fr.D.F.3 (1.2 g) was subjected to a silica gel column eluting with CH_2Cl_2/Me_2CO (20:1–0:1) to afford nine portions (Fr.D.F.3.1–Fr.D.F.3.9). Fr.D.F.3.5 (121.4 mg) was passed through Sephadex LH-20 (MeOH) to obtain five portions (Fr.D.F.3.5.1–Fr.D.F.3.5.5). **4** (1.7 mg, t_R = 15.2 min; flow rate: 3 mL/min) was purified from Fr.D.F.3.5.3 (31.0 mg) by semi-preparative HPLC (MeCN/H_2O, 25%, 0.05% HCOOH). Fr.D.F.3.5.4 (31.0 mg)

was subjected to semi-preparative HPLC (MeCN/H_2O, 26%, 0.05% HCOOH) to afford **17** (2.3 mg, t_R = 15.2 min; flow rate: 3 mL/min) and **14** (3.9 mg, t_R = 19.4 min). Fr.D.F.3.6 (87.5 mg) was filtered through Sephadex LH-20 (MeOH) to afford three parts (Fr.D.F.3.6.1–Fr.D.F.3.6.3). Fr.D.F.3.6.2 (15.8 mg) was submitted to semi-preparative HPLC (MeCN/H_2O, 23%, 0.05% HCOOH) to obtain **2** (2.4 mg, t_R = 18.6 min). Compound **10** (8.1 mg, t_R = 12.7 min; flow rate: 3 mL/min) was purified from Fr.D.F.5 (102.1 mg) by semi-preparative HPLC (MeOH/H_2O, 51%, 0.05% HCOOH).

Fr.D.G (6.4 g) was cut to give three parts (Fr.D.G.1–Fr.D.G.3) through Sephedax LH-20 (MeOH). Fr.D.G.2 (1.9 g) was submitted to a silica gel column eluting with CH_2Cl_2/MeOH (40:1–0:1) to afford four portions (Fr.D.G.2.1–Fr.D.G.2.4). Fr.D.G.2.4 (572.3 mg) was divided into four parts (Fr.D.G.2.4.1–Fr.D.G.2.4.4) through Sephedax LH-20 (MeOH). Fr.D.G.2.4.4 (196.2 mg) was purified by a semi-preparative HPLC (MeCN/H_2O, 25%, 0.05% HCOOH) to afford compound **11** (21.6 mg, t_R = 39.6 min; flow rate: 3 mL/min). Fr.D.G.3 (750.0 mg) was cut by preparative HPLC (MeOH/H_2O, 20%–100%, 0.05% HCOOH) to give thirteen parts (Fr.D.E.3.1–Fr.D.E.3.13). **12** (2.1 mg, t_R = 19.5 min; flow rate: 3 mL/min) was purified from Fr.D.G.3.12 (20.2 mg) by semi-preparative HPLC (MeCN/H_2O, 29%, 0.05% HCOOH).

Afterwards, compounds **1**, **2**, and **3** are racemates, and were subjected to chiral HPLC (flow rate: 1 mL/min). These separations afforded (−)-**1** (1.7 mg, t_R = 9.7 min) and (+)-**1** (1.3 mg, t_R = 14.3 min) (n-hexane/ethanol containing 0.05% TFA in ethanol, 81:19) (Figure S12); (+)-**2** (1.4 mg, t_R = 36.5 min) and (−)-**2** (1.1 mg, t_R = 41.2 min) (n-hexane/ethanol containing 0.05% TFA in ethanol, 93:7) (Figure S24); (−)-**3** (0.8 mg, t_R = 10.4 min) and (+)-**3** (1.3 mg, t_R = 18.5 min) (n-hexane/ethanol containing 0.05% TFA in ethanol, 81:19) (Figure S33).

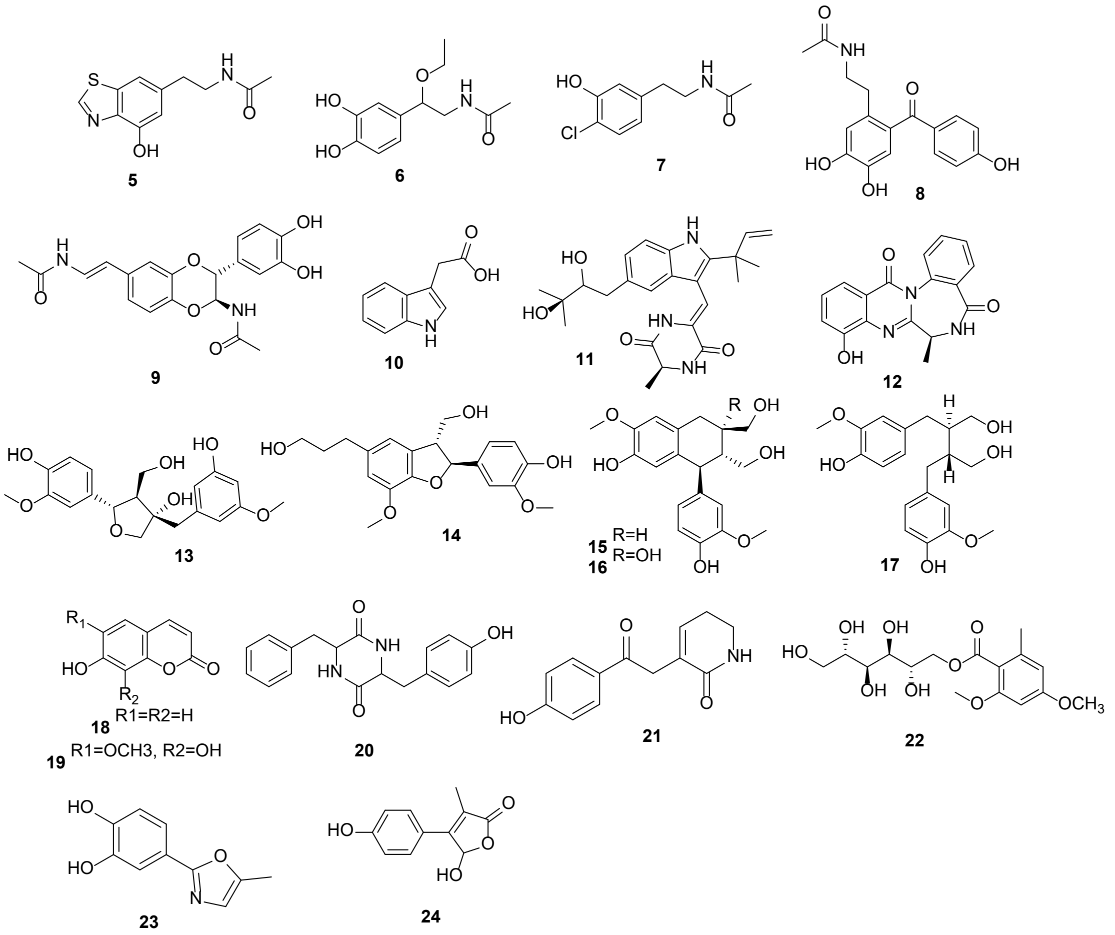

Figure S1. Structures of compounds **5−24**.

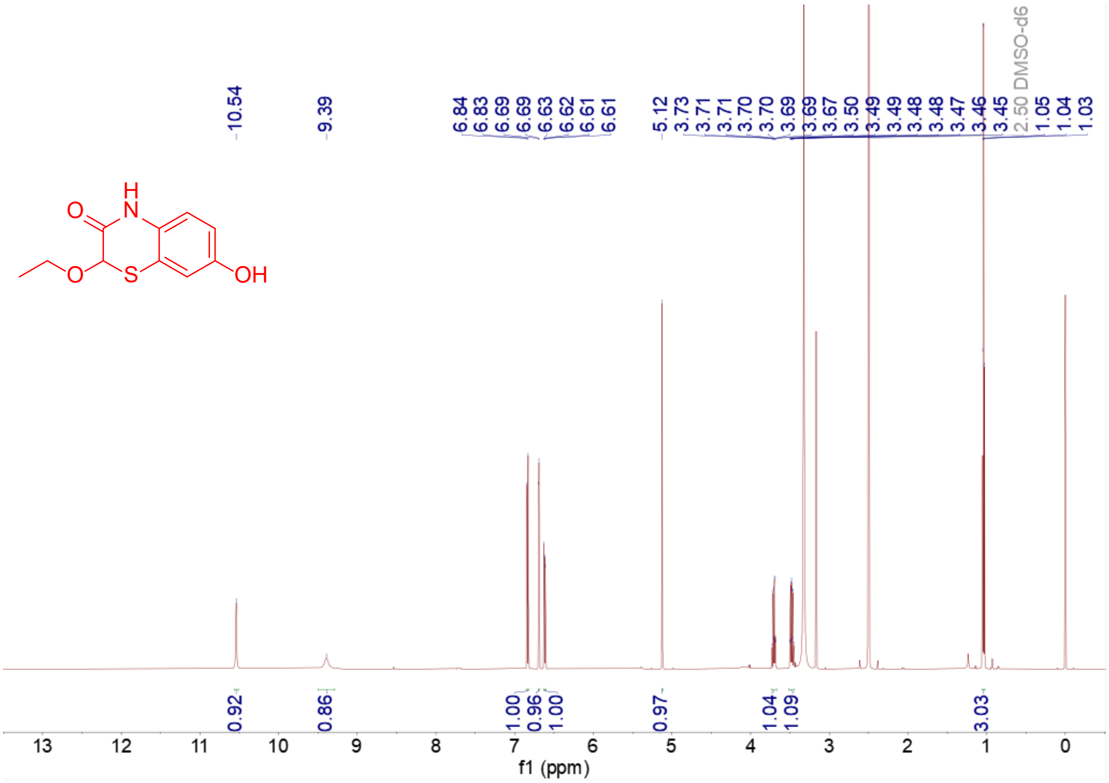

Figure S2. ^{1}H NMR (500 MHz) spectrum of **1** in DMSO-d_6.

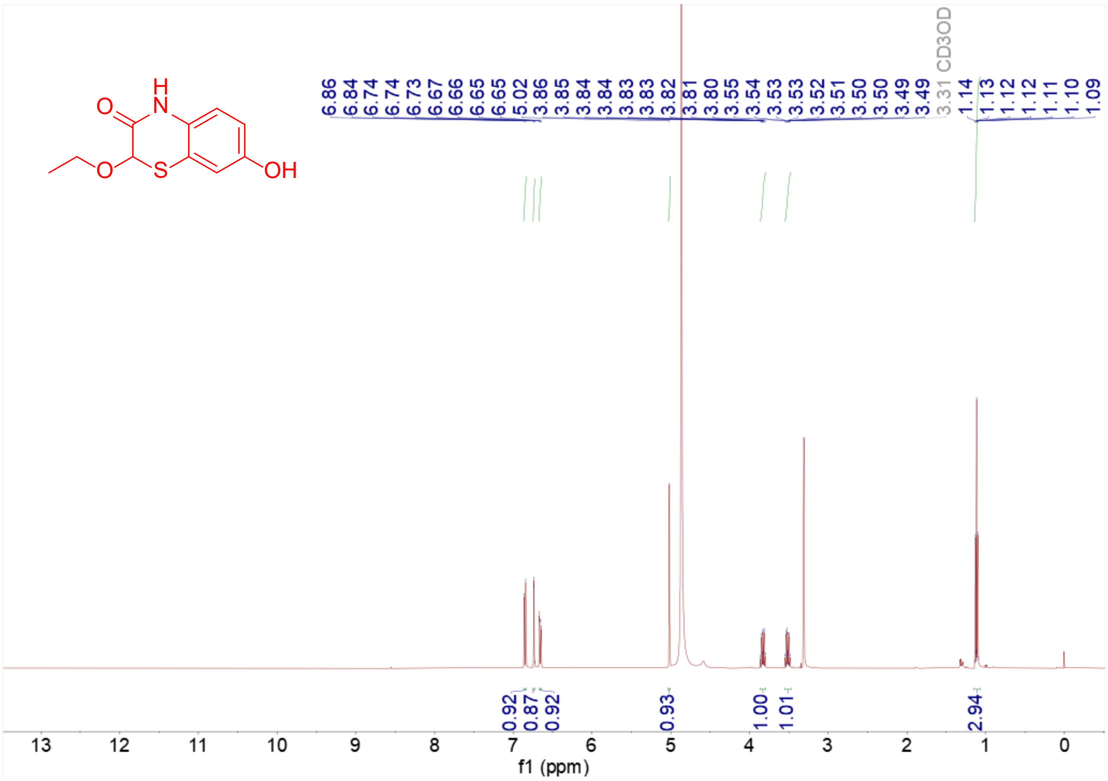

Figure S3. ^{1}H NMR (500 MHz) spectrum of **1** in methanol-d_4.

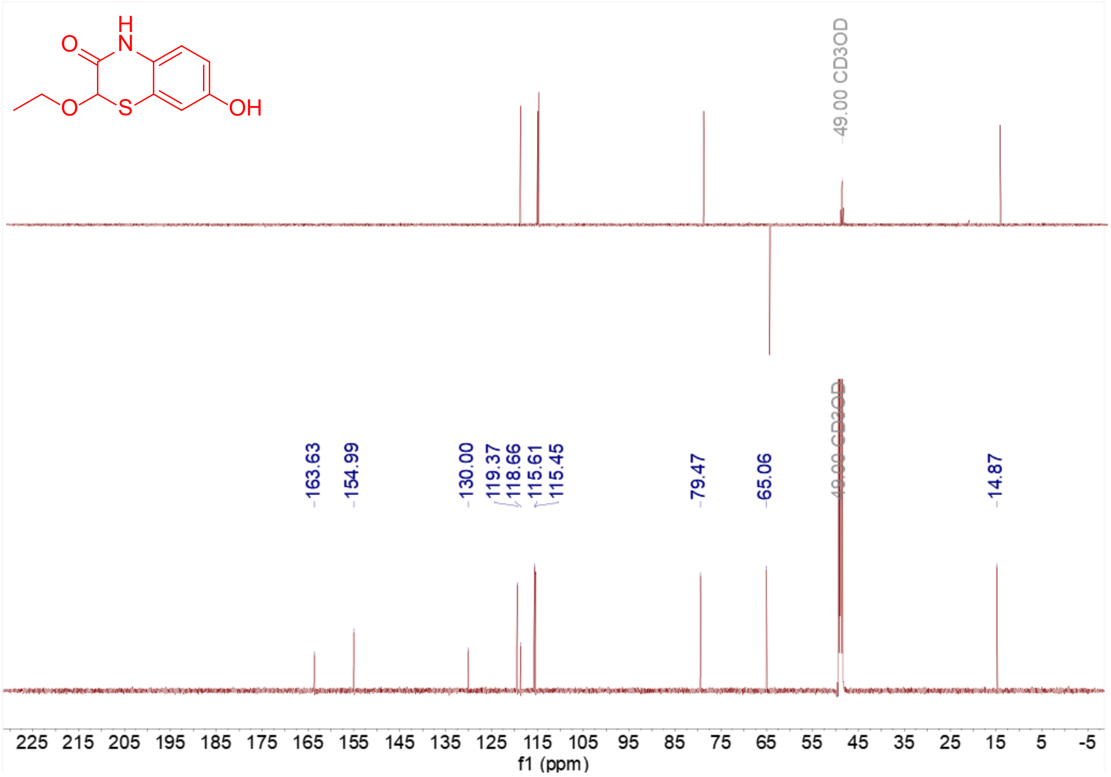

Figure S4. ^{13}C NMR and DEPT-135 (150 MHz) spectra of **1** in methanol-d_4.

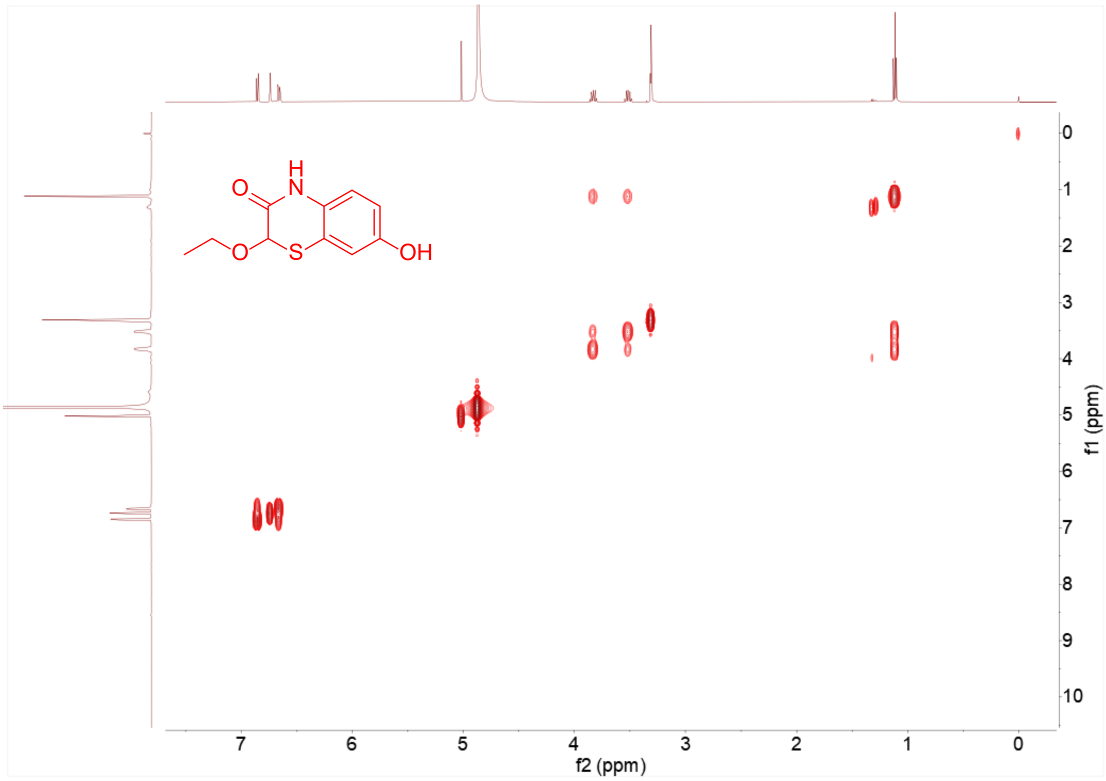

Figure S5. ^{1}H-^{1}H COSY (600 MHz) spectrum of **1** in methanol-d_4.

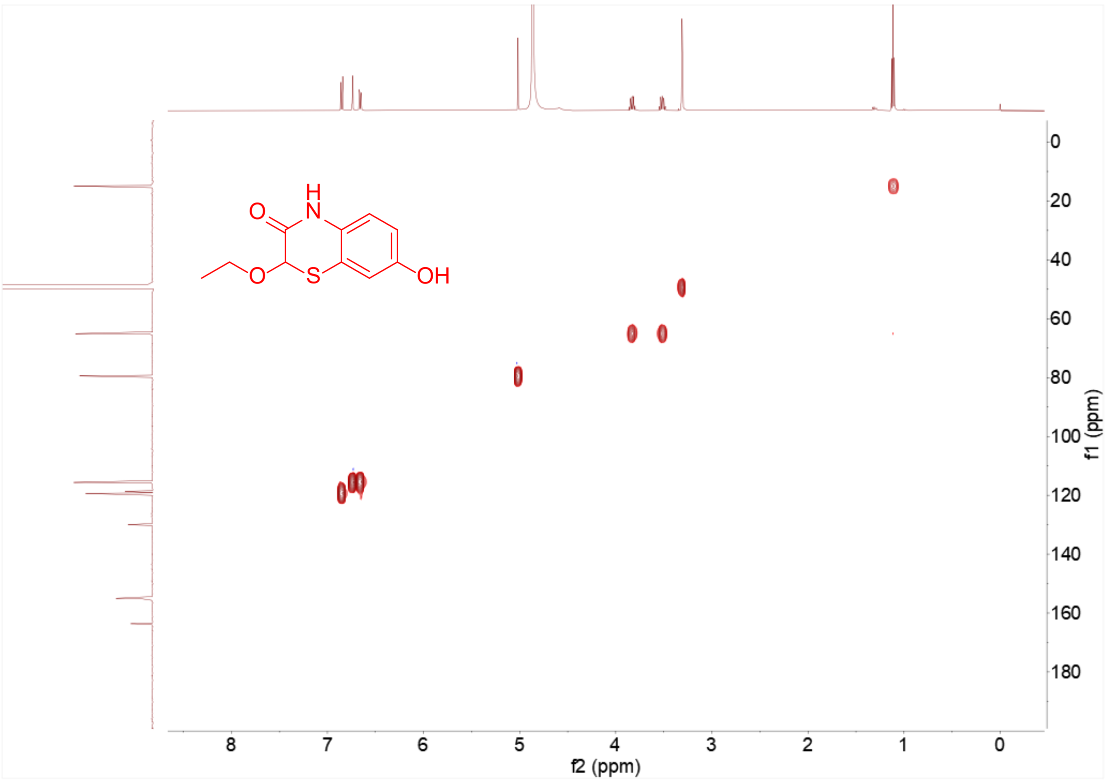

Figure S6. HSQC (600 MHz) spectrum of **1** in methanol-*d*4.

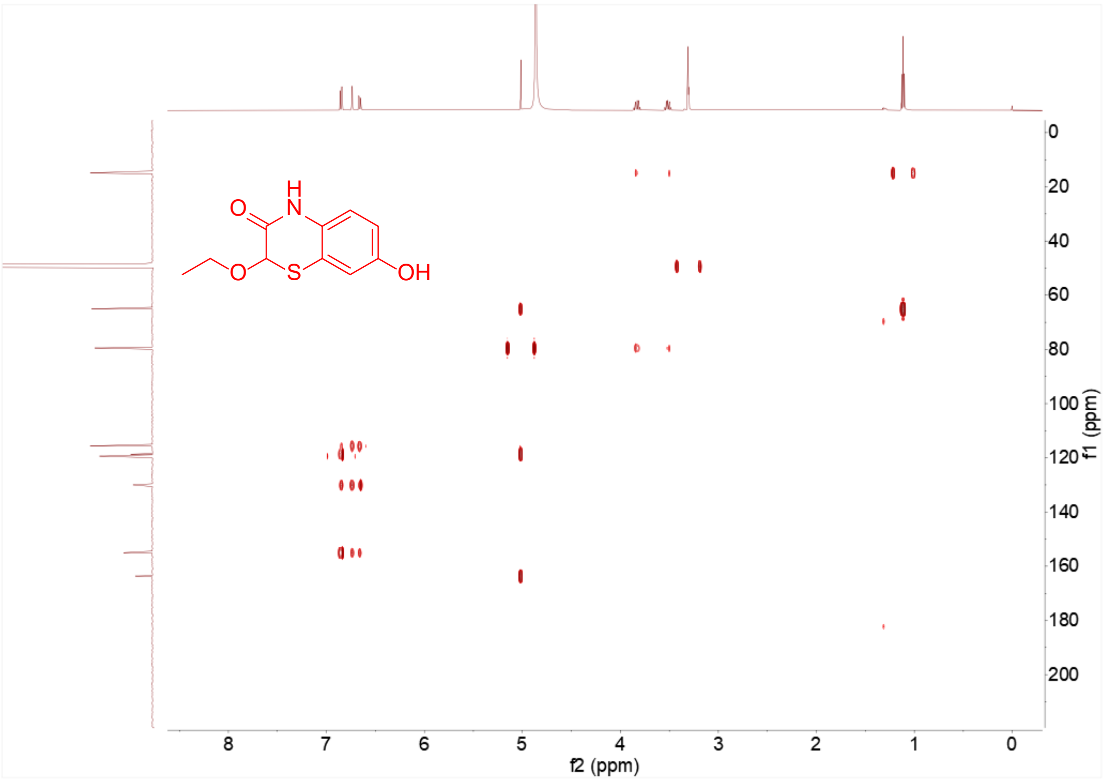

Figure S7. HMBC (600 MHz) spectrum of **1** in methanol-*d*4.

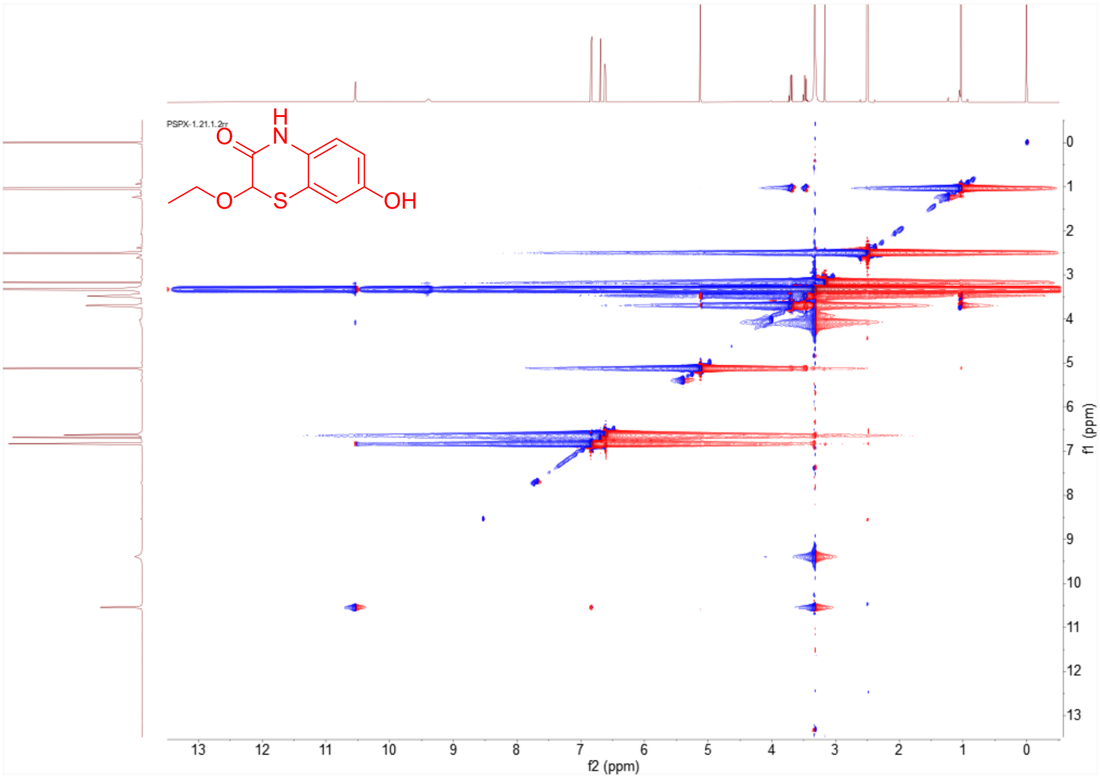

Figure S8. ROESY (600 MHz) spectrum of **1** in DMSO-d_6.

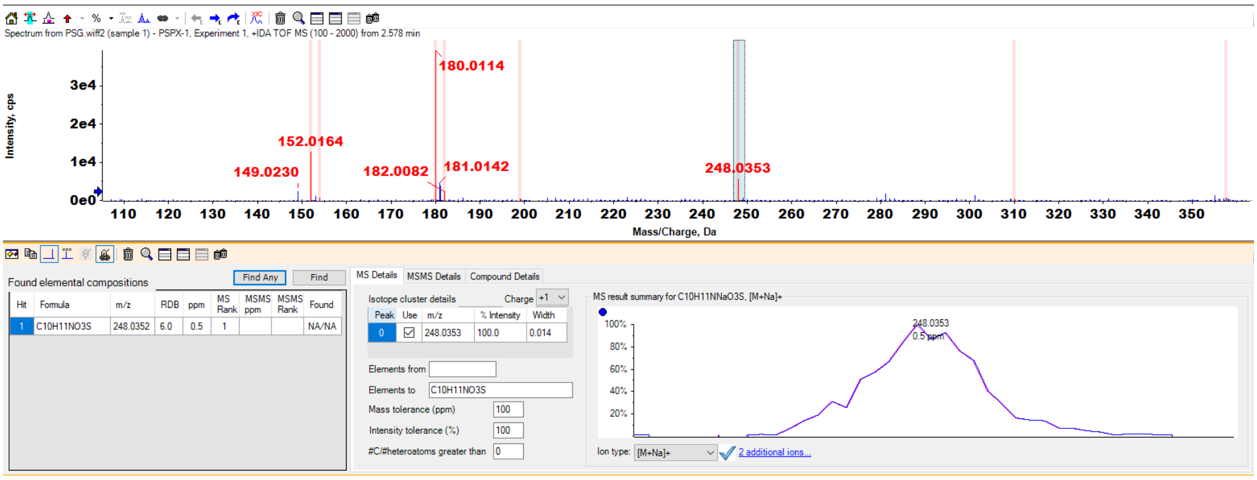

Figure S9. HRESIMS of **1**.

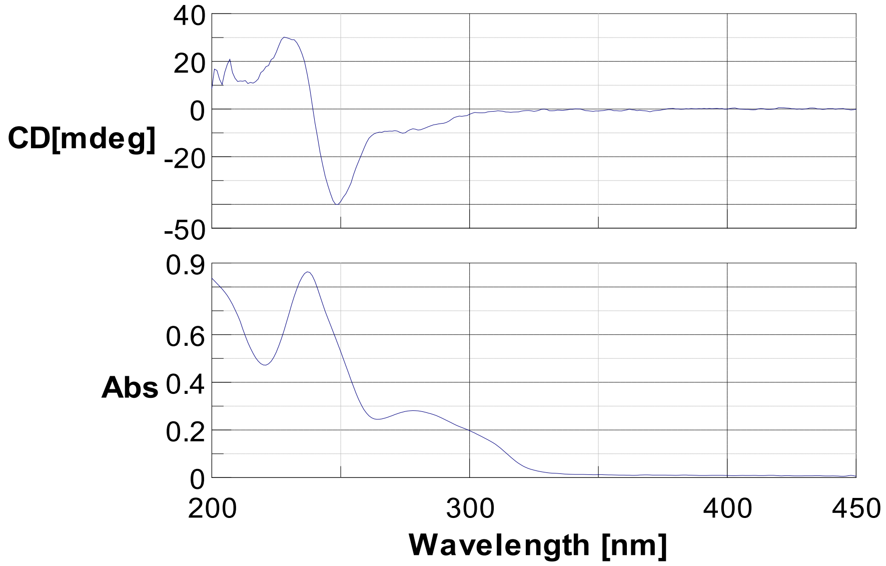

Figure S10. CD and UV spectra of (−)-**1**.

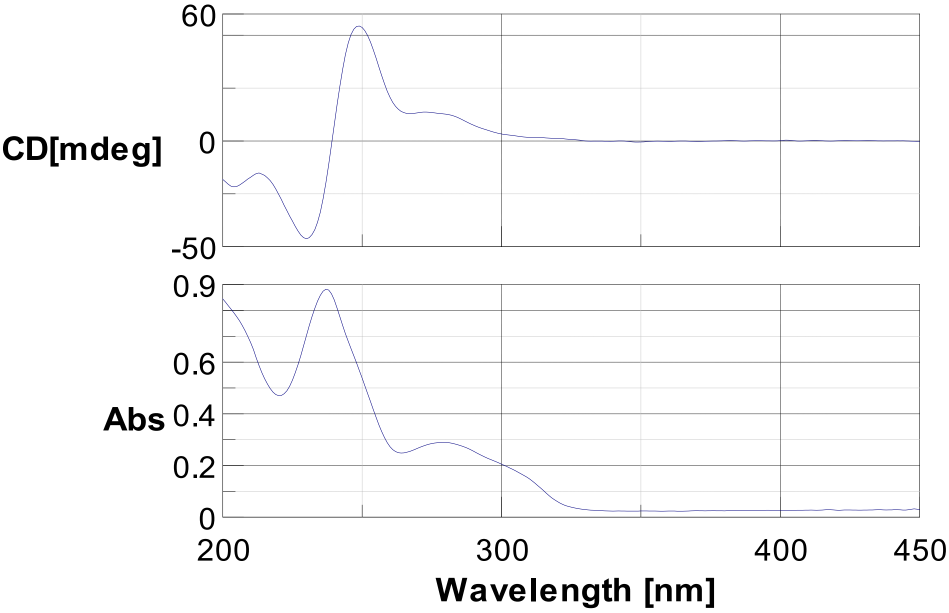

Figure S11. CD and UV spectra of (+)-**1**.

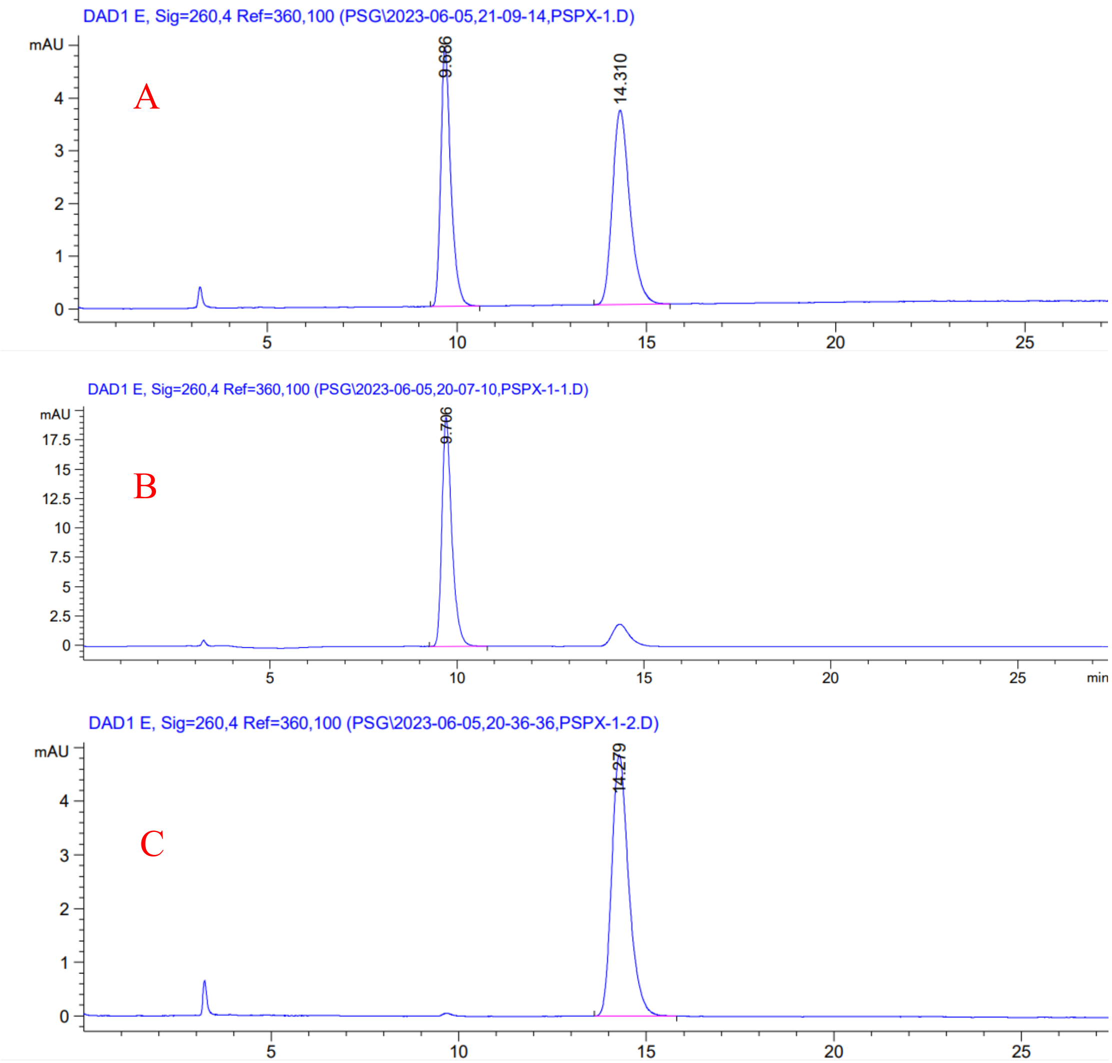

Figure S12. (A) Chiral HPLC chromatogram of **1**;
(B) Chiral HPLC chromatogram of (−)-**1**;
(C) Chiral HPLC chromatogram of (+)-**1**.

Analysis condition: Daicel Chiralpak IC column (250 mm × 4.6 mm, i.d., 5 μm), n-hexane/EtOH, 81:19, 0.05% TFA, flow rate: 1 mL/min.

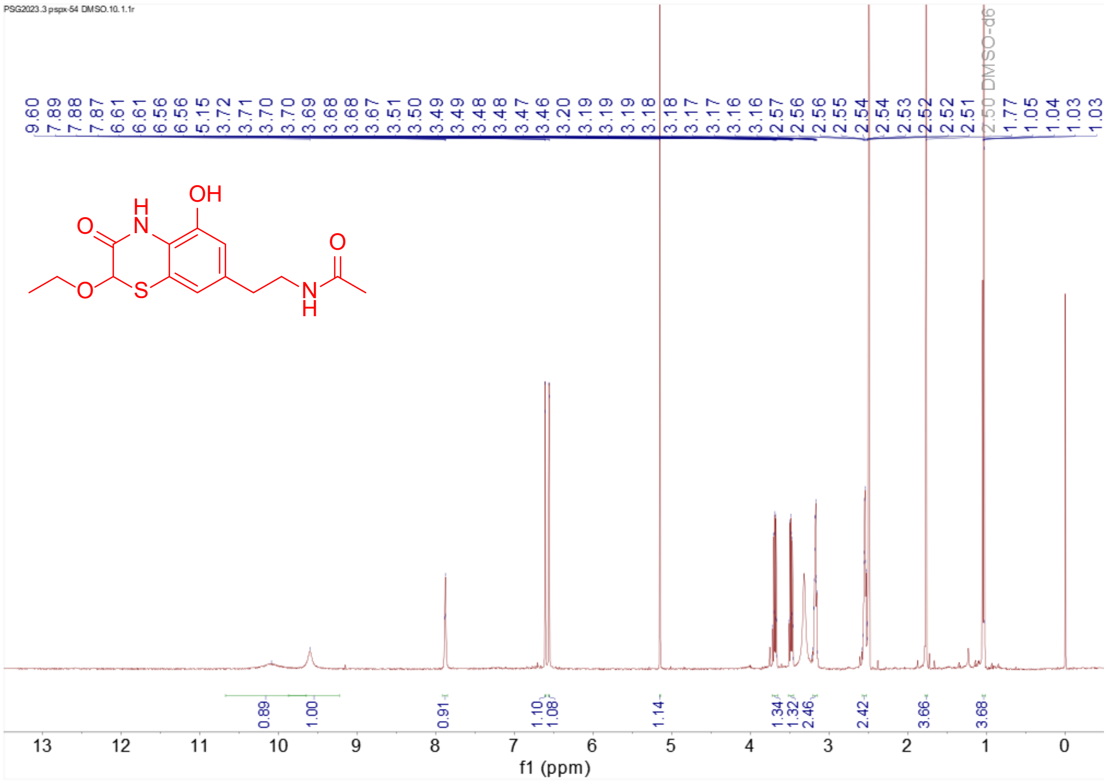

Figure S13. ^{1}H NMR (500 MHz) spectrum of **2** in DMSO-d_6.

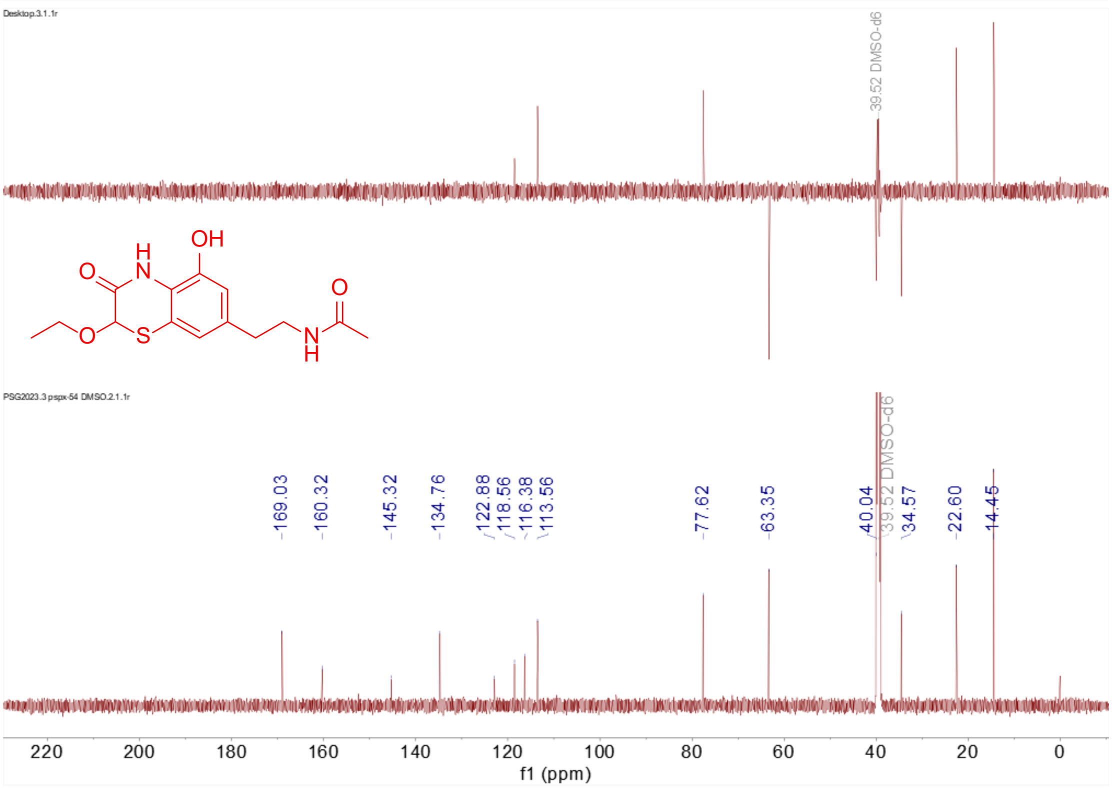

Figure S14. ^{13}C NMR and DEPT-135 (150 MHz) spectra of **2** in DMSO-d_6.

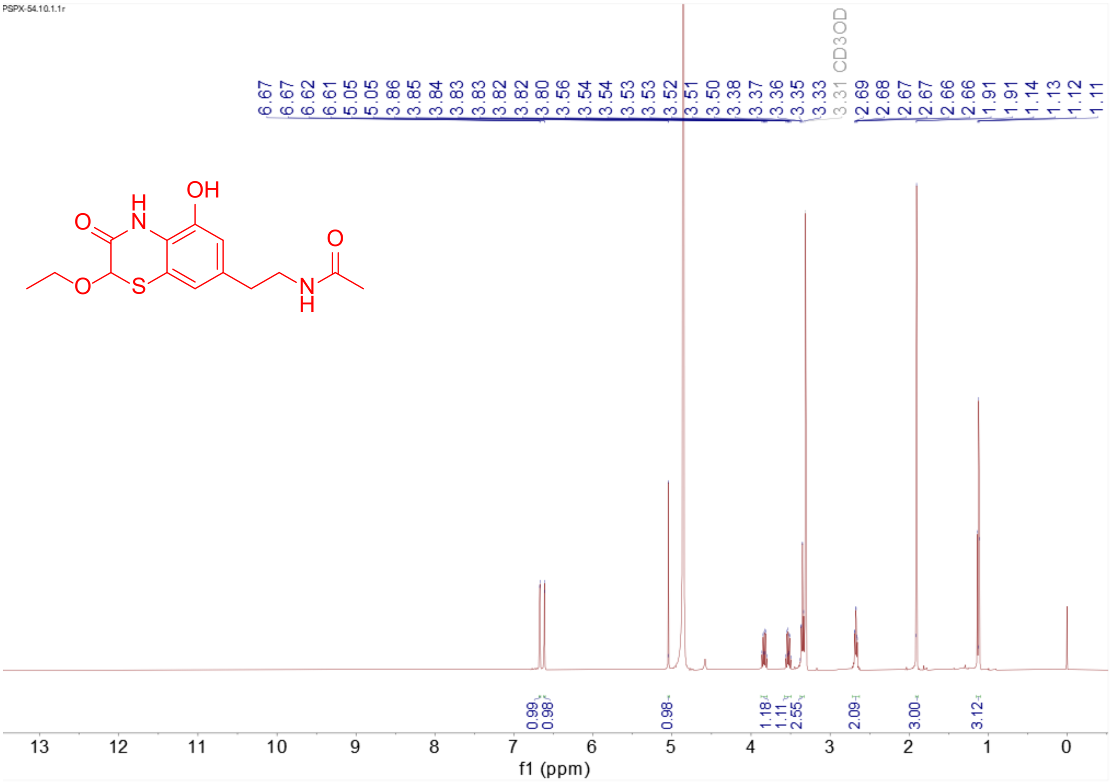

Figure S15. ^{1}H NMR (500 MHz) spectrum of **2** in methanol-d_4.

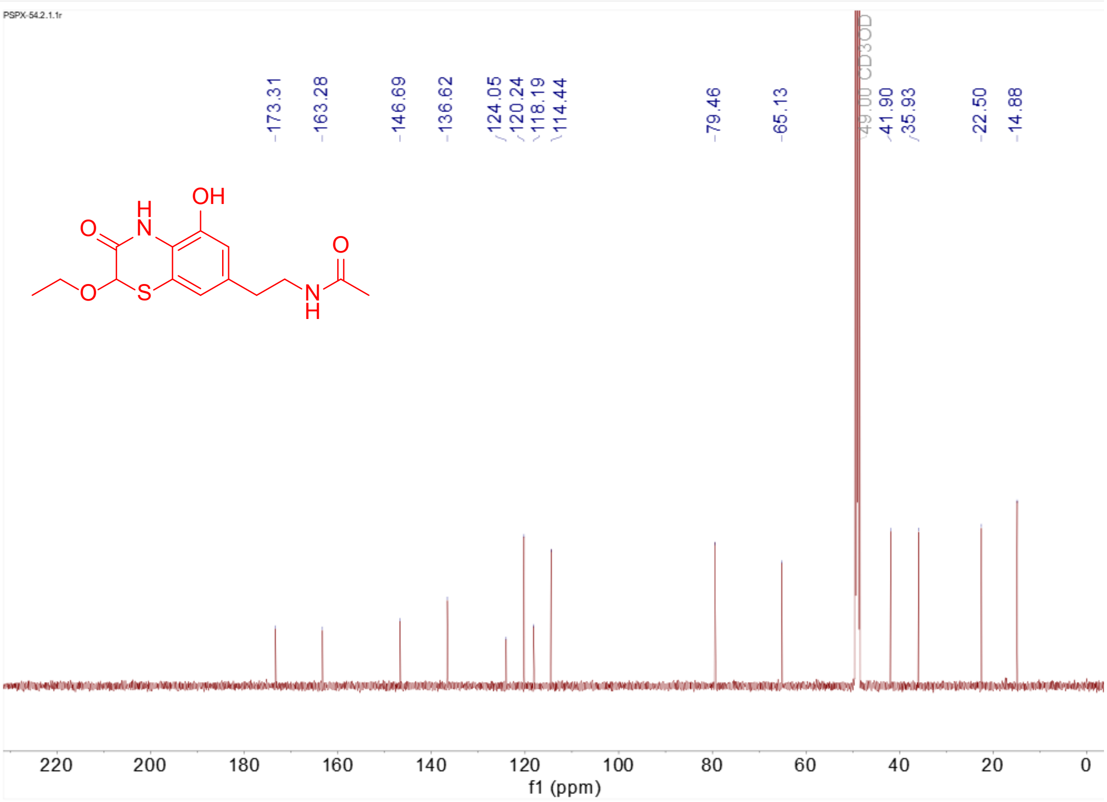

Figure S16. ^{13}C NMR (150 MHz) spectrum of **2** in methanol-d_4.

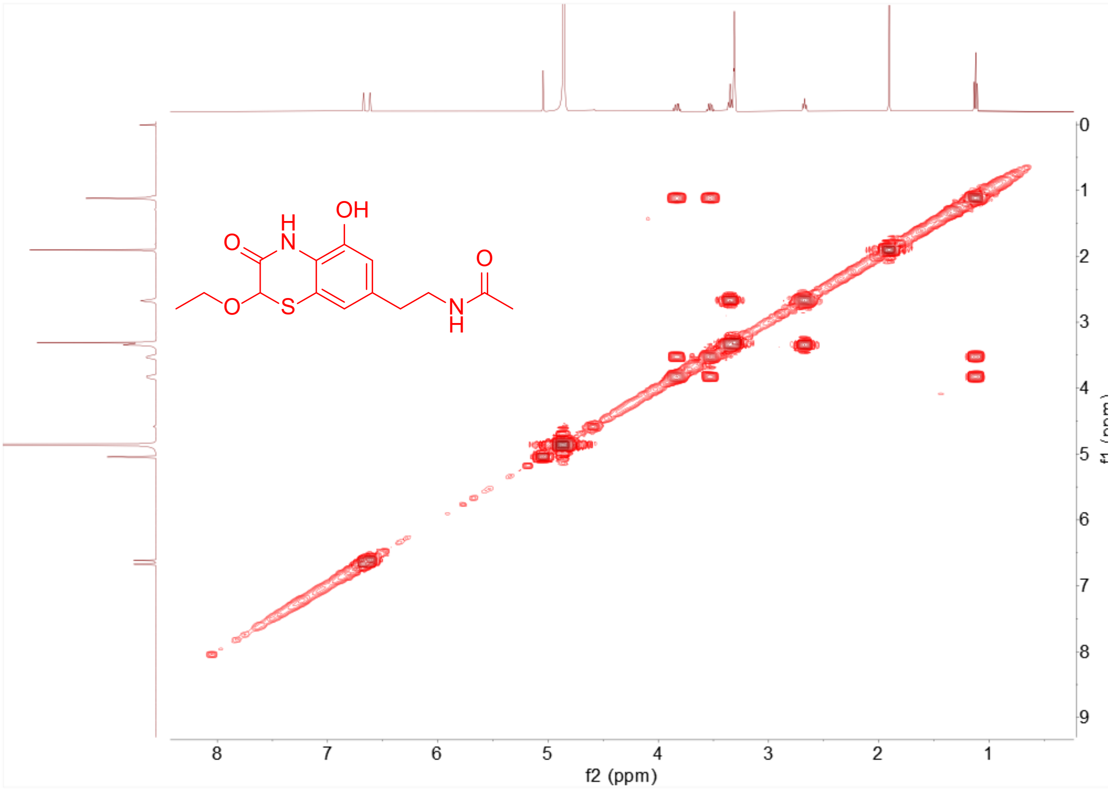

Figure S17. ^{1}H-^{1}H COSY (600 MHz) spectrum of **2** in methanol-*d*$_4$.

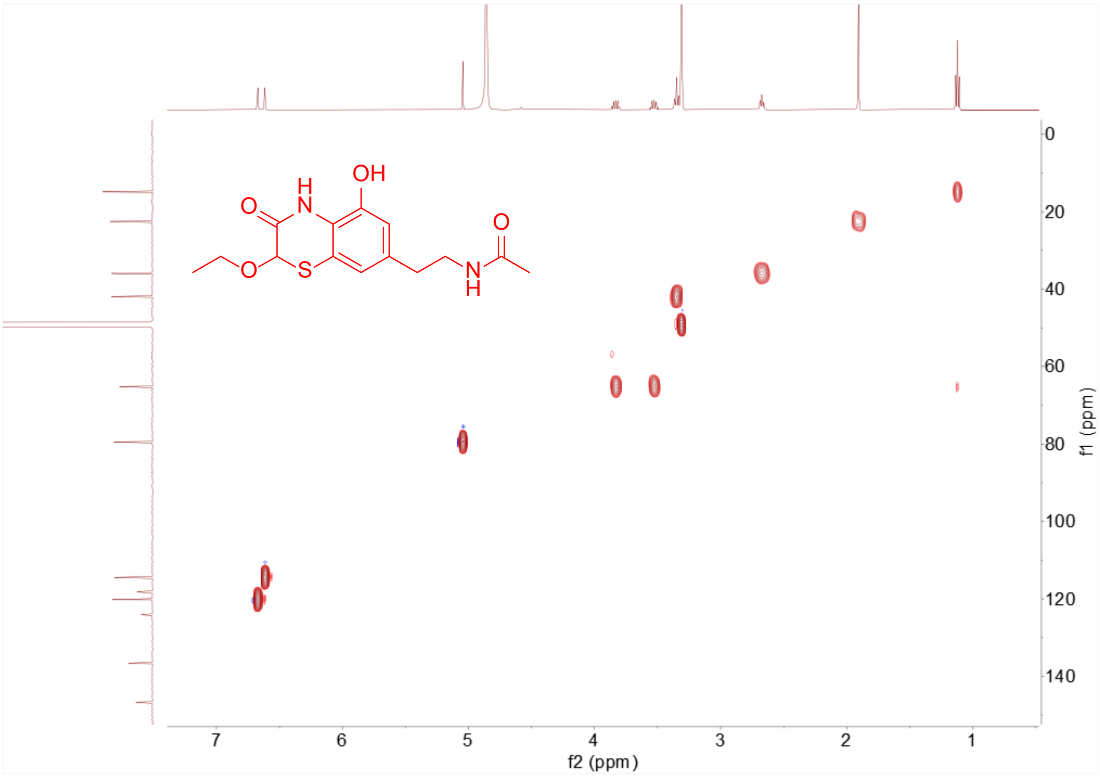

Figure S18. HSQC (600 MHz) spectrum of **2** in methanol-*d*$_4$.

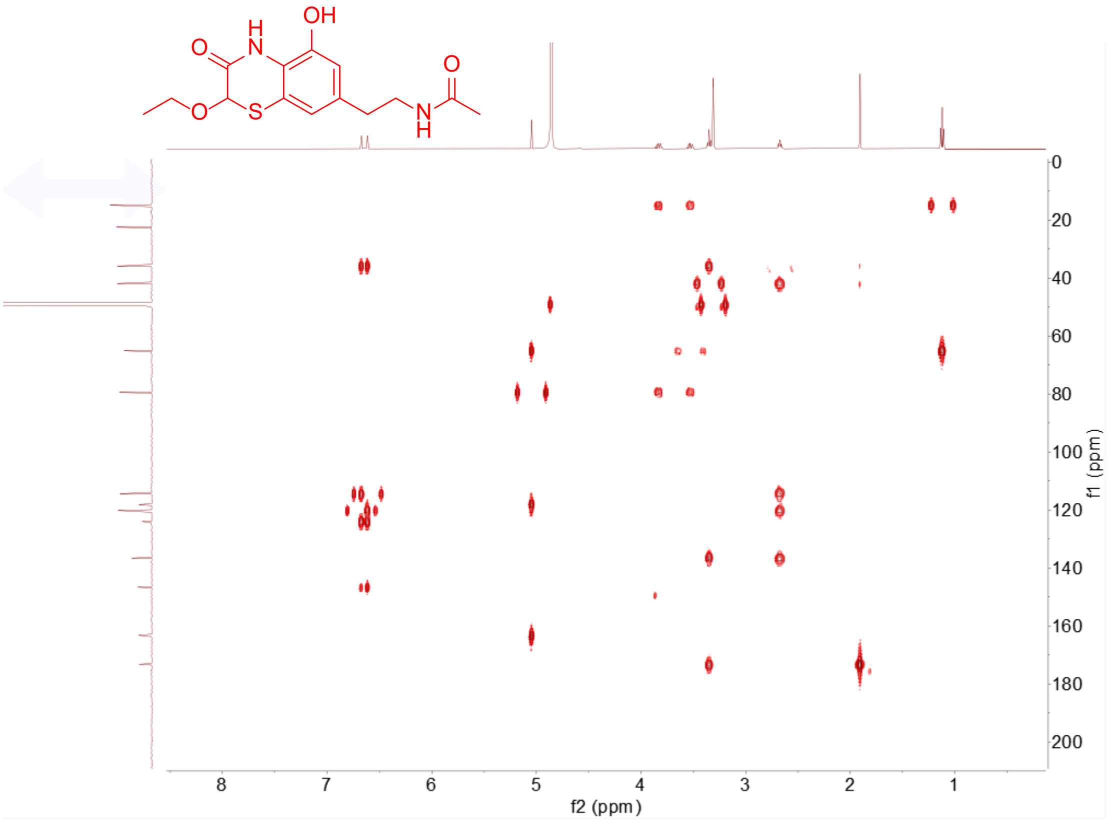

Figure S19. HMBC (600 MHz) spectrum of **2** in methanol-d_4.

.

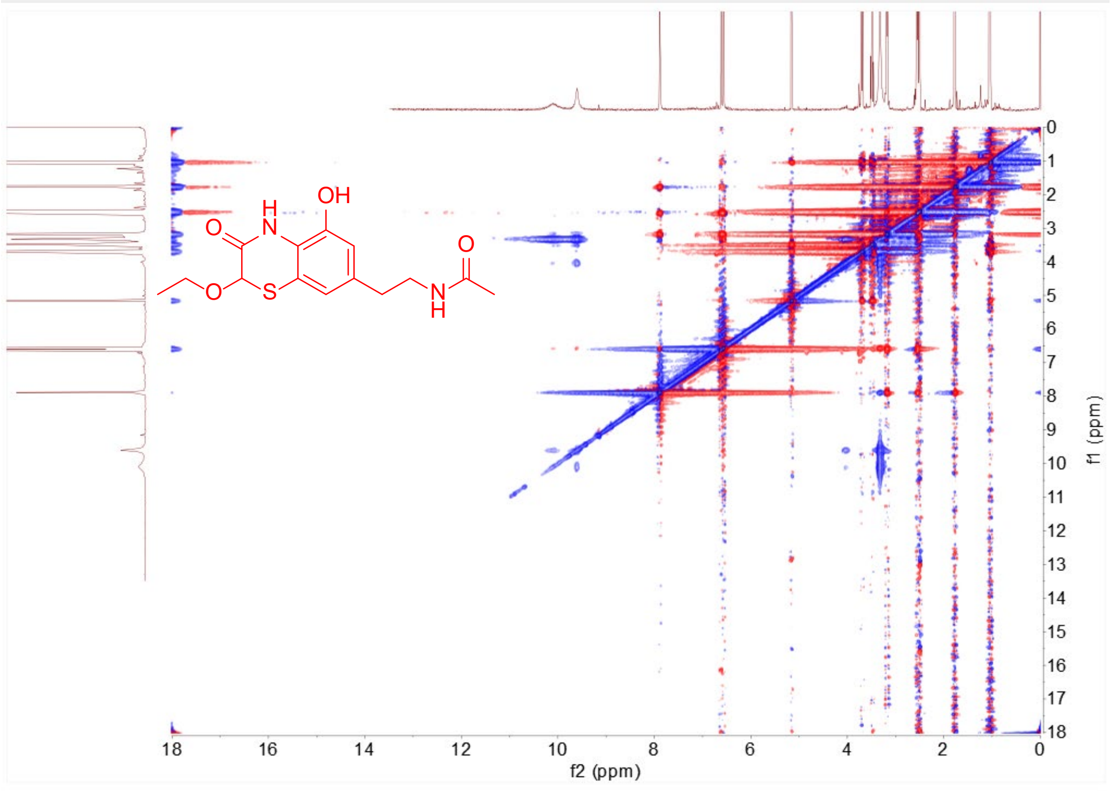

Figure S20. ROESY (600 MHz) spectrum of **2** in DMSO-d_6.

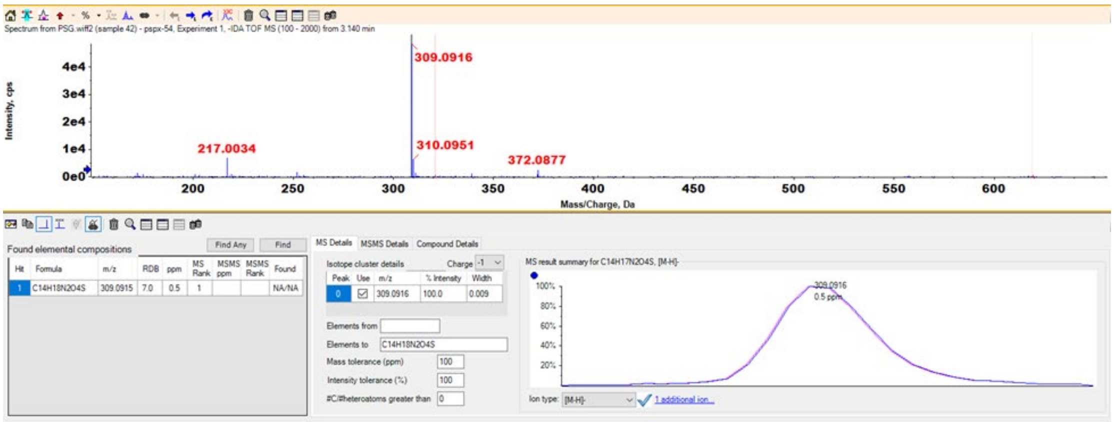

Figure S21. HRESIMS of **2**.

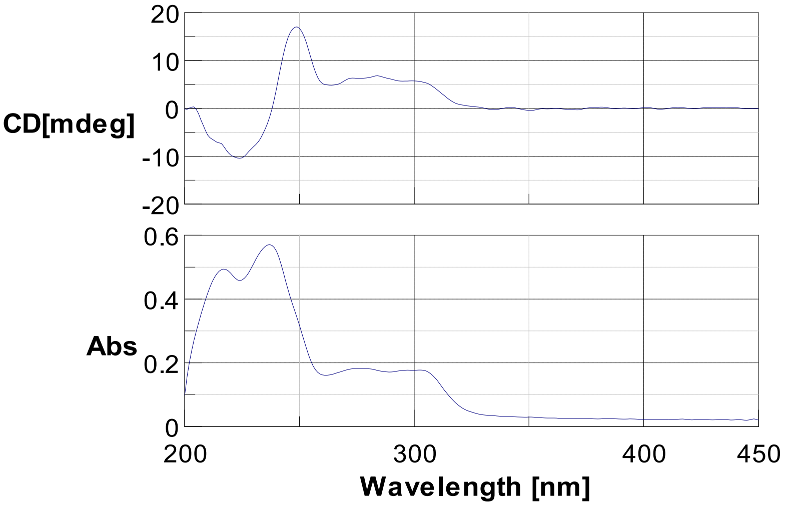

Figure S22. CD and UV spectra of (+)-**2** in methanol.

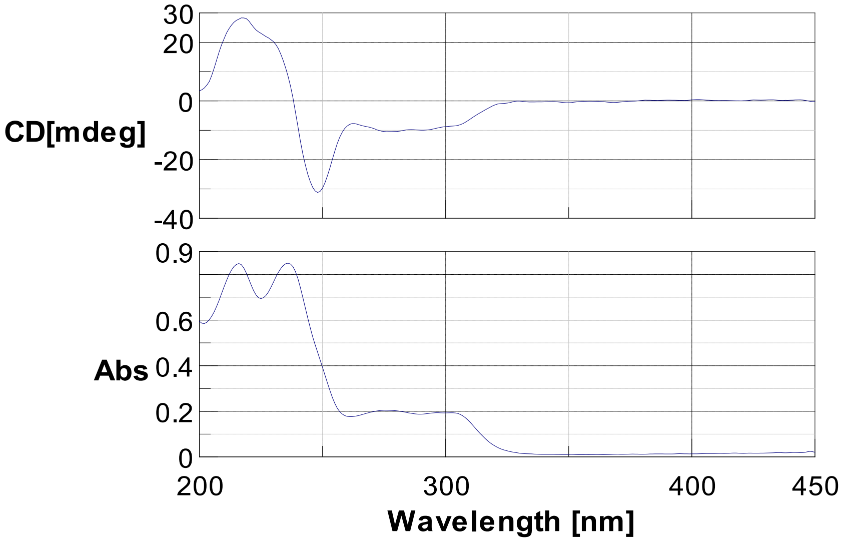

Figure S23. CD and UV spectra of (−)-**2** in methanol.

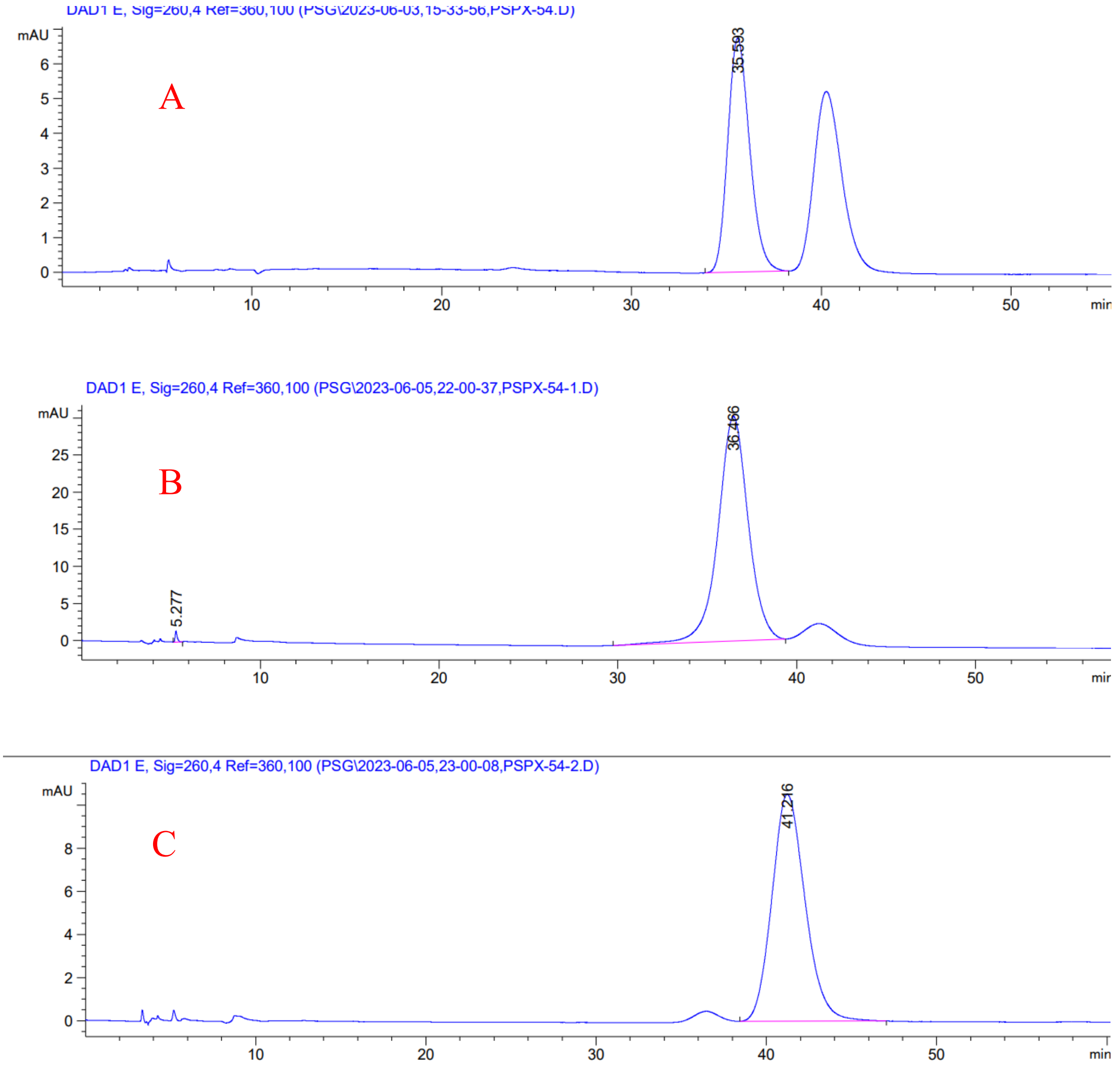

Figure S24. (A) Chiral HPLC chromatogram of **2**;
(B) Chiral HPLC chromatogram of (+)-**2**;
(C) Chiral HPLC chromatogram of (−)-**2**.

Analysis condition: Daicel Chiralpak IC column (250 mm × 4.6 mm, i.d., 5 μm), n-hexane/EtOH, 93:7, 0.05% TFA, flow rate: 1 mL/min.

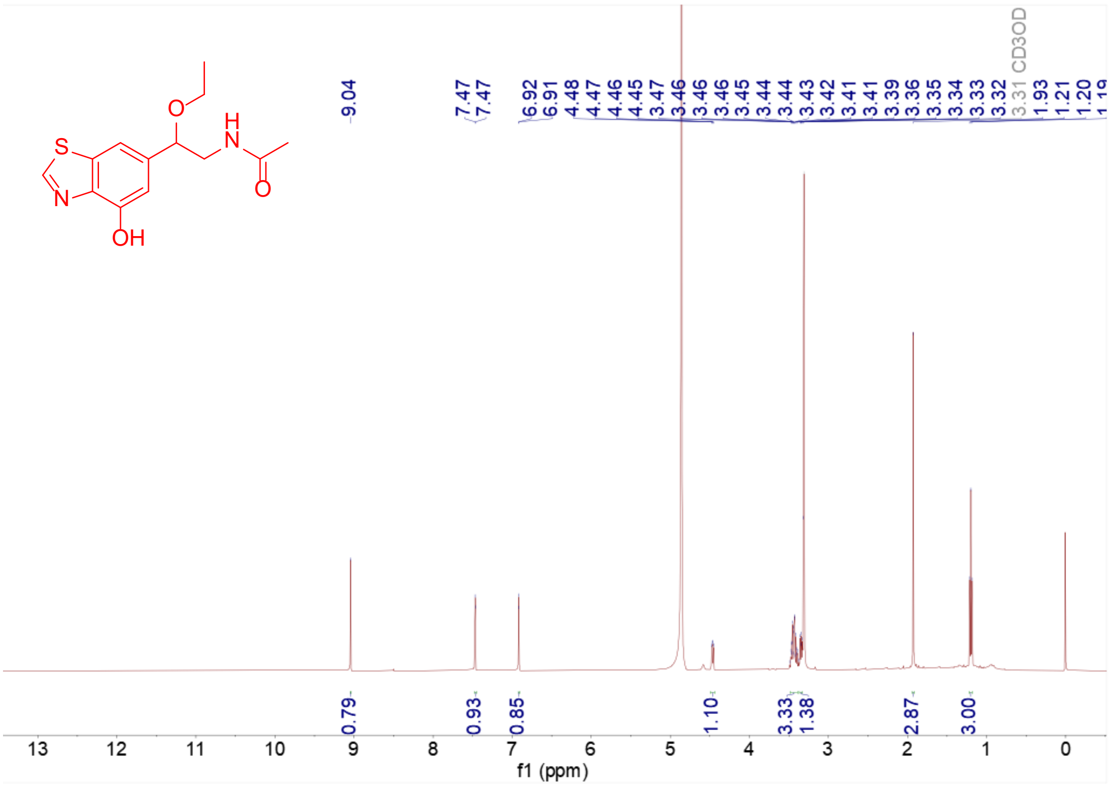

Figure S25. ^{1}H NMR (500 MHz) spectrum of **3** in methanol-d_4.

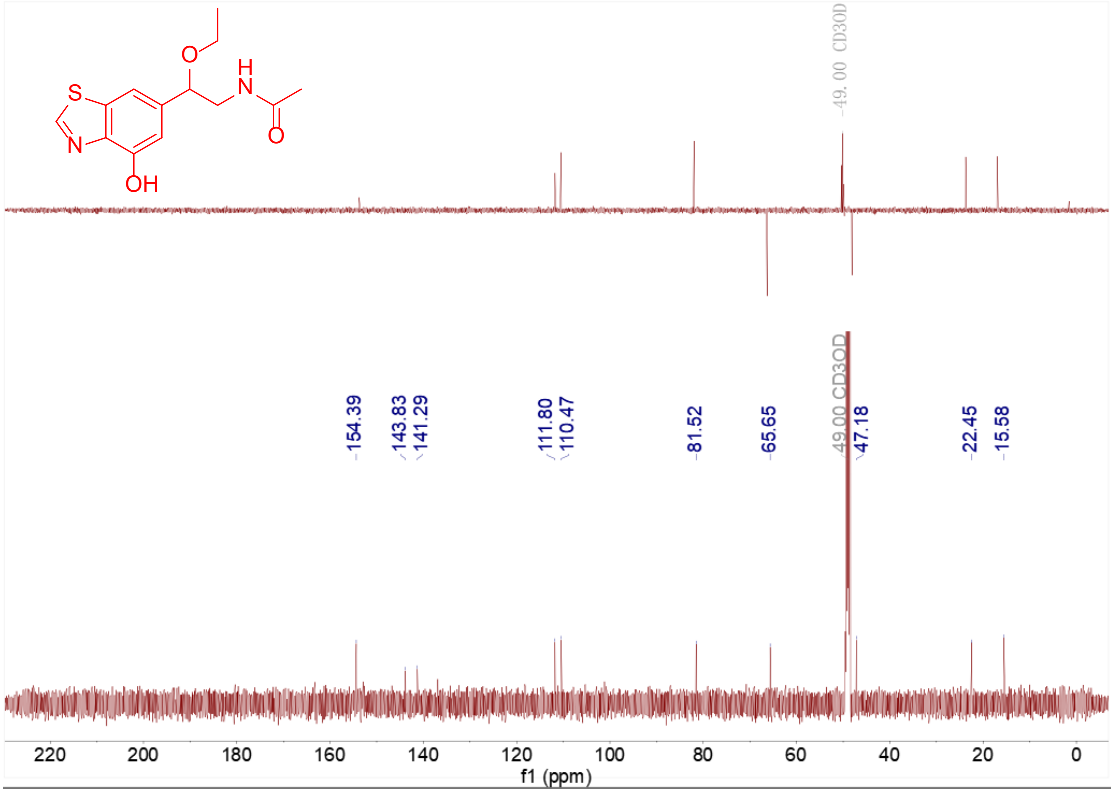

Figure S26. ^{13}C NMR and DEPT-135 (150 MHz) spectra of **3** in methanol-d_4.

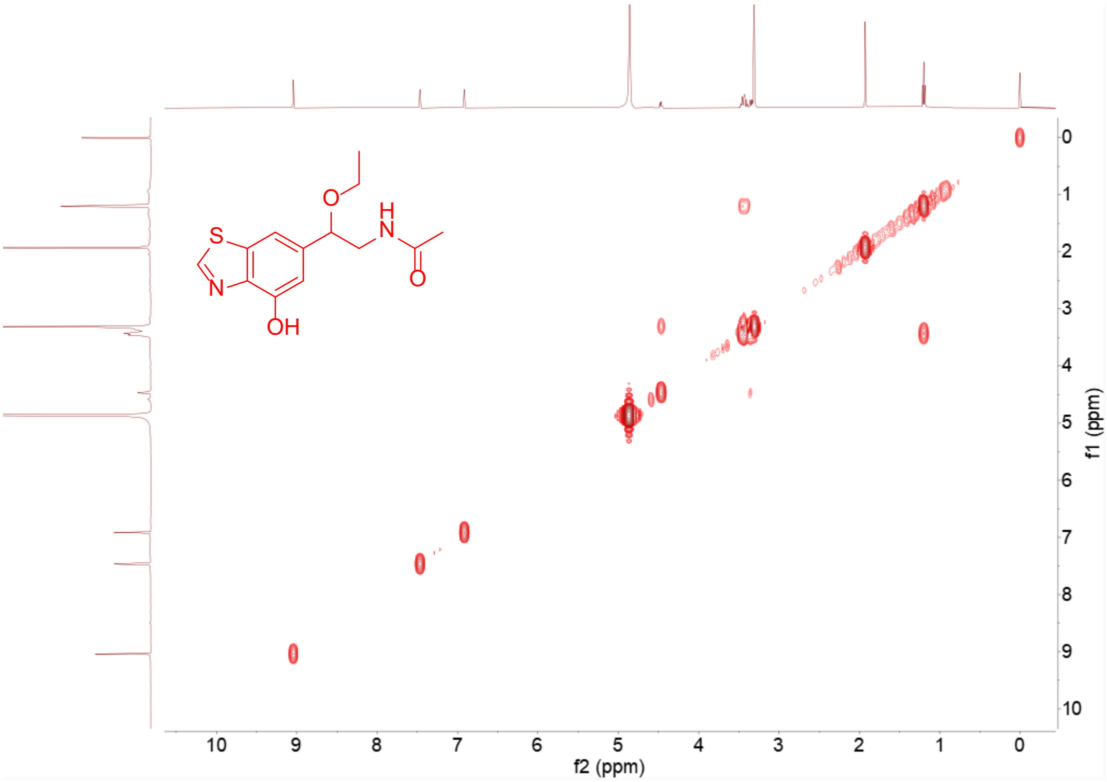

Figure S27. ^{1}H-^{1}H COSY (600 MHz) spectrum of **3** in methanol-*d*4.

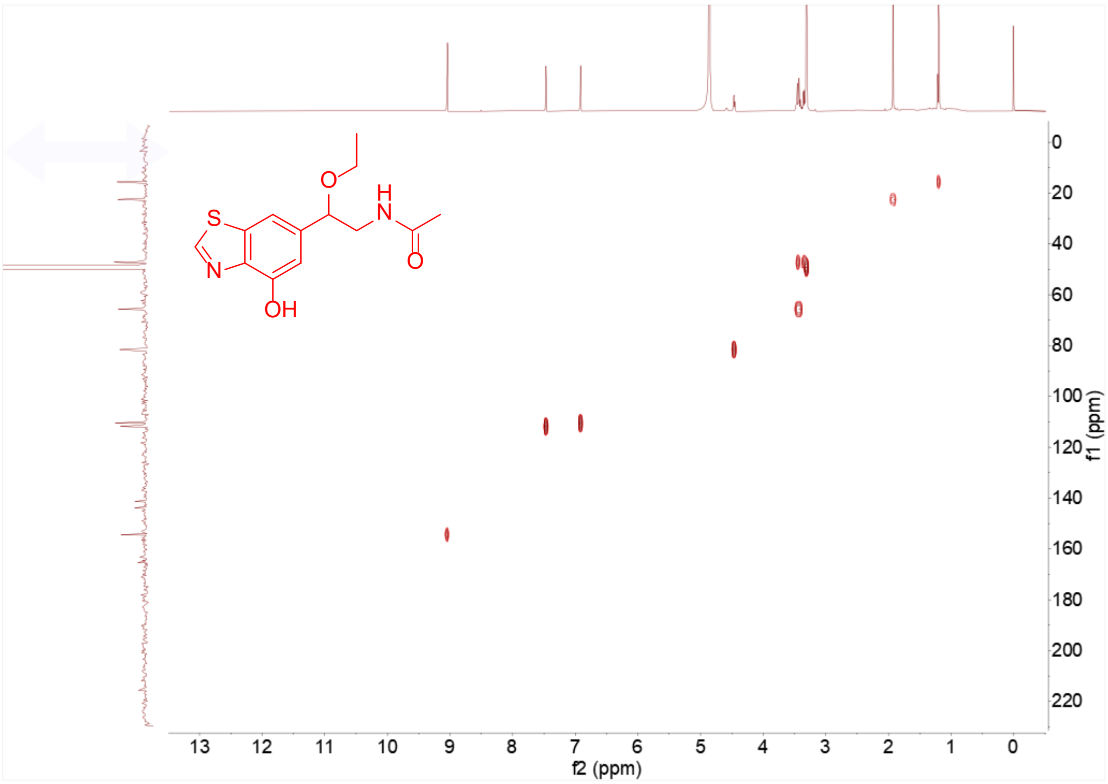

Figure S28. HSQC (600 MHz) spectrum of **3** in methanol-*d*4.

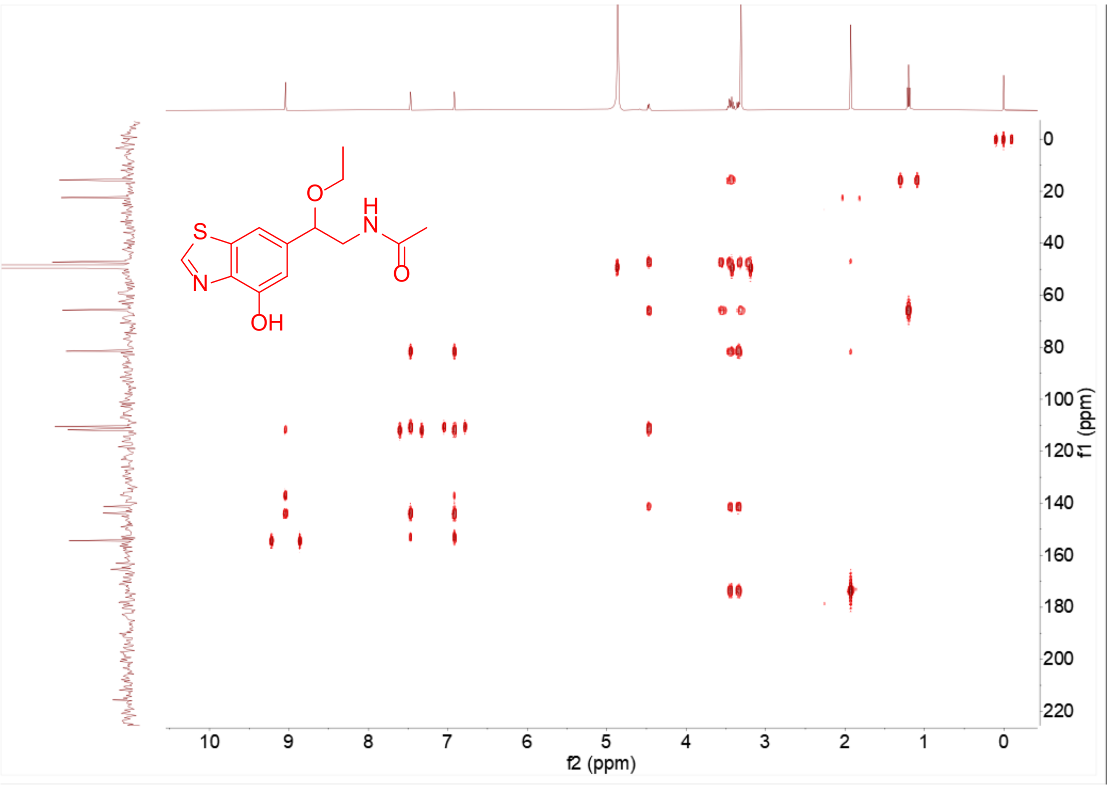

Figure S29. HMBC (600 MHz) spectrum of **3** in methanol-d_4.

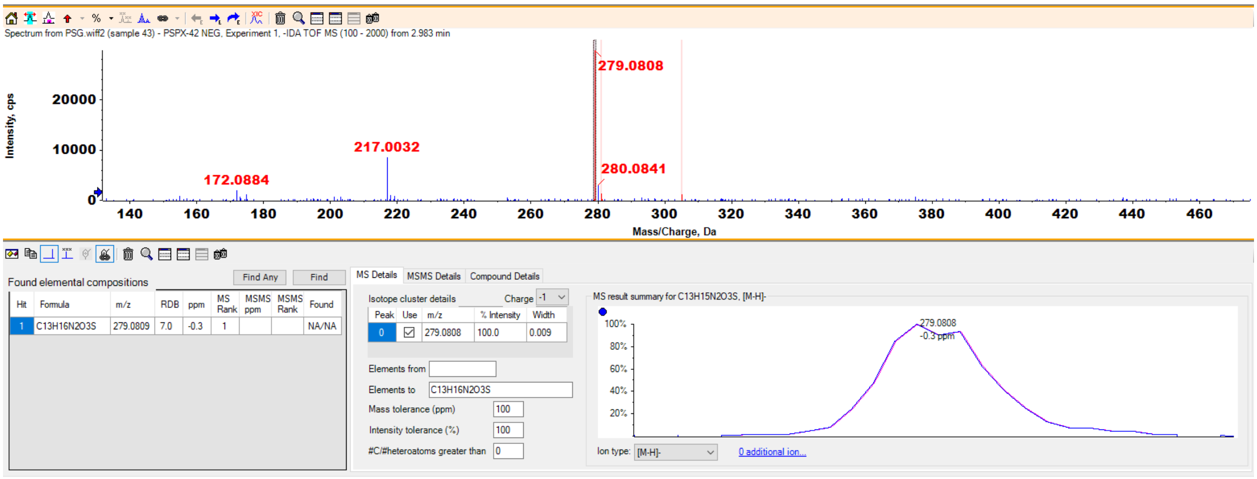

Figure S30. HRESIMS of **3**.

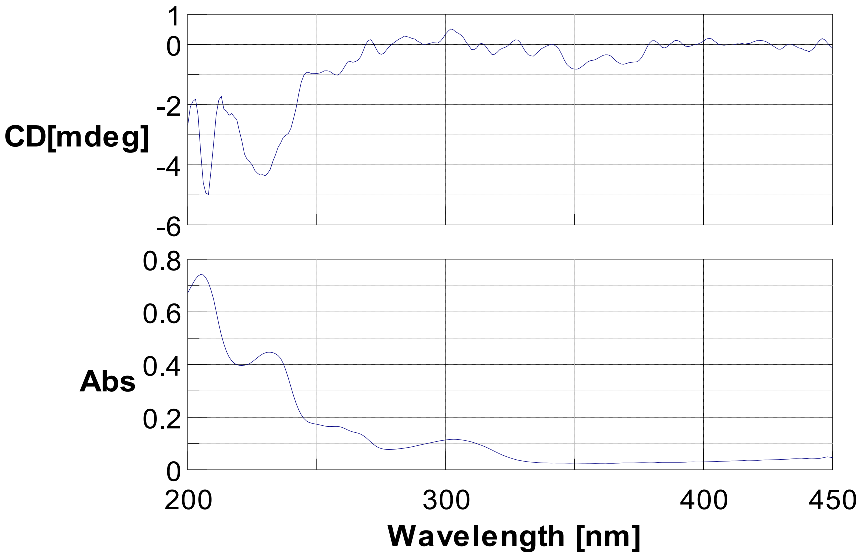

Figure S31. CD and UV spectra of (−)-**3** in methanol.

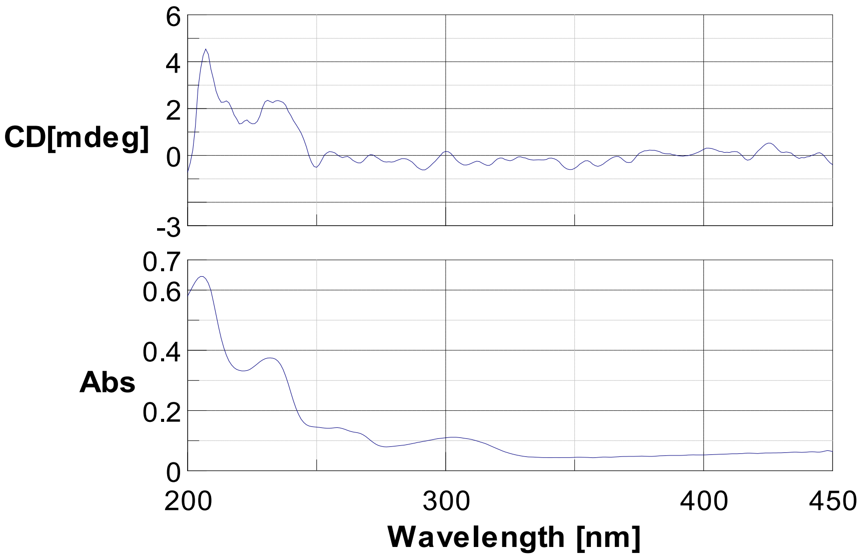

Figure S32. CD and UV spectra of (+)-**3** in methanol.

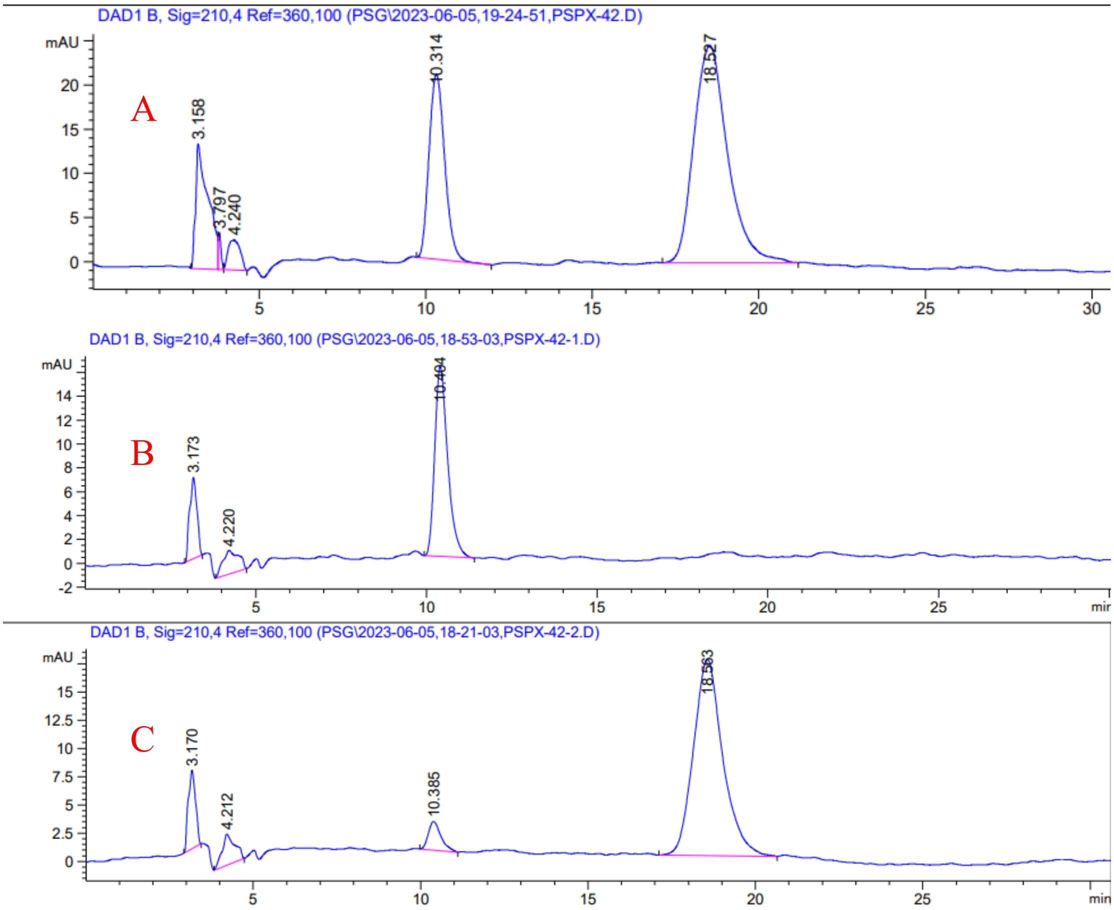

Figure S33. (A) Chiral HPLC chromatogram of **3**;
(B) Chiral HPLC chromatogram of (−)-**3**;
(C) Chiral HPLC chromatogram of (+)-**3**.

Analysis condition: Daicel Chiralpak IC column (250 mm × 4.6 mm, i.d., 5 µm), n-hexane/EtOH, 81:19, 0.05% TFA, flow rate: 1 mL/min.

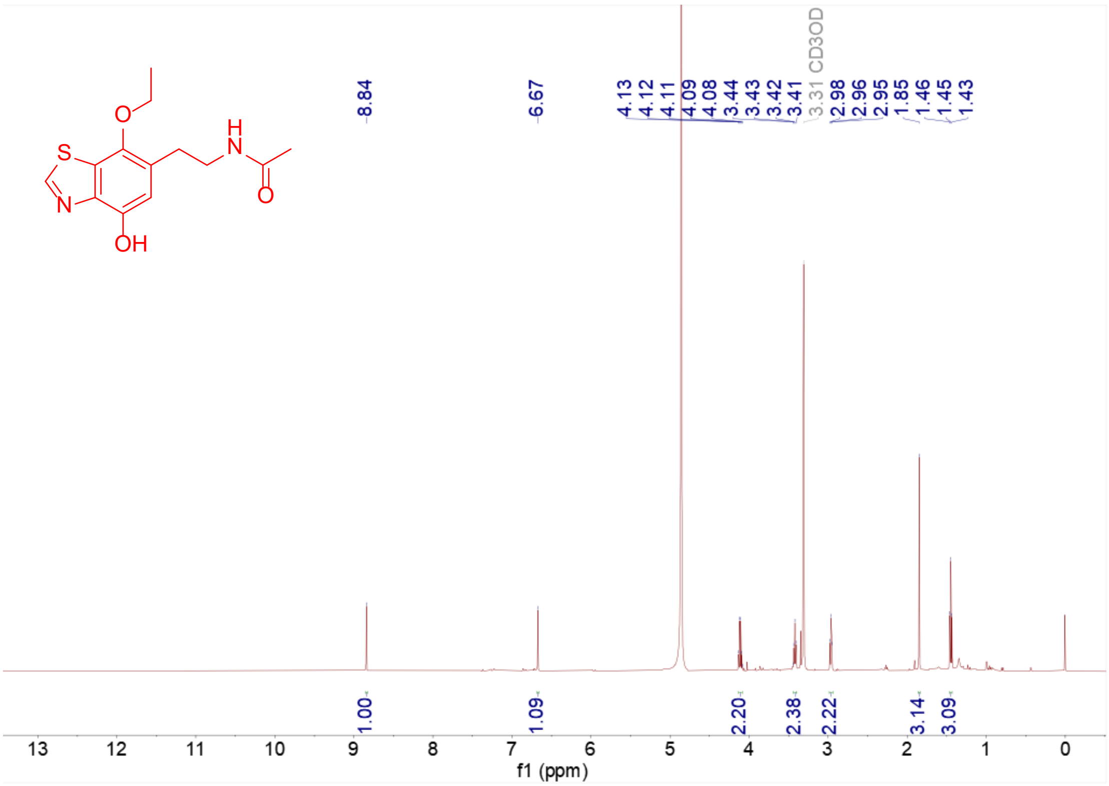

Figure S34. ^{1}H NMR (500 MHz) spectrum of **4** in methanol-d_4.

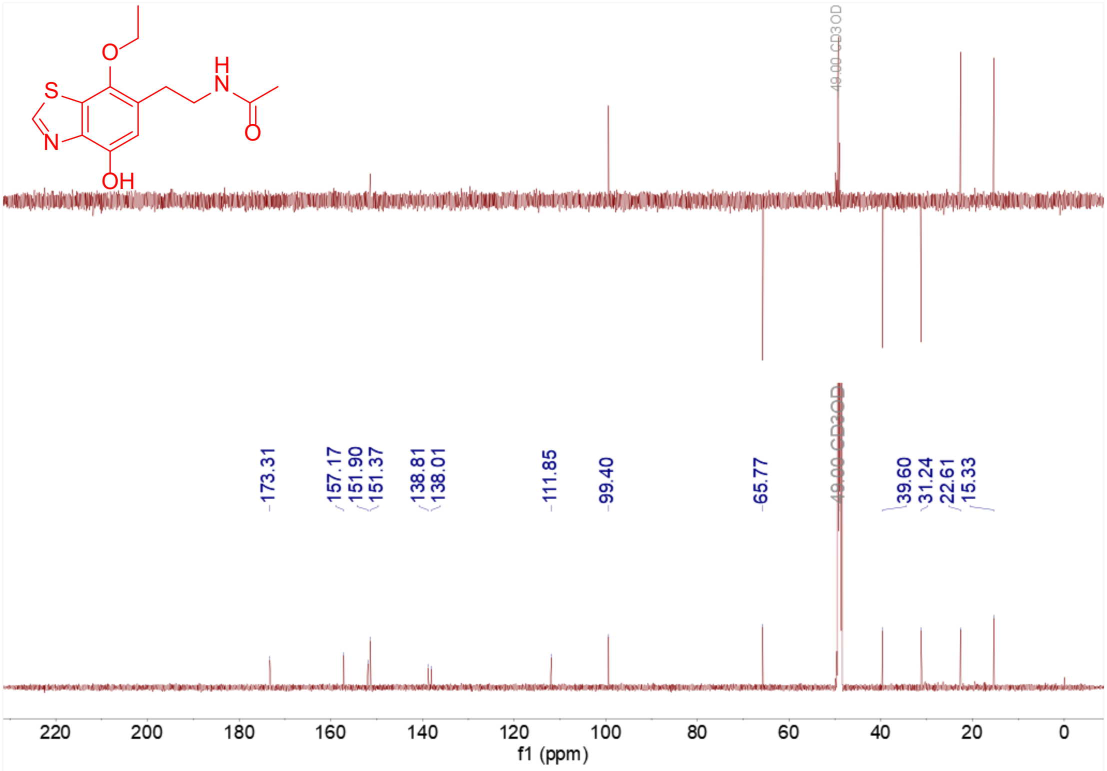

Figure S35. ^{13}C NMR and DEPT-135 (150 MHz) spectra of **4** in methanol-d_4.

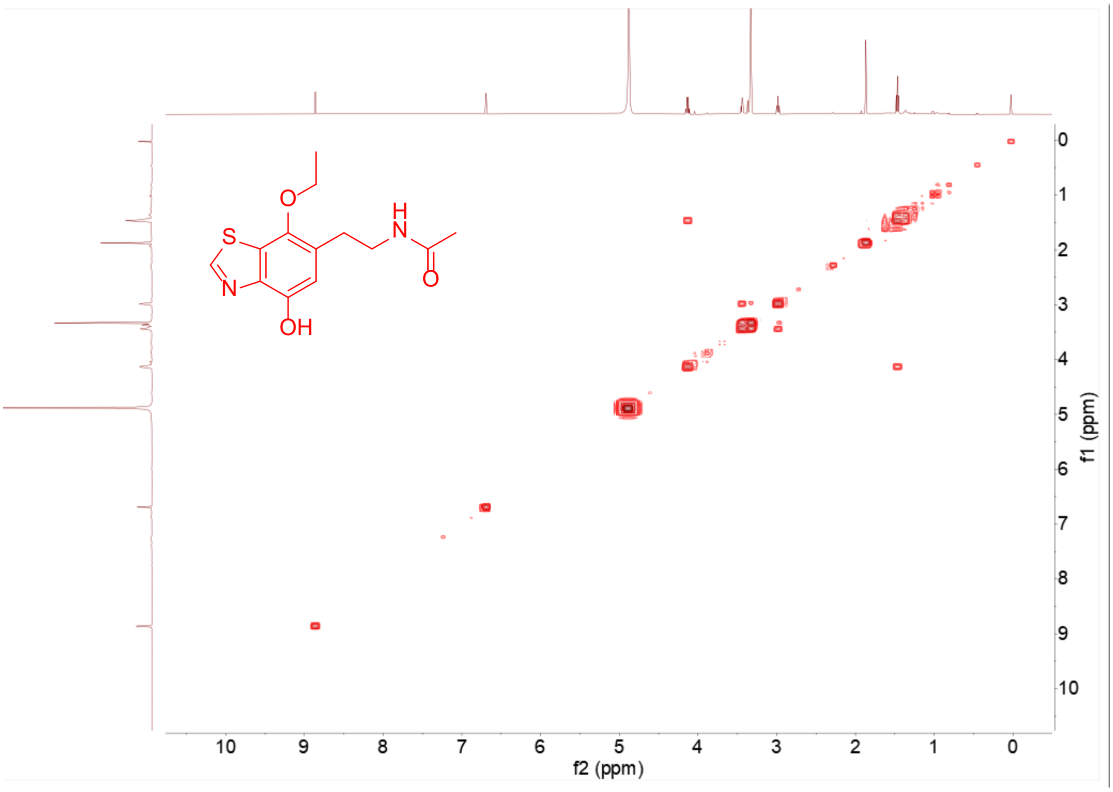

Figure S36. ^{1}H-^{1}H COSY (600 MHz) spectrum of **4** in methanol-d_4.

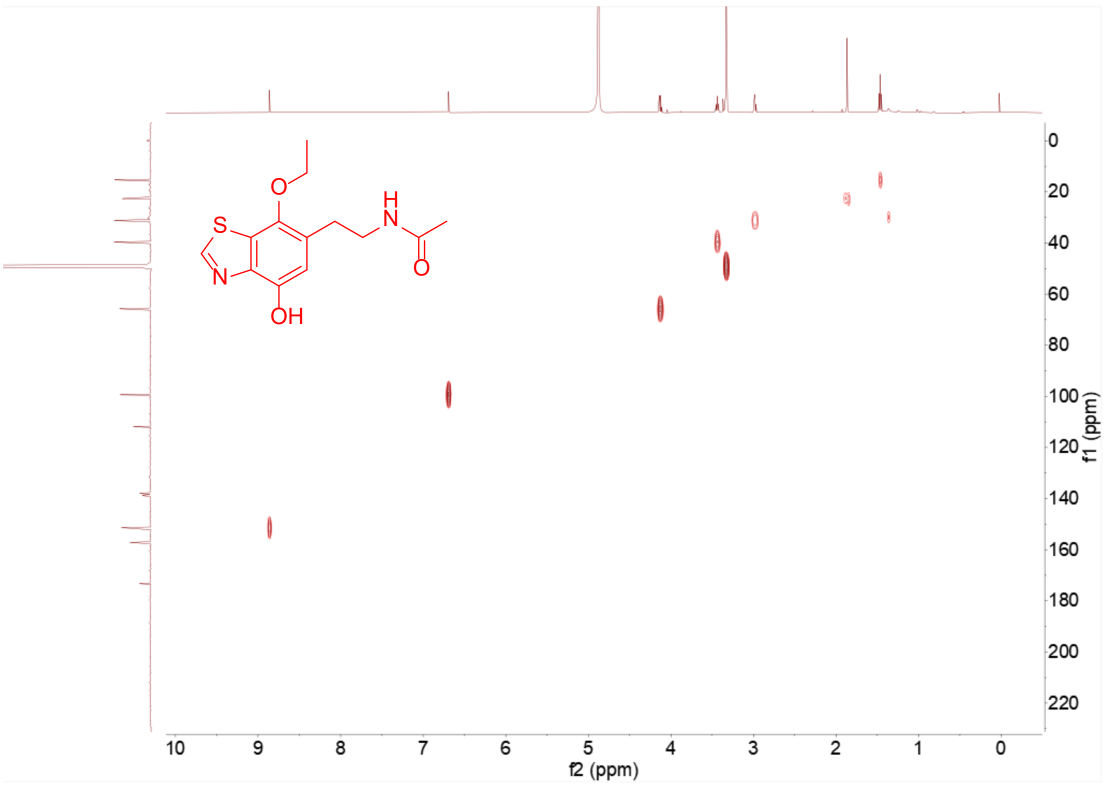

Figure 37. HSQC (600 MHz) spectrum of **4** in methanol-d_4.

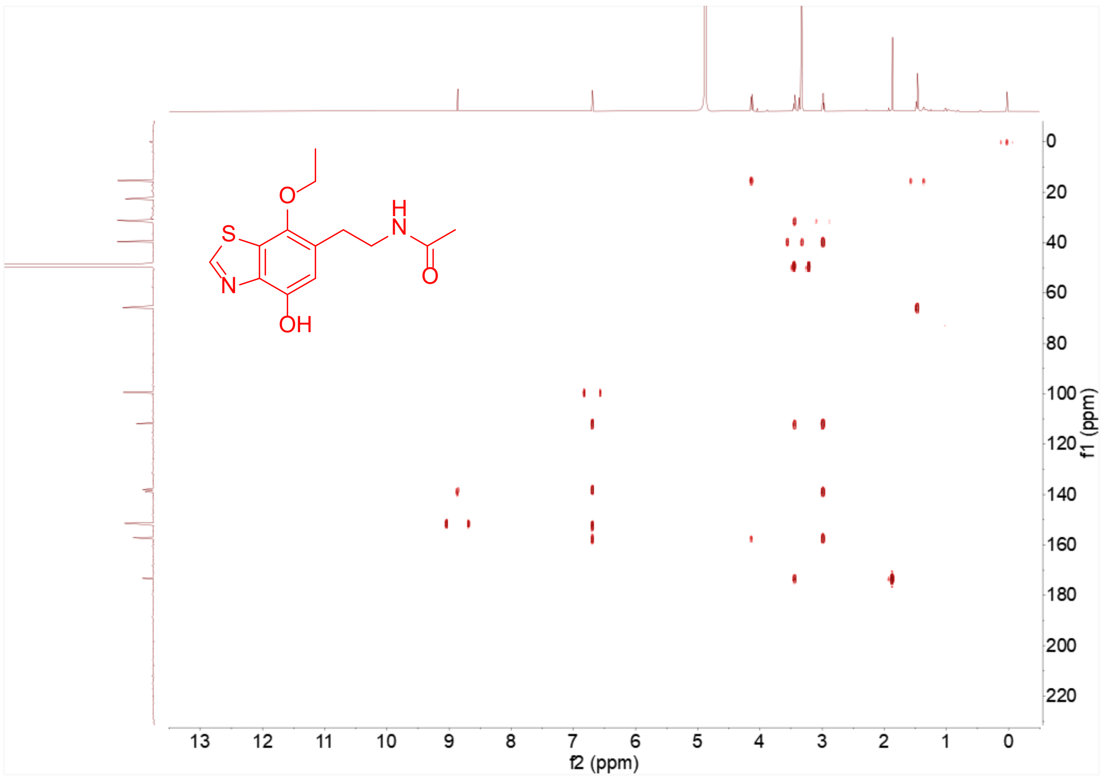

Figure S38. HMBC (600 MHz) spectrum of **4** in methanol-d_4.

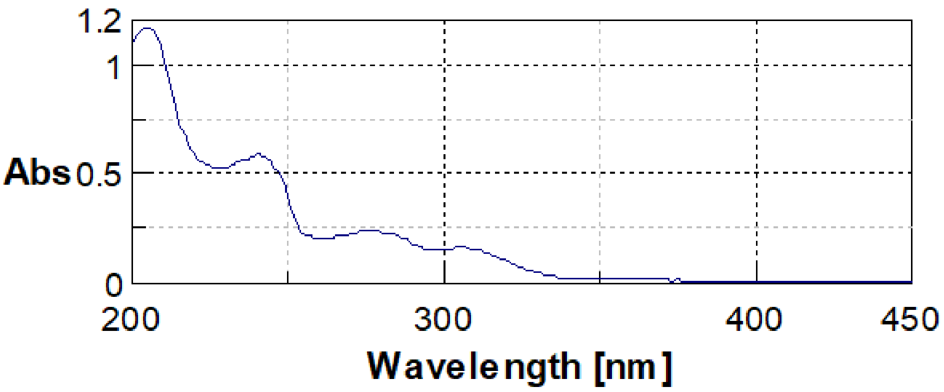

Figure S39. UV spectrum of **4** in methanol.

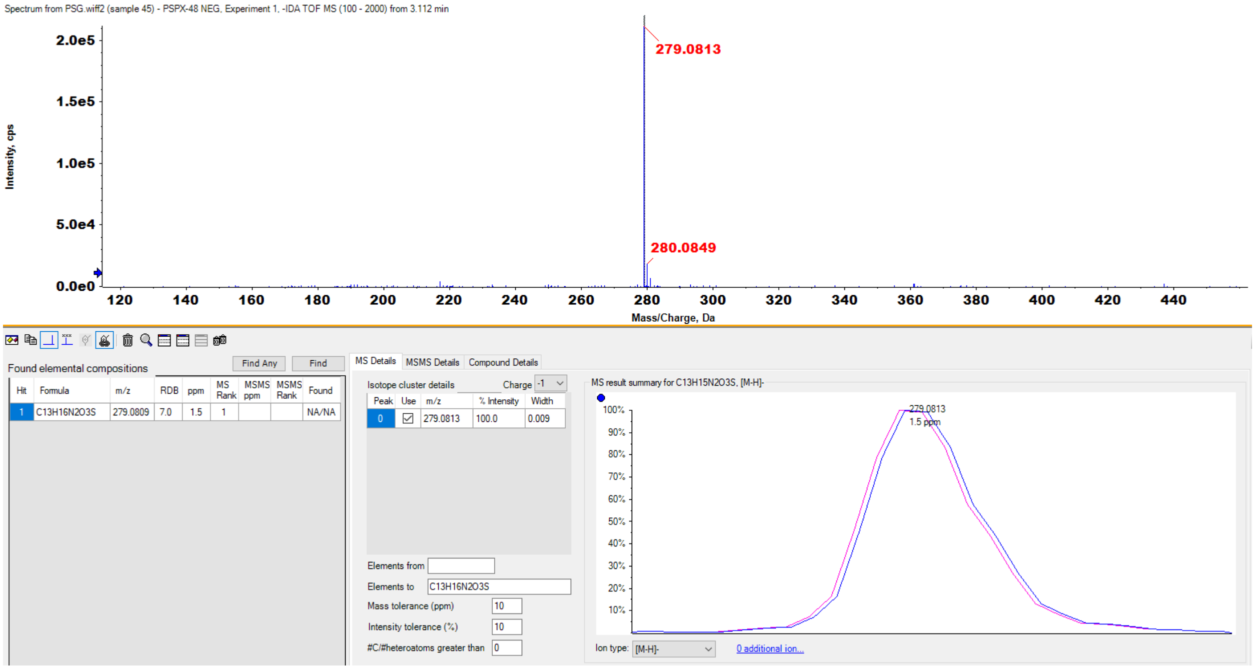

Figure S40. HRESIMS of **4**.

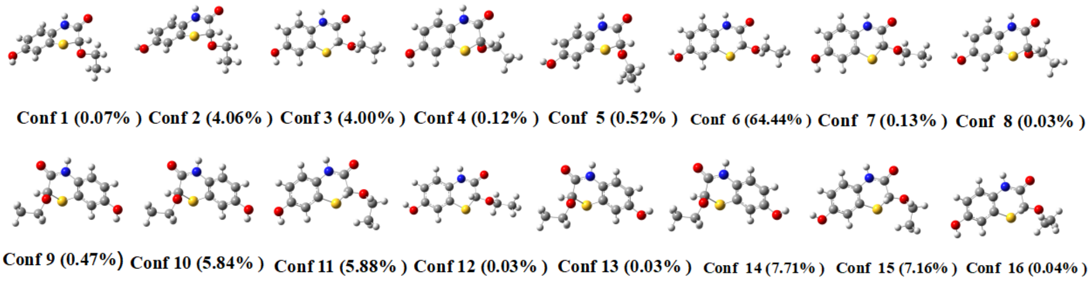

Figure S41. mPW1PW91/6-311+G(d,p) optimized lowest energy conformers for (8*R*)-**1**.

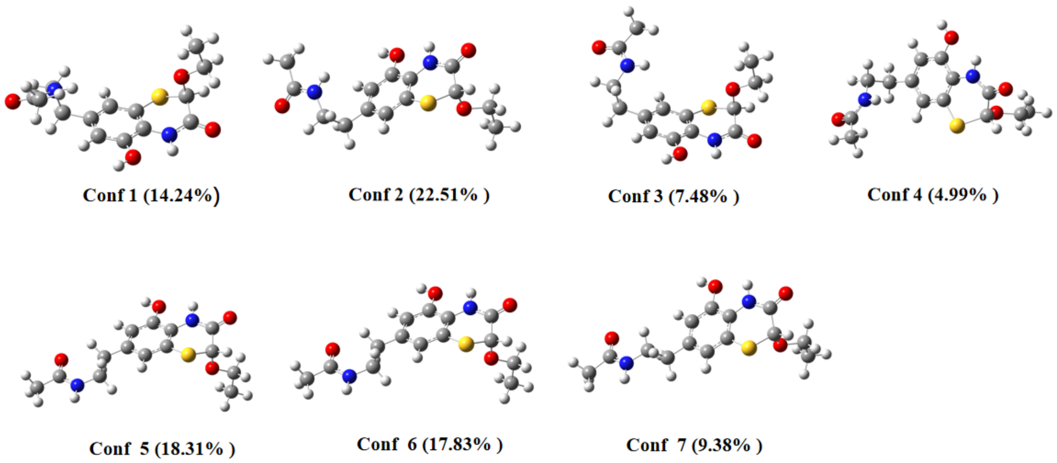

Figure S42. B3LYP/6-31G(d,p) optimized lowest energy conformers for (8*R*)-**2**.

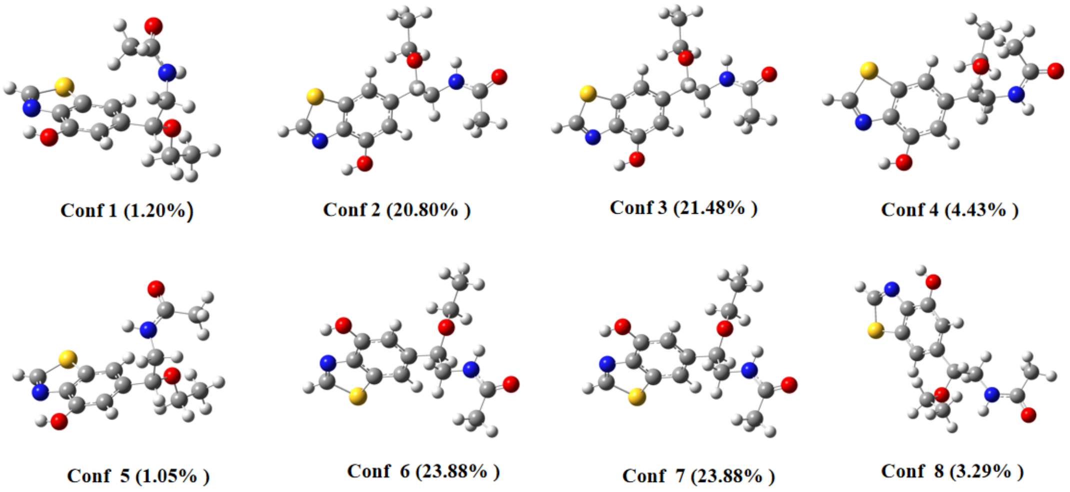

Figure S43. B3LYP/6-31G(d,p) optimized lowest energy conformers for (7*R)*-**3**.

HPLC-MS experiments of the crude extract and the compounds 1–4.

We have applied LC-MS (at positive ion mode) to confirmed that whether these new compounds are artificial products or not. By comparing the retention times (t_R) of HPLC profile and molecular weight of compounds **1–4** with that of the crude extract (raw materials were extracted with acetone and MeOH, respectively), which showed that compounds **1–4** were not detectable in the above extracts (data not shown), confirming that they are artificial products.

Extraction and analytical methods:

The dried and powdered insect bodies of Mantidis Ootheca (5.0 g) were smashed, then ultrasonic-assisted extraction respectively with acetone and MeOH (2 × 50 mL, 1 h). The LC-MS analyses of the extracts of Mantidis Ootheca in acetone and MeOH were performed on an Shimazu LC-20AD AB SCIEX triple TOF X500R MS spectrometer (Shimadzu Corporation, Tokyo, Japan) using a C18 column (2.1 mm × 50 mm, i.d., 5 µm, Waters Corporation, Milford, MA, USA). The run started with 90% water and 10% acetonitrile, increasing to 100% acetonitrile in 50 min, held until 56.5 min, then returning to initial conditions and stabilizing up to 60 min. Taking compounds **1–4** as the standard samples by running the same LC-MS conditions as above.

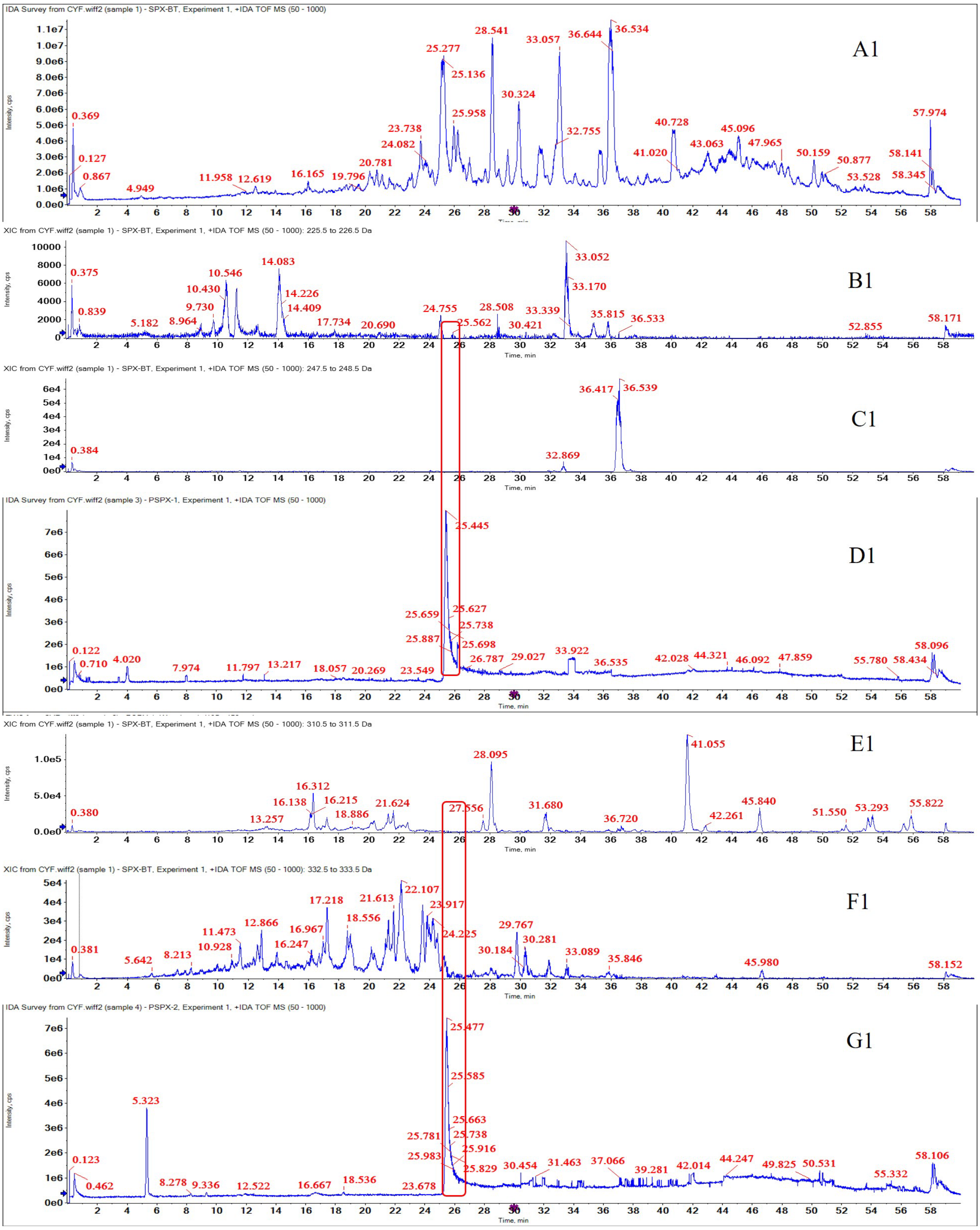

Figure S44. LC-MS chromatogram of the crude extract and the compounds (**1** and **2**). To check whether compounds **1** and **2** are natural products or artifacts, Mantidis Ootheca was extracted with **acetone** (for details, see above Extraction). **A1**: HPLC chromatogram of crude extract; **B1**: LC-MS spectrum of crude extract targeting m/z 226; **C1**: LC-MS spectrum of crude extract targeting m/z 248; **D1**: HPLC chromatogram of compound **1**; **E1**: LC-MS spectrum of crude extract targeting m/z 311; **F1**: LC-MS spectrum of crude extract targeting m/z 333; **G1**: HPLC chromatogram of compound **2**;

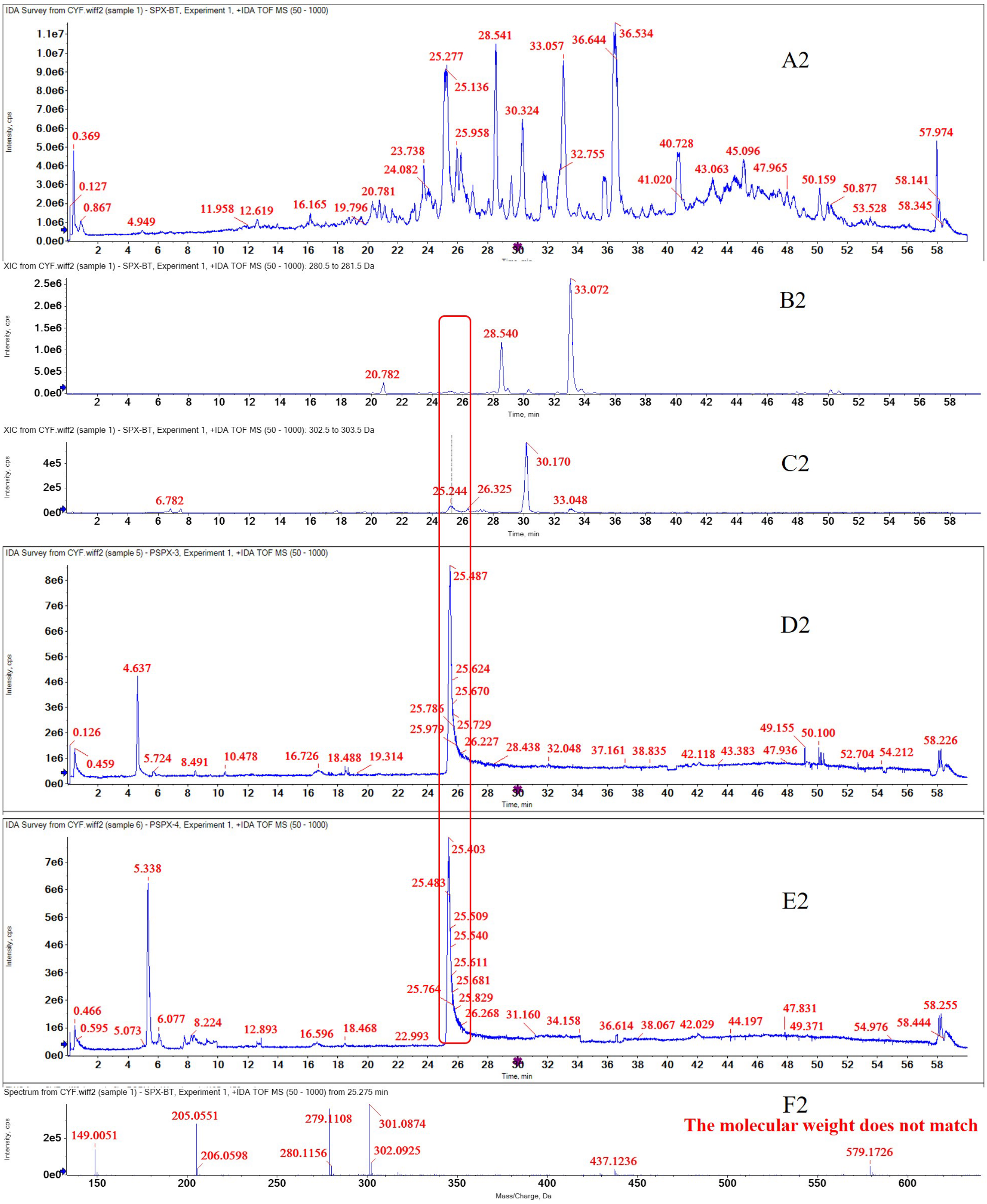

Figure S45. LC-MS chromatogram of the crude extract and the compounds (**3** and **4**). To check whether compounds **3** and **4** are natural products or artifacts, Mantidis Ootheca was extracted with **acetone** (for details, see above Extraction). **A2**: HPLC chromatogram of crude extract; **B2**: LC-MS spectrum of crude extract targeting m/z 281; **C2**: LC-MS spectrum of crude extract targeting m/z 303; **D2**: HPLC chromatogram of compound **3**; **E2**: HPLC chromatogram of compound 4; **F2**: LC-MS spectrum of the fractions that from 25.2 minutes in **C2**;

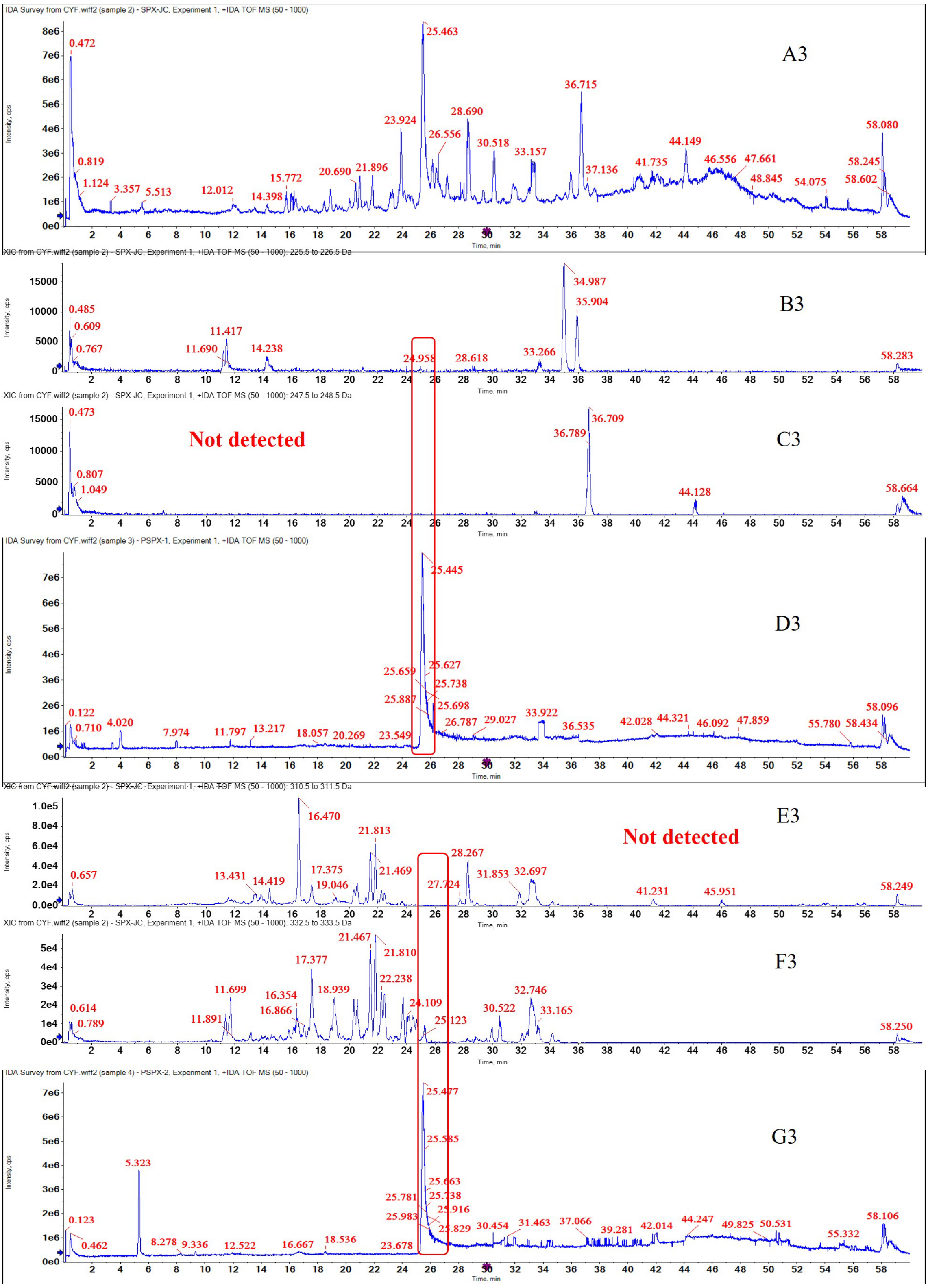

Figure S46. LC-MS chromatogram of the crude extract and the compounds (**1** and **2**). To check whether compounds **1** and **2** are natural products or artifacts, Mantidis Ootheca was extracted with **MeOH** (for details, see above Extraction). **A3**: HPLC chromatogram of crude extract; **B3**: LC-MS spectrum of crude extract targeting m/z 226; **C3**: LC-MS spectrum of crude extract targeting m/z 248; **D3**: HPLC chromatogram of compound **1**; **E3**: LC-MS spectrum of crude extract targeting m/z 311; **F3**: LC-MS spectrum of crude extract targeting

m/z 333; **G3**: HPLC chromatogram of compound **2**;

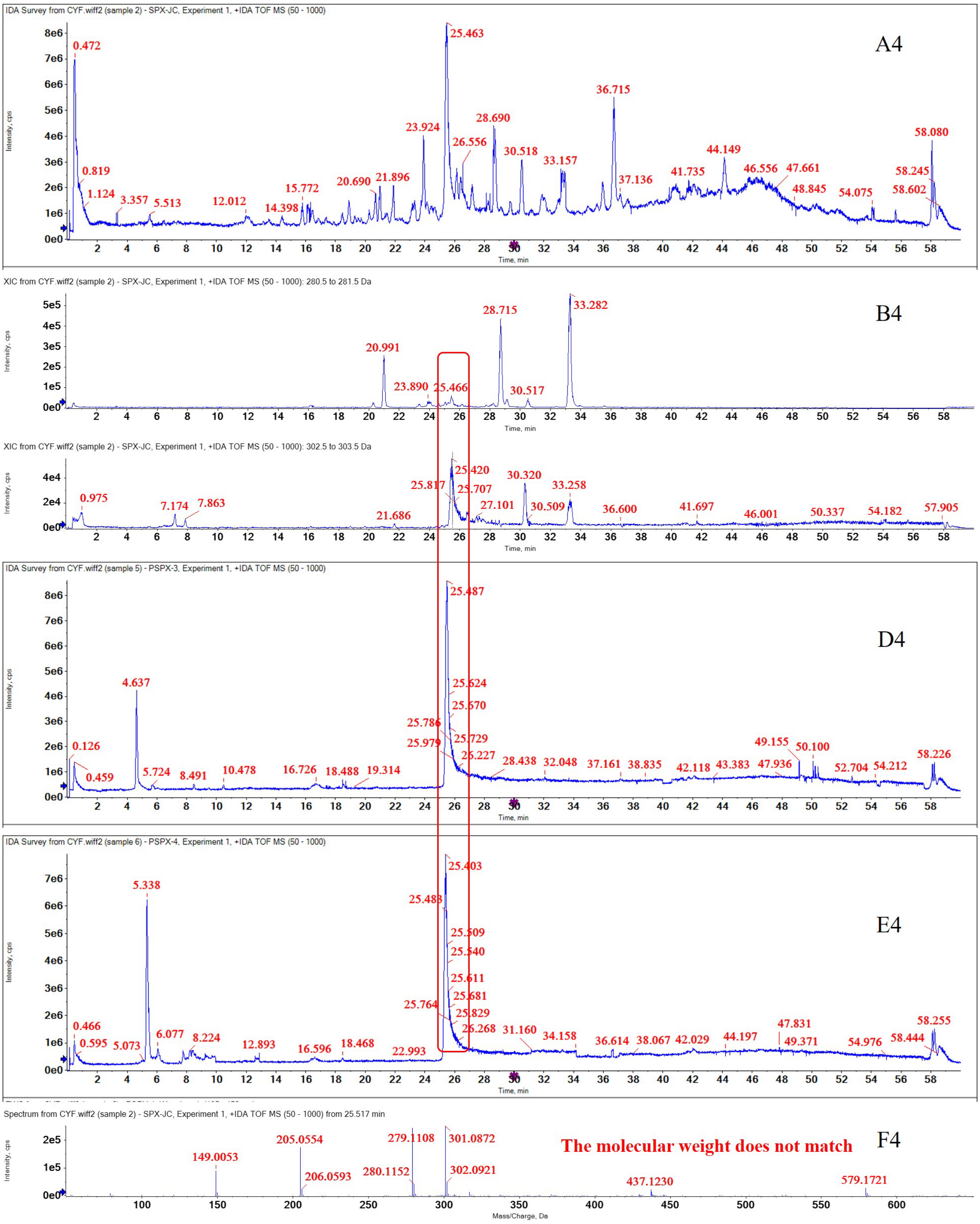

Figure S47. LC-MS chromatogram of the crude extract and the compounds (**3** and **4**). To check whether compounds **3** and **4** are natural products or artifacts, Mantidis Ootheca was extracted with **MeOH** (for details, see above Extraction). **A4**: HPLC chromatogram of crude extract; **B4**: LC-MS spectrum of crude extract targeting m/z 281; **C4**: LC-MS spectrum of crude extract targeting m/z 303; **D4**: HPLC chromatogram of compound **3**; **E4**: HPLC chromatogram of compound 4; **F4**: LC-MS spectrum of the fractions that from 25.5 minutes in **C4**;

Table S1 The Cartesian coordinates of the lowest energy conformers for (8*R*)-**1**.

**Conf 1**	**X axis(Å)**	**Y axis(Å)**	**Z axis(Å)**	**Conf 2**	**X axis(Å)**	**Y axis(Å)**	**Z axis(Å)**
C	-3.2202	-0.4539	-0.3002	C	-3.2231	-0.4586	-0.2956
C	-3.0682	0.6542	-1.1242	C	-3.0775	0.6518	-1.1193
C	-1.8691	1.3671	-1.1101	C	-1.8765	1.364	-1.1026
C	-0.8196	0.9565	-0.2816	C	-0.8237	0.9526	-0.277
C	-0.9836	-0.1566	0.5576	C	-0.9825	-0.1631	0.5586
C	-2.1937	-0.8613	0.5465	C	-2.1924	-0.865	0.5457
N	0.3568	1.7197	-0.2877	N	0.3505	1.7192	-0.2832
C	1.5436	1.371	0.3026	C	1.5393	1.3719	0.3046
S	0.2695	-0.6393	1.7291	S	0.2745	-0.6492	1.7233
O	2.4687	2.1891	0.3199	O	2.4613	2.1936	0.3218
C	1.7316	-0.0738	0.8048	C	1.7328	-0.0734	0.8018
O	2.0125	-0.9852	-0.2567	O	2.018	-0.9742	-0.268
C	3.3494	-0.8707	-0.7379	C	3.364	-0.8725	-0.7254
C	3.5515	-1.908	-1.8252	C	3.5659	-1.8888	-1.8323
O	-4.4109	-1.1164	-0.3446	O	-4.3803	-1.179	-0.2749
H	-3.8826	0.9633	-1.7739	H	-3.8811	0.9779	-1.7723
H	-1.7649	2.233	-1.7581	H	-1.7706	2.2332	-1.7467
H	-2.3265	-1.7162	1.203	H	-2.3417	-1.7258	1.1926
H	0.3619	2.6275	-0.7567	H	0.3539	2.6288	-0.7481
H	2.5584	-0.0783	1.5247	H	2.5599	-0.0774	1.5214
H	3.5191	0.1289	-1.1505	H	3.5579	0.133	-1.1122
H	4.0569	-1.0497	0.079	H	4.0547	-1.0822	0.0985
H	4.5677	-1.8598	-2.2265	H	4.5892	-1.8497	-2.2161
H	3.3727	-2.9154	-1.435	H	3.3633	-2.9013	-1.4679
H	2.8404	-1.7522	-2.6433	H	2.8712	-1.7023	-2.658
H	-4.3729	-1.8722	0.2653	H	-5.0042	-0.7953	-0.9128
**Conf 3**	**X axis(Å)**	**Y axis(Å)**	**Z axis(Å)**	**Conf 4**	**X axis(Å)**	**Y axis(Å)**	**Z axis(Å)**
C	-3.5419	-0.4568	0.1939	C	-3.2769	-0.4822	-0.2819
C	-3.5482	0.9246	0.3401	C	-3.2354	0.8186	-0.7675
C	-2.3546	1.6375	0.2251	C	-2.0615	1.5633	-0.6501
C	-1.1548	0.9607	-0.0163	C	-0.9273	0.9955	-0.0605
C	-1.1557	-0.4336	-0.1709	C	-0.979	-0.3152	0.439
C	-2.3596	-1.142	-0.0684	C	-2.1639	-1.0535	0.3275
N	0.0125	1.7252	-0.1471	N	0.2202	1.7931	0.0533
C	1.297	1.2445	-0.2091	C	1.467	1.3601	0.4228
S	0.3204	-1.3305	-0.6034	S	0.3916	-1.0386	1.3185
O	2.2046	2.0435	-0.4555	O	2.3638	2.192	0.5936
C	1.549	-0.2342	0.1798	C	1.7445	-0.1532	0.486
O	2.8544	-0.7071	-0.1331	O	1.9489	-0.6951	-0.8189
C	3.8241	-0.3556	0.8491	C	3.2778	-0.5291	-1.3068
C	5.1773	-0.8536	0.3807	C	4.1764	-1.6506	-0.8162

O	-4.7331	-1.1096	0.3057	O	-4.4466	-1.1698	-0.4145
H	-4.4819	1.4455	0.5356	H	-4.1159	1.2524	-1.2341
H	-2.3762	2.7187	0.3337	H	-2.0439	2.5803	-1.0324
H	-2.3658	-2.2198	-0.2036	H	-2.2095	-2.0638	0.7232
H	-0.0629	2.7401	-0.247	H	0.1553	2.7941	-0.1413
H	1.384	-0.313	1.2623	H	2.6303	-0.3065	1.1117
H	3.8589	0.7291	0.9888	H	3.2236	-0.5737	-2.3995
H	3.5679	-0.8293	1.8032	H	3.6836	0.4525	-1.0404
H	5.9557	-0.6127	1.1102	H	5.1718	-1.5708	-1.2627
H	5.1582	-1.9379	0.2291	H	4.2839	-1.6293	0.2723
H	5.4421	-0.4018	-0.581	H	3.7502	-2.6253	-1.0754
H	-4.5897	-2.0589	0.1535	H	-4.3295	-2.0626	-0.049
**Conf 5**	**X axis(Å)**	**Y axis(Å)**	**Z axis(Å)**	**Conf 6**	**X axis(Å)**	**Y axis(Å)**	**Z axis(Å)**
C	-3.1502	-0.6067	-0.158	C	-3.541	-0.4614	0.194
C	-3.1557	0.5091	-0.9872	C	-3.5541	0.9215	0.3343
C	-2.0209	1.3201	-1.0514	C	-2.3582	1.6326	0.2177
C	-0.8829	1.0019	-0.301	C	-1.1557	0.9567	-0.0204
C	-0.8889	-0.1208	0.5406	C	-1.1529	-0.4372	-0.1724
C	-2.0334	-0.9221	0.6093	C	-2.356	-1.1425	-0.0655
N	0.2191	1.8647	-0.3863	N	0.0104	1.7233	-0.1483
C	1.4711	1.6218	0.1184	C	1.2959	1.2444	-0.2051
S	0.483	-0.4994	1.6123	S	0.324	-1.3306	-0.6065
O	2.3143	2.5237	0.0837	O	2.2026	2.0474	-0.4433
C	1.8163	0.2064	0.6039	C	1.5499	-0.2351	0.1789
O	2.0903	-0.6046	-0.5427	O	2.8573	-0.701	-0.1372
C	3.1659	-1.5121	-0.3241	C	3.8236	-0.3608	0.8521
C	3.3341	-2.3598	-1.5694	C	5.1797	-0.8463	0.3793
O	-4.2381	-1.4224	-0.0597	O	-4.6849	-1.1955	0.295
H	-4.0266	0.7641	-1.583	H	-4.4773	1.4592	0.528
H	-2.0323	2.1926	-1.6995	H	-2.3767	2.7146	0.3227
H	-2.0656	-1.7901	1.2628	H	-2.3808	-2.222	-0.1942
H	0.1177	2.7674	-0.8532	H	-0.0642	2.738	-0.2483
H	2.7058	0.2982	1.2393	H	1.3861	-0.3174	1.2613
H	4.0872	-0.9515	-0.1311	H	3.8536	0.7219	1.0082
H	2.9487	-2.1553	0.5354	H	3.5671	-0.8498	1.7984
H	4.1548	-3.0723	-1.4486	H	5.9556	-0.6135	1.114
H	2.4142	-2.9131	-1.7859	H	5.1652	-1.9283	0.2113
H	3.5388	-1.7289	-2.4408	H	5.4448	-0.379	-0.5749
H	-4.9352	-1.0931	-0.6507	H	-5.4347	-0.5978	0.4506
**Conf 7**	**X axis(Å)**	**Y axis(Å)**	**Z axis(Å)**	**Conf 8**	**X axis(Å)**	**Y axis(Å)**	**Z axis(Å)**
C	-3.496	-0.5446	-0.0095	C	-3.2767	-0.4916	-0.2755
C	-3.5773	0.8415	-0.0326	C	-3.2441	0.8094	-0.7644
C	-2.4081	1.6018	-0.0608	C	-2.0695	1.5553	-0.6481

C	-1.1597	0.9705	-0.048	C	-0.9308	0.9916	-0.0603
C	-1.0837	-0.4298	-0.0339	C	-0.9751	-0.3186	0.4393
C	-2.262	-1.1875	-0.0179	C	-2.1588	-1.0561	0.3301
N	-0.0192	1.7828	-0.0985	N	0.2132	1.7948	0.051
C	1.2805	1.3818	0.0814	C	1.4626	1.3673	0.4188
S	0.4695	-1.2923	-0.1424	S	0.4004	-1.0376	1.3129
O	2.1719	2.2126	-0.1114	O	2.3554	2.2045	0.5859
C	1.5476	-0.0427	0.6405	C	1.7475	-0.144	0.4827
O	2.8888	-0.509	0.7373	O	1.9565	-0.6781	-0.8251
C	3.649	-0.4118	-0.4641	C	3.2928	-0.529	-1.2975
C	4.9073	-1.242	-0.2901	C	4.1682	-1.6688	-0.807
O	-4.6643	-1.2462	0.011	O	-4.404	-1.2533	-0.362
H	-4.5489	1.3286	-0.0337	H	-4.1156	1.2577	-1.2314
H	-2.4878	2.6856	-0.0839	H	-2.0517	2.5729	-1.0295
H	-2.2072	-2.2725	-0.0208	H	-2.2196	-2.0691	0.7199
H	-0.1249	2.7787	-0.3066	H	0.1453	2.7953	-0.1431
H	1.2063	0.0099	1.6822	H	2.6341	-0.2923	1.1086
H	3.0836	-0.7834	-1.3245	H	3.2499	-0.5632	-2.3911
H	3.9341	0.6279	-0.6444	H	3.7107	0.4439	-1.0178
H	5.534	-1.1927	-1.185	H	5.17	-1.6	-1.2406
H	5.4881	-0.8878	0.5681	H	4.2625	-1.6597	0.2829
H	4.6536	-2.289	-0.0936	H	3.7303	-2.6343	-1.0808
H	-4.4633	-2.1971	0.0165	H	-5.097	-0.744	-0.8133
**Conf 9**	**X axis(Å)**	**Y axis(Å)**	**Z axis(Å)**	**Conf 10**	**X axis(Å)**	**Y axis(Å)**	**Z axis(Å)**
C	3.2239	0.0029	0.4189	C	3.3053	-0.3018	0.3347
C	2.9582	1.3637	0.3308	C	3.1638	1.0186	0.7424
C	1.6999	1.7969	-0.0879	C	1.9417	1.6686	0.5685
C	0.7062	0.8638	-0.4009	C	0.8599	0.9874	0.0011
C	0.9845	-0.5096	-0.3217	C	1.0121	-0.3433	-0.4191
C	2.253	-0.9378	0.0887	C	2.245	-0.9864	-0.2515
N	-0.5346	1.3495	-0.8373	N	-0.3389	1.6931	-0.1739
C	-1.6757	0.6076	-0.9998	C	-1.5459	1.1518	-0.5343
S	-0.2002	-1.7358	-0.8396	S	-0.291	-1.2157	-1.2664
O	-2.6725	1.1413	-1.4964	O	-2.4914	1.9079	-0.7781
C	-1.7229	-0.8246	-0.43	C	-1.7142	-0.376	-0.5097
O	-1.9383	-0.8628	0.9791	O	-1.889	-0.82	0.8368
C	-3.247	-0.454	1.37	C	-3.1727	-1.3756	1.1039
C	-3.1673	0.8789	2.09	C	-4.2427	-0.304	1.2108
O	4.4687	-0.3732	0.8281	O	4.5191	-0.8942	0.52
H	3.7302	2.085	0.585	H	4.0038	1.5412	1.1922
H	1.5068	2.8641	-0.1543	H	1.8456	2.7022	0.8899
H	2.4737	-1.9999	0.14	H	2.3687	-2.0122	-0.586
H	-0.63	2.3393	-1.0724	H	-0.3492	2.7056	-0.0376

H	-2.5323	-1.3649	-0.9349	H	-2.5825	-0.6232	-1.1317
H	-3.9413	-0.4022	0.5238	H	-3.4309	-2.1277	0.3497
H	-3.6288	-1.2115	2.0622	H	-3.0978	-1.8952	2.0649
H	-4.1527	1.182	2.4549	H	-5.1894	-0.7376	1.546
H	-2.4802	0.8163	2.9404	H	-3.9398	0.4758	1.9169
H	-2.7838	1.6622	1.4296	H	-4.415	0.1827	0.2464
H	4.5099	-1.3437	0.8578	H	4.4723	-1.8123	0.2049
**Conf 11**	**X axis(Å)**	**Y axis(Å)**	**Z axis(Å)**	**Conf 12**	**X axis(Å)**	**Y axis(Å)**	**Z axis(Å)**
C	-3.4439	-0.5016	0.2248	C	-3.4966	-0.5467	-0.0089
C	-3.4617	0.851	0.5404	C	-3.5824	0.84	-0.0349
C	-2.2863	1.5971	0.4521	C	-2.4097	1.5972	-0.0637
C	-1.0919	0.9799	0.0665	C	-1.1599	0.966	-0.0493
C	-1.0816	-0.3847	-0.2587	C	-1.082	-0.4335	-0.0335
C	-2.2681	-1.1249	-0.1815	C	-2.2613	-1.1863	-0.0159
N	0.0556	1.7786	-0.0288	N	-0.0202	1.7794	-0.0989
C	1.3414	1.3361	-0.2157	C	1.2795	1.3792	0.0831
S	0.3807	-1.1915	-0.8755	S	0.4709	-1.2951	-0.1418
O	2.2249	2.1779	-0.4015	O	2.1692	2.2131	-0.1052
C	1.6353	-0.1737	-0.0322	C	1.5483	-0.0459	0.6393
O	2.9276	-0.5637	-0.4847	O	2.8927	-0.5046	0.7334
C	3.9697	-0.3155	0.4543	C	3.6427	-0.4281	-0.4759
C	4.121	-1.4917	1.4029	C	4.9247	-1.2165	-0.2819
O	-4.618	-1.1878	0.3174	O	-4.6141	-1.3265	0.0142
H	-4.3906	1.3246	0.8474	H	-4.5435	1.3452	-0.0376
H	-2.3171	2.6561	0.6943	H	-2.4849	2.6816	-0.0884
H	-2.2665	-2.178	-0.4482	H	-2.2269	-2.2733	-0.0164
H	-0.0391	2.7964	0.0041	H	-0.1244	2.7752	-0.3069
H	1.5367	-0.3882	1.039	H	1.2095	0.0055	1.6818
H	4.8964	-0.1998	-0.1172	H	3.0829	-0.8462	-1.3182
H	3.8048	0.616	1.0063	H	3.8974	0.6117	-0.6966
H	4.9703	-1.3393	2.0751	H	5.5439	-1.1821	-1.1826
H	3.2207	-1.6325	2.0086	H	5.5009	-0.816	0.5588
H	4.2762	-2.4195	0.8425	H	4.7015	-2.2624	-0.0463
H	-4.4696	-2.1081	0.042	H	-5.4009	-0.7568	0.0086
**Conf 13**	**X axis(Å)**	**Y axis(Å)**	**Z axis(Å)**	**Conf 14**	**X axis(Å)**	**Y axis(Å)**	**Z axis(Å)**
C	-3.3057	-0.3216	-0.3257	C	3.2276	-0.0046	0.4193
C	-3.1783	0.9974	-0.7456	C	2.9679	1.3585	0.3336
C	-1.9578	1.6546	-0.5777	C	1.7079	1.7906	-0.0854
C	-0.8684	0.9852	-0.0077	C	0.7111	0.8593	-0.3988
C	-1.0081	-0.3431	0.4219	C	0.9846	-0.5145	-0.3193
C	-2.2372	-0.9915	0.2616	C	2.2525	-0.9404	0.0896
N	0.3251	1.7022	0.1585	N	-0.5278	1.349	-0.8364
C	1.5375	1.1728	0.5193	C	-1.6707	0.6101	-1.0007

S	0.3039	-1.2004	1.269	S	-0.2034	-1.7375	-0.8342
O	2.478	1.9392	0.7511	O	-2.6647	1.1484	-1.4984
C	1.7179	-0.353	0.5076	C	-1.7236	-0.8215	-0.431
O	1.8992	-0.7979	-0.8382	O	-1.9452	-0.8524	0.9776
C	3.1688	-1.3933	-1.0856	C	-3.2591	-0.453	1.3598
C	4.268	-0.351	-1.1892	C	-3.1918	0.8781	2.0843
O	-4.4805	-0.9994	-0.4643	O	4.4454	-0.4705	0.8171
H	-4.0106	1.5276	-1.1982	H	3.7274	2.0932	0.5823
H	-1.8655	2.6868	-0.9054	H	1.5127	2.8577	-0.1535
H	-2.3722	-2.0167	0.597	H	2.4902	-1.9997	0.1478
H	0.3289	2.7134	0.0149	H	-0.6216	2.3385	-1.0727
H	2.5892	-0.586	1.1309	H	-2.533	-1.36	-0.9378
H	3.3977	-2.1451	-0.3217	H	-3.9474	-0.4014	0.5087
H	3.0906	-1.919	-2.043	H	-3.6421	-1.215	2.0465
H	5.2094	-0.8137	-1.4993	H	-4.1816	1.1758	2.4416
H	3.998	0.4238	-1.9141	H	-2.5116	0.8163	2.9403
H	4.4336	0.149	-0.2304	H	-2.8061	1.6647	1.4291
H	-5.1309	-0.421	-0.8954	H	5.0213	0.2835	1.0247
**Conf 15**	**X axis(Å)**	**Y axis(Å)**	**Z axis(Å)**	**Conf16**	**X axis(Å)**	**Y axis(Å)**	**Z axis(Å)**
C	-3.4421	-0.5087	0.2229	C	-3.4833	-0.3244	0.2789
C	-3.4687	0.8456	0.5344	C	-3.4109	1.0569	0.4058
C	-2.2918	1.5917	0.4452	C	-2.1883	1.7045	0.2266
C	-1.0938	0.9767	0.0629	C	-1.0377	0.9628	-0.0597
C	-1.0779	-0.3875	-0.2611	C	-1.1182	-0.4313	-0.1941
C	-2.2627	-1.1266	-0.1803	C	-2.3515	-1.074	-0.027
N	0.0519	1.7785	-0.0281	N	0.1601	1.6634	-0.2535
C	1.3396	1.3387	-0.2095	C	1.4143	1.1167	-0.3679
S	0.3863	-1.1884	-0.8791	S	0.2873	-1.4106	-0.6796
O	2.2214	2.1845	-0.3855	O	2.3493	1.8676	-0.6589
C	1.6367	-0.1711	-0.0319	C	1.6065	-0.3678	0.0204
O	2.9314	-0.5522	-0.4861	O	2.8723	-0.8785	-0.3863
C	3.9702	-0.3149	0.4592	C	3.7788	-1.1303	0.6818
C	4.1191	-1.5022	1.3942	C	4.3447	0.1493	1.2711
O	-4.5676	-1.2745	0.291	O	-4.7004	-0.9123	0.4538
H	-4.3881	1.3352	0.8409	H	-4.306	1.6287	0.6363
H	-2.3211	2.6517	0.6848	H	-2.1484	2.7866	0.3207
H	-2.278	-2.1823	-0.4406	H	-2.4203	-2.1515	-0.1466
H	-0.0431	2.7959	0.0055	H	0.1343	2.6802	-0.3603
H	1.5388	-0.3899	1.0385	H	1.4998	-0.4212	1.1117
H	4.8986	-0.1918	-0.1078	H	3.2995	-1.746	1.4515
H	3.8027	0.61	1.0213	H	4.6022	-1.7204	0.2657
H	4.9661	-1.3572	2.071	H	5.11	-0.0774	2.0191
H	3.217	-1.6509	1.9952	H	4.7907	0.7704	0.4877

H	4.2769	-2.4232	0.8234	H	3.5635	0.7459	1.7517
H	-5.3168	-0.7171	0.5584	H	-4.6146	-1.8691	0.3059

Table S2 The Cartesian coordinates of the lowest energy conformers for (8*R*)-**2**.

Conf 1	X axis(Å)	Y axis(Å)	Z axis(Å)	Conf 2	X axis(Å)	Y axis(Å)	Z axis(Å)
O	-4.2787	-1.056	-1.1942	O	-3.8131	1.9904	-1.1752
C	-3.242	-0.791	-0.5776	C	-2.9726	1.1544	-0.829
N	-2.0416	-1.2533	-1.0432	N	-1.7788	1.5725	-0.3081
C	-0.8025	-1.1414	-0.3939	C	-0.6822	0.7566	0.0099
C	0.3472	-1.4927	-1.1107	C	0.3807	1.3326	0.7153
O	0.2074	-1.9109	-2.4087	O	0.2892	2.6523	1.0752
C	1.608	-1.4143	-0.5246	C	1.5096	0.5867	1.0444
C	1.7313	-1.0008	0.8087	C	1.5945	-0.7567	0.6541
C	3.0972	-0.8906	1.4483	C	2.8267	-1.5686	0.9846
C	3.571	0.5592	1.6048	C	3.7569	-1.7678	-0.2178
N	3.6653	1.2738	0.3452	N	4.2556	-0.5238	-0.7747
C	4.6943	1.0611	-0.5404	C	5.2469	0.2051	-0.1626
C	4.6039	1.8983	-1.7866	C	5.6185	1.4617	-0.9007
O	5.587	0.2398	-0.3625	O	5.7604	-0.1068	0.906
C	0.5747	-0.6801	1.5377	C	0.5382	-1.3279	-0.0739
C	-0.6919	-0.7444	0.9423	C	-0.6004	-0.5783	-0.3983
S	-2.1275	-0.4204	1.9447	S	-1.8518	-1.3208	-1.4251
C	-3.2466	0.1664	0.6321	C	-3.2851	-0.3565	-0.8437
O	-2.9001	1.5044	0.2725	O	-3.7038	-0.8449	0.4302
C	-3.9483	2.1714	-0.4267	C	-5.0245	-0.4299	0.7698
C	-3.5011	3.5935	-0.7041	C	-5.3891	-1.0557	2.1019
H	-2.0464	-1.7233	-1.9532	H	-1.6767	2.5758	-0.1287
H	1.0852	-2.1468	-2.7548	H	1.0584	2.8769	1.6262
H	2.5004	-1.6694	-1.0908	H	2.3326	1.0374	1.5936
H	3.0632	-1.3704	2.4345	H	3.3712	-1.0912	1.8085
H	3.8257	-1.4636	0.8616	H	2.5098	-2.5483	1.3638
H	2.8851	1.1274	2.2425	H	4.6164	-2.3769	0.0846
H	4.5582	0.5699	2.0806	H	3.2407	-2.2948	-1.0276
H	2.9178	1.9282	0.1037	H	3.8286	-0.1733	-1.6349
H	5.6042	2.037	-2.2057	H	5.5487	1.3139	-1.9821
H	3.9698	1.3882	-2.5166	H	4.9426	2.2672	-0.6015
H	4.181	2.8824	-1.5655	H	6.6474	1.7381	-0.6544
H	0.6624	-0.3824	2.5815	H	0.6063	-2.366	-0.3956
H	-4.251	0.1522	1.0713	H	-4.0771	-0.5306	-1.5816
H	-4.1553	1.6631	-1.3737	H	-5.0686	0.6604	0.8551
H	-4.8573	2.1831	0.1844	H	-5.7305	-0.7617	7.00E-04
H	-4.2757	4.1486	-1.2406	H	-6.3988	-0.766	2.406

H	-3.2753	4.1161	0.2314	H	-5.3375	-2.1479	2.0418
H	-2.5836	3.5993	-1.3018	H	-4.683	-0.7457	2.8794
**Conf 3**	**X axis(Å)**	**Y axis(Å)**	**Z axis(Å)**	**Conf 4**	**X axis(Å)**	**Y axis(Å)**	**Z axis(Å)**
O	-4.5205	0.5824	-0.4114	O	-4.0732	0.4491	-1.8286
C	-3.2984	0.4062	-0.4329	C	-3.0436	0.2219	-1.1856
N	-2.4641	1.4064	-0.0139	N	-2.1727	1.2438	-0.9226
C	-1.0629	1.4031	-0.0919	C	-0.9109	1.123	-0.3203
C	-0.3663	2.42	0.5723	C	-0.2434	2.2982	0.0453
O	-1.0856	3.3545	1.2715	O	-0.8635	3.4993	-0.1841
C	1.0253	2.4801	0.5337	C	1.0227	2.2541	0.6255
C	1.7404	1.526	-0.2018	C	1.6459	1.0172	0.8378
C	3.2528	1.5499	-0.2315	C	3.0336	0.9465	1.4363
C	3.875	0.777	0.9373	C	4.1388	1.0908	0.3839
N	3.6008	-0.6467	0.8831	N	4.1775	-0.0179	-0.5512
C	4.3365	-1.4979	0.0926	C	4.7992	-1.2052	-0.2427
C	3.8693	-2.9266	0.1428	C	4.6959	-2.2425	-1.3267
O	5.2657	-1.1318	-0.6185	O	5.3579	-1.4216	0.8269
C	1.0383	0.527	-0.8968	C	0.9845	-0.1602	0.45
C	-0.3591	0.4566	-0.8422	C	-0.2899	-0.1147	-0.1289
S	-1.1946	-0.7777	-1.8155	S	-1.0215	-1.6289	-0.7143
C	-2.6878	-0.9652	-0.7891	C	-2.7754	-1.1601	-0.5543
O	-2.3764	-1.7423	0.3682	O	-3.1634	-1.2204	0.8177
C	-3.5392	-2.2787	0.995	C	-4.5778	-1.2733	0.986
C	-3.0935	-3.1296	2.1683	C	-4.8736	-1.3953	2.4681
H	-2.9033	2.2341	0.3993	H	-2.4718	2.1871	-1.1865
H	-0.47	4.0019	1.6546	H	-0.3154	4.2108	0.1876
H	1.5604	3.2615	1.0659	H	1.5332	3.1699	0.9101
H	3.6063	1.1498	-1.19	H	3.1378	1.7458	2.1808
H	3.5919	2.5928	-0.1963	H	3.1466	0.0047	1.9875
H	4.9613	0.9212	0.9306	H	4.0036	2.0041	-0.2056
H	3.4927	1.1417	1.8969	H	5.1111	1.1545	0.8853
H	2.795	-1.0083	1.3985	H	3.6758	0.0732	-1.4373
H	3.5205	-3.1868	1.1462	H	3.7489	-2.7786	-1.2219
H	3.0522	-3.0626	-0.5707	H	4.741	-1.7776	-2.3157
H	4.6974	-3.5905	-0.1198	H	5.5265	-2.9479	-1.2367
H	1.5917	-0.2028	-1.4871	H	1.476	-1.1218	0.5951
H	-3.4098	-1.506	-1.4122	H	-3.3349	-1.9084	-1.1279
H	-4.1828	-1.4699	1.3548	H	-5.04	-0.3611	0.5958
H	-4.0972	-2.8959	0.2825	H	-4.9854	-2.1409	0.4557
H	-3.954	-3.5659	2.6831	H	-5.9512	-1.4406	2.6486
H	-2.4362	-3.9378	1.8308	H	-4.4077	-2.296	2.8814
H	-2.5202	-2.5302	2.8832	H	-4.4589	-0.5422	3.0154

Conf 5	X axis(Å)	Y axis(Å)	Z axis(Å)	Conf 6	X axis(Å)	Y axis(Å)	Z axis(Å)
O	-4.6774	1.4326	-0.0142	O	-4.6779	1.4319	-0.0115
C	-3.5827	0.8883	-0.1895	C	-3.5832	0.888	-0.1878
N	-2.4345	1.5629	0.1225	N	-2.435	1.5626	0.1244
C	-1.1241	1.1316	-0.1344	C	-1.1245	1.1318	-0.1336
C	-0.0715	1.859	0.4336	C	-0.0719	1.8589	0.4347
O	-0.3728	2.9382	1.2236	O	-0.373	2.9372	1.2258
C	1.2537	1.4957	0.2076	C	1.2533	1.496	0.2077
C	1.5435	0.4009	-0.6179	C	1.5429	0.402	-0.619
C	2.9758	-0.0112	-0.853	C	2.9752	-0.0096	-0.8552
C	3.481	-0.9506	0.2447	C	3.4817	-0.9485	0.2424
N	4.8756	-1.304	0.0634	N	4.876	-1.3024	0.06
C	5.8871	-0.4439	0.4258	C	5.888	-0.4377	0.4098
C	7.2668	-0.9859	0.171	C	7.2673	-0.9933	0.1838
O	5.6934	0.6773	0.8837	O	5.6954	0.6769	0.8838
C	0.4889	-0.3146	-1.2065	C	0.4882	-0.3131	-1.2078
C	-0.8446	0.045	-0.9691	C	-0.8452	0.046	-0.9694
S	-2.1345	-0.8297	-1.8311	S	-2.1353	-0.8281	-1.8316
C	-3.4784	-0.5885	-0.6242	C	-3.4787	-0.5883	-0.6239
O	-3.2943	-1.4769	0.478	O	-3.2939	-1.4777	0.4773
C	-4.476	-1.6327	1.2598	C	-4.4753	-1.6346	1.2595
C	-4.1902	-2.6449	2.352	C	-4.1888	-2.6478	2.3506
H	-2.5395	2.4678	0.5909	H	-2.54	2.467	0.5936
H	0.458	3.3229	1.5522	H	0.4579	3.3216	1.5545
H	2.0689	2.0543	0.6618	H	2.0686	2.0544	0.662
H	3.0684	-0.4975	-1.8328	H	3.0673	-0.496	-1.8348
H	3.6029	0.8872	-0.9146	H	3.6018	0.8892	-0.9174
H	3.3673	-0.4898	1.2332	H	3.3694	-0.4872	1.2309
H	2.9086	-1.8849	0.2466	H	2.909	-1.8826	0.2456
H	5.1055	-2.2199	-0.3275	H	5.1057	-2.2262	-0.312
H	7.2952	-2.0687	0.3224	H	7.5686	-1.5747	1.0591
H	7.5585	-0.7569	-0.8573	H	7.2892	-1.6346	-0.7019
H	7.974	-0.5208	0.8632	H	7.9712	-0.1702	0.033
H	0.7086	-1.1556	-1.8623	H	0.7078	-1.1534	-1.8645
H	-4.4016	-0.8501	-1.1545	H	-4.4021	-0.8496	-1.1541
H	-4.7586	-0.6775	1.7134	H	-4.7579	-0.6799	1.7141
H	-5.2972	-1.9916	0.6299	H	-5.2967	-1.9931	0.6296
H	-5.0713	-2.7998	2.9809	H	-5.0696	-2.8034	2.9798
H	-3.8916	-3.6054	1.919	H	-3.8901	-3.6077	1.9166
H	-3.3606	-2.3079	2.9823	H	-3.3589	-2.3111	2.9809
**Conf 7**	**X axis(Å)**	**Y axis(Å)**	**Z axis(Å)**				

O	-4.4674	1.486	-1.1003				
C	-3.4578	0.8422	-0.7977				
N	-2.3037	1.5045	-0.4805				
C	-1.0513	0.9187	-0.2403				
C	-0.031	1.7358	0.2606				
O	-0.3098	3.0555	0.5059				
C	1.2413	1.2245	0.5042				
C	1.5129	-0.1233	0.2325				
C	2.8954	-0.6791	0.4698				
C	3.8092	-0.4607	-0.7385				
N	5.1594	-0.9301	-0.4949				
C	6.0588	-0.1827	0.2308				
C	7.4102	-0.8275	0.3733				
O	5.785	0.8954	0.7473				
C	0.4987	-0.9351	-0.2994				
C	-0.7839	-0.422	-0.5346				
S	-1.9963	-1.4686	-1.3136				
C	-3.4994	-0.6917	-0.6362				
O	-3.6779	-1.1003	0.7196				
C	-5.0002	-0.8612	1.1953				
C	-5.0973	-1.3956	2.611				
H	-2.358	2.5247	-0.4081				
H	0.4858	3.4766	0.8743				
H	2.0305	1.8609	0.8979				
H	3.321	-0.2129	1.3675				
H	2.8313	-1.7501	0.7016				
H	3.4308	-1.0039	-1.6116				
H	3.8554	0.6023	-1.0036				
H	5.4469	-1.83	-0.8847				
H	7.4002	-1.4952	1.2388				
H	7.6661	-1.4005	-0.5225				
H	8.1691	-0.0537	0.518				
H	0.7115	-1.9761	-0.5373				
H	-4.328	-1.0725	-1.2448				
H	-5.214	0.2122	1.1957				
H	-5.7267	-1.375	0.5564				
H	-6.0983	-1.2352	3.0209				
H	-4.8728	-2.4671	2.6349				
H	-4.3661	-0.9023	3.2601				

Table S3 The Cartesian coordinates of the lowest energy conformers for (7*R*)-**3**

**Conf 1**	**X axis(Å)**	**Y axis(Å)**	**Z axis(Å)**	**Conf 2**	**X axis(Å)**	**Y axis(Å)**	**Z axis(Å)**

O	1.9789	3.2723	-1.1155	O	-5.6653	-0.7342	-0.5814
C	1.9314	2.6416	-0.0613	C	-4.4748	-1.0368	-0.608
N	2.6007	1.4613	0.051	N	-3.5502	-0.1246	-1.0197
C	2.6314	0.5249	1.1698	C	-2.0984	-0.2636	-1.0609
C	1.9375	-0.824	0.858	C	-1.433	0.2902	0.218
O	2.5574	-1.3876	-0.3109	O	-1.6153	1.7168	0.2295
C	2.3881	-2.8019	-0.4033	C	-1.5652	2.267	1.5454
C	3.042	-3.2739	-1.6868	C	-1.7171	3.7712	1.4363
H	2.1289	-1.4935	1.7076	H	-1.9303	-0.1421	1.0966
C	0.4356	-0.6563	0.6776	C	0.0479	-0.0533	0.2283
C	-0.1078	-0.2935	-0.573	C	0.9676	0.7008	-0.5308
C	-1.4979	-0.1202	-0.6905	C	2.3274	0.3434	-0.5066
C	-2.3521	-0.299	0.397	C	2.7867	-0.7397	0.2426
C	-1.7887	-0.6611	1.6369	C	1.8492	-1.4825	0.9879
O	-2.587	-0.8455	2.7414	O	2.2497	-2.5616	1.7403
C	-0.4138	-0.8361	1.7765	C	0.4977	-1.1451	0.9807
N	-3.7037	-0.0964	0.1501	N	4.1514	-0.9884	0.1796
C	-3.859	0.2352	-1.1124	C	4.7102	-0.0973	-0.6086
S	-2.4372	0.3198	-2.0655	S	3.6675	1.0672	-1.3117
C	1.1546	3.1367	1.1266	C	-3.9904	-2.4016	-0.2028
H	3.094	1.1767	-0.8003	H	-3.9423	0.7908	-1.2584
H	3.6949	0.3394	1.3584	H	-1.8406	-1.3161	-1.2142
H	2.2027	0.982	2.0658	H	-1.7674	0.2995	-1.9412
H	1.3244	-3.061	-0.4149	H	-0.6099	2.0255	2.0231
H	2.866	-3.2857	0.4554	H	-2.3828	1.8551	2.147
H	2.9431	-4.3571	-1.7998	H	-1.6898	4.239	2.4243
H	4.1054	-3.0129	-1.6968	H	-2.6631	4.029	0.9488
H	2.5856	-2.7847	-2.5537	H	-0.916	4.1954	0.8219
H	0.538	-0.1396	-1.4351	H	0.6344	1.5528	-1.1196
H	-3.4965	-0.6735	2.4215	H	3.2182	-2.6143	1.6056
H	-0.019	-1.1112	2.7517	H	-0.1938	-1.7435	1.5696
H	-4.8317	0.4457	-1.5381	H	5.7738	-0.0915	-0.8096
H	0.6733	4.0868	0.877	H	-4.8287	-2.9844	0.1897
H	0.3792	2.417	1.3994	H	-3.578	-2.922	-1.0713
H	1.8282	3.2997	1.972	H	-3.2306	-2.3198	0.5786
**Conf 3**	**X axis(Å)**	**Y axis(Å)**	**Z axis(Å)**	**Conf 4**	**X axis(Å)**	**Y axis(Å)**	**Z axis(Å)**
O	-5.7893	-0.7362	-0.517	O	5.6254	0.7739	-0.7726
C	-4.6306	-1.1397	-0.5848	C	4.4941	0.3412	-0.9869
N	-3.5975	-0.2836	-0.3516	N	3.4238	1.1689	-0.8241
C	-2.1659	-0.5483	-0.4371	C	2.005	0.8502	-0.9516
C	-1.3659	0.3415	0.5382	C	1.3497	0.5261	0.4112
O	-1.6007	1.715	0.18	O	1.8147	-0.7587	0.8513

C	-1.3596	2.6159	1.2609	C	1.7745	-0.9056	2.2698
C	-1.5993	4.0274	0.7628	C	2.2457	-2.3046	2.6133
H	-1.7424	0.1719	1.556	H	1.654	1.2902	1.1394
C	0.115	0.0096	0.4561	C	-0.1647	0.5068	0.2709
C	0.909	0.518	-0.5938	C	-0.8214	-0.6135	-0.2814
C	2.2727	0.1789	-0.6438	C	-2.2218	-0.5904	-0.4067
C	2.8565	-0.6507	0.3133	C	-2.9749	0.5126	-0.0048
C	2.0429	-1.152	1.3492	C	-2.2974	1.6224	0.5386
O	2.569	-1.9767	2.3157	O	-2.9931	2.7359	0.9474
C	0.69	-0.8276	1.4197	C	-0.9109	1.6208	0.6741
N	4.2109	-0.9099	0.1494	N	-4.3482	0.4097	-0.1837
C	4.6386	-0.2775	-0.9205	C	-4.6216	-0.7619	-0.713
S	3.4789	0.6443	-1.7824	S	-3.2886	-1.7925	-1.027
C	-4.3016	-2.571	-0.9065	C	4.2564	-1.0701	-1.4469
H	-3.8825	0.6896	-0.2008	H	3.6877	2.1125	-0.5299
H	-1.9735	-1.607	-0.2373	H	1.5386	1.7351	-1.3991
H	-1.8842	-0.3411	-1.4762	H	1.8849	0.0146	-1.6481
H	-0.3281	2.5196	1.6154	H	0.7542	-0.757	2.6386
H	-2.0457	2.3928	2.0851	H	2.437	-0.1675	2.7348
H	-1.4316	4.7565	1.5604	H	2.2379	-2.4647	3.695
H	-2.6241	4.1371	0.3929	H	3.2607	-2.4726	2.2379
H	-0.9319	4.2588	-0.0739	H	1.603	-3.0543	2.14
H	0.4775	1.1708	-1.3497	H	-0.2565	-1.4881	-0.5966
H	3.5148	-2.0675	2.0789	H	-3.932	2.5236	0.7677
H	0.0961	-1.2352	2.2348	H	-0.426	2.497	1.0984
H	5.6688	-0.3307	-1.2486	H	-5.6339	-1.0687	-0.9427
H	-5.2177	-3.1046	-1.1757	H	5.2161	-1.587	-1.5408
H	-3.6113	-2.6204	-1.7528	H	3.6441	-1.6124	-0.7247
H	-3.8566	-3.0545	-0.0329	H	3.7687	-1.0685	-2.4251
**Conf 5**	**X axis(Å)**	**Y axis(Å)**	**Z axis(Å)**	**Conf 6**	**X axis(Å)**	**Y axis(Å)**	**Z axis(Å)**
O	-2.9473	2.9079	-0.6422	O	-5.4659	-1.4809	-0.1707
C	-3.1691	1.7075	-0.7937	C	-4.2564	-1.5332	-0.3798
N	-2.2327	0.9267	-1.401	N	-3.5939	-0.4306	-0.8288
C	-2.2429	-0.5195	-1.5961	C	-2.1635	-0.2691	-1.0695
C	-1.4932	-1.3199	-0.4994	C	-1.4432	0.3408	0.1534
O	-2.1772	-1.1518	0.7509	O	-1.8821	1.7027	0.2898
C	-1.9217	-2.2066	1.6771	C	-1.711	2.2116	1.6114
C	-2.6624	-1.8951	2.9625	C	-2.175	3.6546	1.6275
H	-1.5515	-2.3805	-0.7798	H	-1.7329	-0.2185	1.0529
C	-0.0308	-0.9068	-0.4008	C	0.0638	0.2816	-0.0403
C	0.3607	0.1749	0.417	C	0.8136	-0.6976	0.643
C	1.7184	0.5357	0.47	C	2.2056	-0.7501	0.4431

C	2.686	-0.1462	-0.2672	C	2.8589	0.136	-0.4134
C	2.2726	-1.2202	-1.0807	C	2.0889	1.1039	-1.0886
O	3.1869	-1.9229	-1.8302	O	2.6848	1.9988	-1.9454
C	0.9326	-1.5949	-1.1486	C	0.7103	1.1752	-0.9048
N	3.9907	0.3089	-0.1281	N	4.2341	-0.0255	-0.5214
C	3.9991	1.3251	0.7058	C	4.6083	-1.0226	0.2484
S	2.4871	1.7909	1.3646	S	3.3718	-1.8167	1.1302
C	-4.46	1.0764	-0.3516	C	-3.4646	-2.7926	-0.1599
H	-1.3906	1.4413	-1.6715	H	-4.1843	0.3992	-0.9381
H	-3.2814	-0.8603	-1.6449	H	-1.7311	-1.2382	-1.3371
H	-1.7803	-0.6997	-2.5729	H	-2.0777	0.394	-1.938
H	-0.8489	-2.288	1.8801	H	-0.6579	2.1593	1.907
H	-2.2817	-3.1545	1.2628	H	-2.3111	1.6221	2.3131
H	-2.5027	-2.6814	3.7055	H	-2.0672	4.0882	2.6257
H	-3.7369	-1.7985	2.7748	H	-3.2243	3.7258	1.3224
H	-2.3248	-0.9405	3.3794	H	-1.5953	4.2534	0.9173
H	-0.3767	0.7255	0.9984	H	0.3344	-1.4031	1.3169
H	4.0473	-1.4979	-1.6348	H	3.6374	1.7759	-1.9188
H	0.6551	-2.4282	-1.7899	H	0.1449	1.9393	-1.4352
H	4.9097	1.8475	0.9694	H	5.6422	-1.3334	0.3272
H	-5.0982	1.8384	0.1055	H	-4.1094	-3.5556	0.2858
H	-4.9856	0.6571	-1.2135	H	-3.0886	-3.1663	-1.1159
H	-4.2738	0.2959	0.388	H	-2.6308	-2.6042	0.5212
**Conf 7**	**X axis(Å)**	**Y axis(Å)**	**Z axis(Å)**	**Conf 8**	**X axis(Å)**	**Y axis(Å)**	**Z axis(Å)**
O	5.7019	-1.2745	-0.0504	O	5.5726	0.6055	-0.7757
C	4.5333	-1.4911	0.2611	C	4.3838	0.9153	-0.7913
N	3.5984	-0.511	0.12	N	3.446	-0.0051	-1.1515
C	2.1843	-0.5515	0.4756	C	1.9941	0.1394	-1.1618
C	1.3461	0.3741	-0.4326	C	1.3624	-0.3479	0.16
O	1.8077	1.7215	-0.2369	O	1.5451	-1.7721	0.2392
C	1.4612	2.5912	-1.314	C	1.6217	-2.2682	1.575
C	1.981	3.9775	-0.9891	C	3.0404	-2.1598	2.1056
H	1.5244	0.0904	-1.4786	H	1.8727	0.1379	1.0008
C	-0.1305	0.2484	-0.0936	C	-0.1203	-0.0125	0.1908
C	-0.9725	-0.5141	-0.9288	C	-1.0566	-0.818	-0.4911
C	-2.3345	-0.6327	-0.5951	C	-2.4175	-0.4657	-0.4511
C	-2.8692	-0.0209	0.5386	C	-2.8621	0.6615	0.2395
C	-2.0082	0.7328	1.3608	C	-1.9086	1.455	0.9082
O	-2.4857	1.3536	2.4907	O	-2.2943	2.5788	1.6004
C	-0.6573	0.865	1.0492	C	-0.5558	1.1237	0.8838
N	-4.2271	-0.2189	0.7529	N	-4.2296	0.8998	0.1996
C	-4.706	-0.9713	-0.2123	C	-4.8049	-0.0429	-0.5132

S	-3.5926	-1.4821	-1.4109	S	-3.7752	-1.2456	-1.1695
C	4.0817	-2.826	0.7852	C	3.9155	2.2973	-0.4277
H	3.9784	0.3951	-0.1729	H	3.8282	-0.9311	-1.3638
H	1.8196	-1.5817	0.4148	H	1.7365	1.1847	-1.3589
H	2.128	-0.2304	1.5221	H	1.6374	-0.464	-2.0045
H	0.3744	2.626	-1.4428	H	1.3364	-3.3247	1.54
H	1.9216	2.2311	-2.2404	H	0.9085	-1.7569	2.231
H	1.7433	4.6815	-1.7914	H	3.1179	-2.6164	3.0965
H	3.0664	3.9586	-0.8452	H	3.3608	-1.1163	2.18
H	1.5422	4.3442	-0.0552	H	3.742	-2.6601	1.43
H	-0.5861	-1.0043	-1.8188	H	-0.7352	-1.7045	-1.0337
H	-3.4399	1.137	2.5122	H	-3.2665	2.6181	1.4907
H	-0.0199	1.4599	1.701	H	0.1483	1.7621	1.4128
H	-5.7487	-1.2588	-0.2557	H	-5.8737	-0.0663	-0.6829
H	4.9527	-3.4659	0.9532	H	4.7659	2.8907	-0.0798
H	3.5554	-2.7022	1.7354	H	3.4831	2.7856	-1.305
H	3.4261	-3.3082	0.0553	H	3.1756	2.2499	0.3754

.
